# Heterogeneous associations of retirement with health and behaviors: a longitudinal study in 35 countries

**DOI:** 10.1093/aje/kwaf126

**Published:** 2025-06-13

**Authors:** Koryu Sato, Haruko Noguchi

**Affiliations:** Faculty of Policy Management, Keio University, Fujisawa-shi, Kanagawa 252-0882, Japan; Graduate School of Economics, Waseda University, Tokyo 169-8050, Japan; Department of Social Epidemiology, Graduate School of Medicine and School of Public Health, Kyoto University, Kyoto 606-8315, Japan; Graduate School of Economics, Waseda University, Tokyo 169-8050, Japan; Waseda Institute of Social and Human Capital Studies (WISH), Tokyo 162-0041, Japan

**Keywords:** retirement, cognitive function, physical independence, self-rated health, physical inactivity, smoking, binge drinking

## Abstract

Many developed countries are raising their state pension age (SPA), thereby delaying retirement. However, existing evidence on the impact of retirement on health yields inconsistent results. This study aims to explore heterogeneous associations of retirement with health and behaviors using harmonized datasets of the Health and Retirement Study and its sister surveys in 35 countries. The data comprises 396 904 observations from 106 927 individuals aged 50-70 years. On average, participants were followed up for 6.7 years, and 50.5% of them consisted of men. This study employed the SPA of each country as an instrument for retirement and performed fixed-effects instrumental variable (IV) regression. Among women, retirement was associated with a 0.100 SD increase in cognitive function and a 3.8%-point increase in physical independence. In both genders, retirement was associated with increased self-rated health, with women indicating a larger point estimate than men. Additionally, retirement was associated with a 4.3%-point decrease in physical inactivity and a 1.9%-point decrease in smoking among women, while no such associations were observed among men. Heterogeneity was not found across countries, educational levels, and pre-retirement job characteristics. Gender differences in post-retirement health behaviors may contribute to heterogeneous associations between retirement and health.

**This article is part of a Special Collection on Cross-National Gerontology**.

## Introduction

The global population of people aged over 60 years is projected to undergo a 2-fold increase between 2015 and 2050.^1^ To confront the challenges posed by global aging, many developed countries are raising their state pension age (SPA).^2^ These policy adjustments may influence population health by delaying retirement and modifying budget constraints and time allocations for health investments in later life.^3^

The implications of delayed retirement on health remain uncertain, with a lack of consensus on this matter. While several studies have suggested an association between retirement and diminished cognitive functions,^4^ others have found no evidence of such an association^5^^,^^6^ and some have even reported a beneficial correlation.^7^ Discrepancies in the findings of studies examining the association between retirement and physical function are also evident.^4^ In contrast, the literature consistently demonstrates a beneficial association of retirement with self-rated health.^4^ However, this persistence of diverse outcomes across health dimensions remains puzzling, given that self-rated health constitutes a strong predictor of both cognitive and physical impairments.^8^

We hypothesized three potential sources of inconsistency in the previous literature. First, variations in statistical methodologies may contribute to these inconsistencies, especially since retirement is an endogenous decision influenced by factors such as health status.^9^ The tendency for people with deteriorating health to retire earlier may create reverse causality, leading to a spurious association that incorrectly suggests retirement has a detrimental effect on health. Second, the country of the study population may introduce another source of inconsistency. From a life-course perspective, individuals’ reactions to retirement often align with features of pension systems and social welfare policies that shape retirement decisions.^10^ However, most previous studies have been confined to a single country or region, limiting the evaluation of country-level heterogeneity. Third, inconsistencies may be attributable to individual-level heterogeneity based on demographic and occupational characteristics. If a subgroup adversely influenced by retirement conceals its beneficial effect in other subgroups, the overall average treatment effect of retirement in the population becomes unclear.

To provide a comprehensive perspective, we examine the association of retirement with cognitive function, physical independence, and self-rated health using harmonized longitudinal data derived from 35 countries. We used SPA as an instrumental variable (IV) for retirement to mitigate the potential endogenous bias and incorporated fixed effects (FEs) to account for unobserved characteristics at both individual and country levels. This study also explores the association of retirement with physical inactivity, smoking, and binge drinking to unveil underlying mechanisms linking retirement to health outcomes. Furthermore, we assess the heterogeneity of the associations across various dimensions, including countries, gender, education, and pre-retirement job characteristics.

## Methods

### Data and study participants

We used the harmonized datasets of the Health and Retirement Study (HRS) and its sister surveys provided by the Gateway to Global Aging Data project (Table 1).^11^ The surveys were designed to represent the national older population and consisted of 50.5% men. Participants were interviewed basically every 2 years and followed up for 6.7 years on average.

**Table 1:** Cohort characteristics of the surveys.

**Survey**	**Country**	**Interview years**	**No. of unique individuals**	**Mean follow-up years**	**Mean no. of interviews**	**% of men**
SHARE	Austria	2004, 2006, 2011, 2013, 2015, 2017, 2019	2877	5.4	3.3	46.2
	Belgium	2004, 2006, 2011, 2013, 2015, 2017, 2019	4118	5.7	3.3	51.8
	Bulgaria	2017, 2019	377	2.0	2.0	43.0
	Croatia	2015, 2017, 2019	1119	2.8	2.4	49.5
	Cyprus	2017, 2019	124	2.0	2.0	42.7
	Czech Republic	2006, 2011, 2013, 2015, 2017, 2019	3827	5.8	3.4	41.4
	Denmark	2004, 2006, 2011, 2013, 2015, 2017, 2019	3031	6.6	3.5	48.2
ELSA	England	2002, 2004, 2006, 2008, 2010, 2012, 2014, 2016, 2018	9895	7.7	4.5	47.6
SHARE	Estonia	2011, 2013, 2015, 2017, 2019	3662	4.8	3.2	42.3
	Finland	2017, 2019	550	2.0	2.0	47.3
	France	2004, 2006, 2011, 2013, 2015, 2017, 2019	3540	6.4	3.4	47.1
	Germany	2004, 2006, 2011, 2013, 2015, 2017, 2019	3437	5.3	3.2	49.7
	Greece	2004, 2006, 2015, 2017, 2019	2187	6.5	2.7	60.5
	Hungary	2011, 2017, 2019	788	6.4	2.3	41.1
	Israel	2004, 2006, 2013, 2015, 2017, 2019	1447	8.2	3.3	46.9
	Italy	2004, 2006, 2011, 2013, 2015, 2017, 2019	3026	5.9	3.2	56.1
	Latvia	2017, 2019	303	2.0	2.0	41.6
	Lithuania	2017, 2019	528	2.0	2.0	37.1
	Luxembourg	2013, 2015, 2017, 2019	841	3.9	2.8	54.5
	Malta	2017, 2019	239	2.0	2.0	72.8
	Netherlands	2004, 2006, 2011, 2013, 2019	1862	6.4	2.7	55.6
	Poland	2006, 2011, 2015, 2017, 2019	1700	5.6	2.7	41.4
	Portugal	2011, 2015, 2017	761	4.6	2.3	50.5
	Romania	2017, 2019	560	2.0	2.0	45.5
	Slovakia	2017, 2019	665	2.0	2.0	47.7
	Slovenia	2011, 2013, 2015, 2017, 2019	2531	4.5	3.1	44.6
	Spain	2004, 2006, 2011, 2013, 2015, 2017, 2019	2731	5.3	3.0	59.6
	Sweden	2004, 2006, 2011, 2013, 2015, 2017, 2019	3151	6.2	3.2	45.1
	Switzerland	2004, 2006, 2011, 2013, 2015, 2017, 2019	2178	6.4	3.6	48.8
CRELES	Costa Rica	2005, 2007, 2009, 2010, 2012	1244	2.3	2.1	76.9
MHAS	Mexico	2001, 2003, 2012, 2015, 2018	8148	6.8	2.7	66.7
HRS	United States	1992, 1994, 1996, 1998, 2000, 2002, 2004, 2006, 2008, 2010, 2012, 2014, 2016, 2018	25 753	9.2	5.2	46.9
CHARLS	China	2011, 2013, 2015, 2018	2819	4.8	2.9	54.1
JSTAR	Japan	2007, 2009, 2011	1775	3.0	2.5	64.8
KLoSA	South Korea	2006, 2008, 2010, 2012, 2014, 2016, 2018	5133	7.0	4.2	53.1
Overall			106 927	6.7	3.7	50.5

Abbreviations: CHARLS, China Health and Retirement Longitudinal Study; CRELES, Costa Rican Longevity and Healthy Aging Study; ELSA, English Longitudinal Study on Ageing; KLoSA, Korean Longitudinal Study of Aging; HRS, Health and Retirement Study; JSTAR, Japanese Study of Aging and Retirement; MHAS, Mexican Health and Aging Study; SHARE, Survey of Health, Ageing and Retirement in Europe.

**Figure 1:**
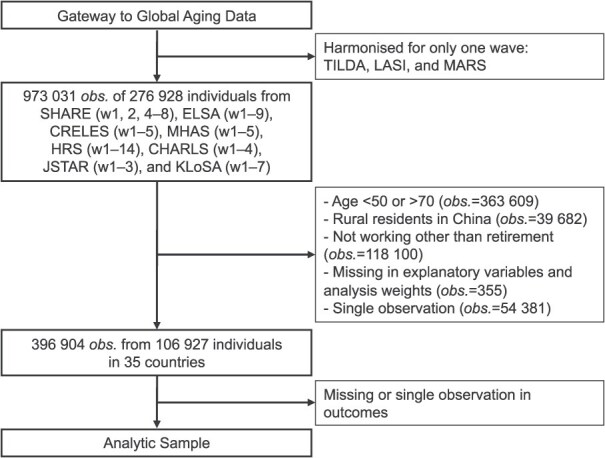
Flowchart of the analytic sample. Abbreviations: obs., observations; SHARE, Survey of Health, Ageing and Retirement in Europe; ELSA, English Longitudinal Study on Ageing; CRELES, Costa Rican Longevity and Healthy Aging Study; MHAS, Mexican Health and Aging Study; HRS, health and retirement study; CHARLS, China Health and Retirement Longitudinal Study; JSTAR, Japanese Study of Aging and Retirement; KLoSA, Korean Longitudinal Study of Aging; TILDA, the Irish Longitudinal Study on Ageing; LASI, Longitudinal Aging Study in India; MARS, Malaysia Ageing and Retirement Study.

Originally, the harmonized data involved 276 928 individuals with 973 031 observations. For the analysis, we focused on individuals aged 50-70 years, as their timings could be affected by the SPA of each country. Subsequently, we excluded certain individuals; for China, rural residents were omitted due to different pension systems in rural and urban areas^12^; individuals not working for reasons other than retirement (eg, unemployed, disabled, or homemakers) were excluded; observations with missing values were omitted; individuals observed only once were also excluded to prevent potential underestimation of standard errors in a FE model.^13^ Our datasets have an unbalanced panel structure; participants were included in the analysis as long as they were within the age range of 50-70 years in each wave. Observations from individuals outside this age range or those in periods of non-employment (eg, transitioning from employment to unemployment, disability, or homemaker status) were excluded. Consequently, our study encompassed 106 927 individuals with 396 904 observations in 35 countries (Figure 1). Table S1 in the Supplementary material compares the composition of gender and educational level by country between those included and excluded (ie, those who had never worked) in the analysis.

This study employed publicly available data that obtained informed consent from all participants and received ethical approval from relevant local ethics committees. Thus, the Ethics Committee of Kyoto University exempted this study from review. We followed the Strengthening the Reporting of Observational Studies in Epidemiology reporting guideline.^14^

### Outcomes

Cognitive function was measured using a word recall test (Appendix S1 in the Supplementary material).^15^ The test score was calculated by adding the number of words remembered during both the immediate and delayed recalls, as in previous studies.^5-7^^,^^16^^,^^17^ Typically, most surveys included a list of 10 words, providing a score range from 0 to 20, and their scores exhibited a normal distribution (Figure S1 in the Supplementary material). Waves 1 and 2 of the HRS comprised 20 words on the list, while the Mexican Health and Aging Study consisted of 8 words, and the Costa Rican Longevity and Healthy Aging Study and the Korean Longitudinal Study of Aging contained 3 words. Hence, the distribution of cognitive scores in Costa Rica and South Korea is skewed to the left (Figure S1 in the Supplementary material). To facilitate cross-country comparisons, we standardized the scores within each country by converting them to *z* scores (ie, mean of 0 and standard deviation of 1) using data pooled across all surveys conducted in that country.

The assessment of physical independence was based on the individual’s capability to carry out activities of daily living (ADL) and instrumental activities of daily living (IADL).^18^ To facilitate comparability, we selected 8 harmonized items, including four ADLs (bathing, eating, getting in and out of bed, and using the toilet) and 4 IADLs (managing money, taking medications, shopping for groceries, and preparing meals). Responses to the 8 items displayed high consistency (Cronbach’s α = .79). Most participants were capable of performing all 8 activities, as shown in Figure S2 in the Supplementary material. Hence, we dichotomized the participants and created a binary variable indicating 1 for those who were fully independent and 0 otherwise.

Self-rated health was measured using a 5-point Likert scale (1 = poor, 2 = fair, 3 = good, 4 = very good, and 5 = excellent). As shown in Figure S3 in the Supplementary material, while self-rated health was normally distributed in many countries, some countries exhibited skewed distributions. To enhance cross-country comparability, we standardized the data to *z* scores within each country.

Additionally, to explore the underlying mechanisms linking retirement to health outcomes, we examined the association between retirement and health behaviors, including physical inactivity, smoking, and binge drinking as these factors have been identified as potential risk factors for cognitive and physical impairments (Appendix S1 in the Supplementary material).^19^^,^^20^ Participants engaging in vigorous or moderate physical activity less than once per week were considered physically inactive individuals. Smoking status was categorized into current smokers and non-smokers. Binge drinking was defined as consuming 5 or more drinks per day for men and four or more for women.^21^ These three variables were converted into binary categories.

### Retirement status

The retirement statuses were determined using the harmonized variable of self-reported labor force status (Appendix S2 in the Supplementary material). Individuals who self-identified as retired during the interview, regardless of their working status (including those who were “partly retired”), were included in the retired group for comparison with workers, as defined in previous literature,^7^^,^^22^ and outlined in Table S2 in the Supplementary material. In other studies, retirement has been defined as not working.^5^^,^^16^ Considering this alternative definition, individuals who self-identified as retired but were still engaged in paid work were subsequently excluded in a sensitivity analysis.

### Instrumental variable

To address potential endogeneity in retirement decisions, we employed the SPA as an IV for retirement. To be valid, an IV must satisfy 2 conditions: (1) the relevance condition (the IV is associated with treatment, ie, retirement), and (2) the exclusion restriction (the IV has no association with potential outcomes under different values of treatment). The SPA is considered to meet these conditions and has been widely used as an IV for retirement in previous research.^17^ The SPA can influence retirement decisions even for those who do not participate in the pension system, as it serves as a reference point for retirement timing within a country.^23^ In some countries, early pensions are granted under specific circumstances, such as reduced benefits or sufficient social security contributions. Thus, we employed the joint IVs of the early retirement age (ERA) and the official retirement age (ORA) to predict retirement, following the approach of a previous study.^5^ A binary ERA variable indicated whether participants had reached the earliest age of eligibility for reduced pensions or full pensions with certain conditions. Similarly, a binary ORA variable indicated whether participants had reached the age of entitlement to minimum guaranteed pensions or full pensions without any requirements. In countries, where early pensions are not available, the ERA variable was set to 0 for all participants. We adjusted for variations in the SPA across gender and birth cohorts. Information on ERA, ORA, and their modifications during the study period was collected from the literature (Table S3 in the Supplementary material). We created a graph depicting age and the corresponding retirement rate by country, demonstrating changes in the retirement rate around the SPA (Figures S4-S5 in the Supplementary material).

### Other variables

We adjusted for age, age squared, and marital status (married/partnered or not). The model included age squared to account for a normal aging process, assuming that cognitive and physical function decline at an increasing rate with aging.^16^

To access potential heterogeneity, we performed interaction tests of retirement with country characteristics (region, country income, and the percentage of the older population), gender, educational levels, and pre-retirement job characteristics (physical demands, job control, and self-employment) (Appendix S3 in the Supplementary material). The effects of retirement on health may be modified by country-level factors, including economic development, social security systems, labor market conditions, and gender norms. Some studies have reported that retirement has greater negative impacts on health among men than women.^22^^,^^24^ Moreover, other studies have suggested heterogeneous associations between retirement and cognitive function based on educational levels^25-27^ and pre-retirement job characteristics.^28-30^

### Statistical analysis

We investigated the association between retirement and the outcomes using the FEIV models employing the 2-stage least squares procedure (Appendix S4 in the Supplementary material). Our model includes FEs of individual, country, year, and interactions between country and year. Robust standard errors, with clusters corresponding to the FEs, were estimated. All analyses were performed using Stata version 18.0 (StataCorp).

The FEIV model offers several advantages in estimating the potential causal effect of retirement on outcomes. By leveraging the panel data structure, individual FEs control for both observable and unobservable time-invariant factors, including genetic predisposition, educational attainment, and country-specific institutional and cultural characteristics. The country-by-year interaction FEs account for changes in sample composition across countries that arise from the unbalanced panel structure. Moreover, we applied the IV method to mitigate the endogeneity bias of retirement due to time-variant factors, such as health conditions. Assuming monotonicity (ie, the IV does not have conflicting effects on treatment in any individual), point estimates can be interpreted as a local average treatment effect (LATE) among “compliers” (ie, individuals who would retire upon reaching the SPA).

## Results

### Descriptive statistics

The datasets consist of 179 738 (45.3%) observations with a retired status (Table 2). We estimated the proportion of compliers in our analytic sample to be at least 52.8%, based on the observed retirement rates; 79.5% among those who had reached the ORA retired and 26.8% among those who had not reached the ORA. In comparison with workers, retirees were older and less likely to be men, married, highly educated, and to have experienced physical labor and a job with low control. To assess the potential impact of attrition bias, we compared characteristics between individuals who were followed up and those who were lost in the follow-up (Table S4 in the Supplementary material). Consequently, we confirmed almost no differences in characteristics between these two groups; however, we observed that those who were lost in the follow-up were, on average, 0.84 years older and had poorer self-rated health by 0.11 points than those who were followed up. If loss to follow-up was more prevalent among retirees and individuals with deteriorating health, this could bias the results in a way that overestimates the beneficial impact of retirement on health. Although the differences were not substantial, caution is necessary in interpreting our results.

**Table 2:** Descriptive statistics of observations by labor force status.

**Variables**	**Labor force status**	
	**Working (obs. = 217 166)**	**Retired (obs. = 179 738)**
Age, years, mean (SD)	57.9 (4.7)	64.2 (4.3)
Men, obs. (%)	113 377 (52.2)	83 958 (46.7)
Married, obs. (%)	173 581 (79.9)	136 461 (75.9)
Education, obs. (%)		
Low	50 673 (23.3)	53 159 (29.6)
Middle	93 304 (43.0)	83 463 (46.4)
High	55 364 (25.5)	35 754 (19.9)
Missing	17 825 (8.2)	7362 (4.1)
Physical demands, obs. (%)		
Physical labor	104 359 (48.1)	39 952 (22.2)
Non-physical labor	77 364 (35.6)	37 235 (20.7)
Missing	35 443 (16.3)	102 551 (57.1)
Job control, obs. (%)		
Low control	43 224 (19.9)	14 375 (8.0)
High control	78 541 (36.2)	30 100 (16.8)
Missing	95 401 (43.9)	135 263 (75.3)
Cognitive function, *z* score (SD)	0.17 (0.95)	-0.06 (0.99)
Missing or single observation	12 625 (5.8)	7003 (3.9)
Physical independence, obs. (%)		
Fully independent	186 649 (86.0)	148 587 (82.7)
Dependent	7959 (3.7)	19 778 (11.0)
Missing or single observation	22 558 (10.4)	11 373 (6.3)
Self-rated health, *z* score (SD)	0.26 (0.91)	−0.10 (0.99)
Missing or single observation	7299 (3.4)	4974 (2.8)
Physical inactivity, obs. (%)		
<1 per week	26 627 (12.3)	25 492 (14.2)
≥1 per week	116 137 (53.5)	104 568 (58.2)
Missing or single observation	74 402 (34.3)	49 678 (27.6)
Smoking, obs. (%)		
Currently smoking	37 588 (17.3)	25 688 (14.3)
Not smoking	146 405 (67.4)	114 838 (63.9)
Missing or single observation	33 173 (15.3)	39 212 (21.8)
Binge drinking, obs. (%)		
≥4/5 drinks per day	14 271 (6.6)	6449 (3.6)
<4/5 drinks per day	124 079 (57.1)	97 412 (54.2)
Missing or single observation	78 816 (36.3)	75 877 (42.2)

Abbreviation: Obs, observations.

### Country-level heterogeneity

Before pooling data from different countries, heterogeneity across countries was assessed using I^2^ statistics as an analogy to meta-analysis. Country-by-country analysis using the FEIV model did not show substantial heterogeneity, with I^2^ statistics around 40% or below for all the outcomes (Figures S6-S11 in the Supplementary material). Applying country FEs to cross-country pooling data, the interaction tests did not indicate any signs of heterogeneity according to region, country income, and the percentage of the older population (Tables S5-S7 in the Supplementary material). We also tested interaction terms between retirement and self-employment, as social security systems beyond the pension system may differently affect retirement decisions depending on the country and occupation type (Tables S8-S13 in the Supplementary material). While most interaction terms were not statistically significant, we found heterogeneity in some countries. For example, in England and Poland, retirement appeared to be less beneficial for cognitive function among self-employed individuals compared to non-self-employed individuals.

### Pooling data analysis

We conducted FE and FEIV regressions using cross-country pooling data. In the first stage of FEIV estimation, the Kleibergen–Paap Wald F statistics^31^ exceeded the Stock–Yogo’s critical value for a 10% maximal relative bias,^32^ indicating that our IVs were strongly correlated with retirement. In addition, the over-identification tests^33^ did not reject the null hypothesis that the IVs were uncorrelated with residuals at the 5% significance level (Table S14 in the Supplementary material). Therefore, it is plausible that our IVs satisfy the conditions of a valid IV.

**Figure 2:**
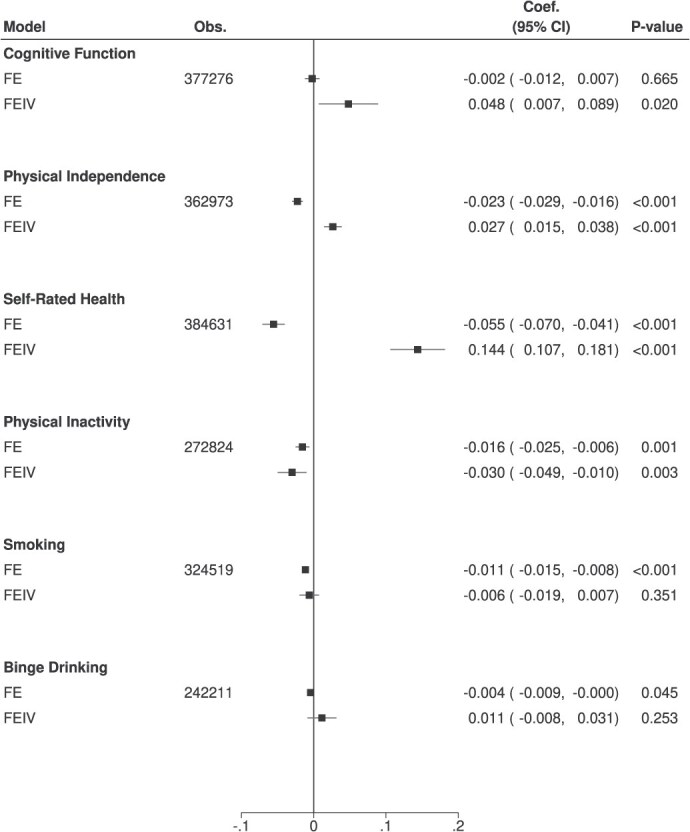
Associations between retirement and outcomes. Abbreviations: obs., observations; Coef., coefficient; FE, fixed effects; FEIV, fixed-effects instrumental variable. Note: All regressions are adjusted for age, age squared, marital status, and fixed effects of individual, year, and interactions between country and year. Robust standard errors clustering at individual, year, and interactions between country and year are calculated.

In FE models without IV, retirement was associated with a lower likelihood of physical independence and a lower score of self-rated health (Figure 2). In contrast, the FEIV models showed that retirement was associated with higher cognitive function (0.048 SD [95% CI, 0.007-0.089]), a greater likelihood of physical independence (2.7%-point [95% CI, 0.015-0.038]), and improved self-rated health (0.144 SD [95% CI, 0.107-0.181]). Consistently, retirement was associated with reduced physical inactivity (−3.0%-point [95% CI, −0.049 to −0.010]) in the FEIV model.

### Individual-level heterogeneity

To assess the individual-level heterogeneity, interaction terms of retirement with demographic and socio-economic factors were included in the FEIV models, such as gender, educational levels, and pre-retirement job characteristics, including physical labor and a job with low control (Tables S15-S18 in the Supplementary material). In summary, notable interactions were not found for most factors, except for gender.

**Figure 3:**
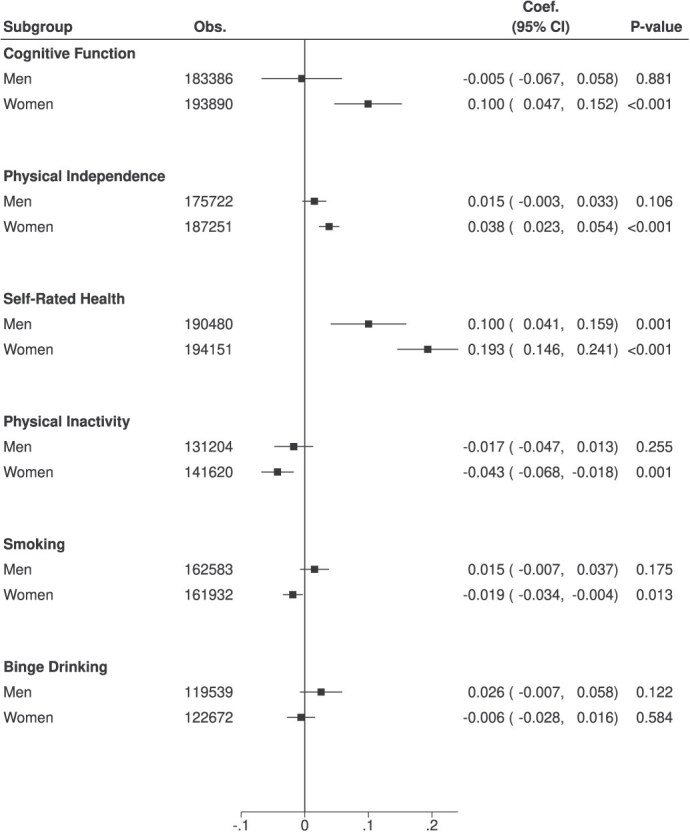
Gender differences in the associations between retirement and outcomes. Abbreviations: obs., observations; Coef., coefficient. Note: All regressions are adjusted for age, age squared, marital status, and fixed effects of individual, year, and interactions between country and year. Robust standard errors clustering at individual, year, and interactions between country and year are calculated.

Given the presence of interactions between retirement and gender, we performed stratified analysis by gender (Figure 3). Among women, retirement was associated with higher cognitive function (0.100 SD [95% CI, 0.047-0.152]) and a greater likelihood of physical independence (3.8%-point [95% CI, 0.023-0.054]). In both genders, retirement was associated with increased self-rated health, but women indicated a larger point estimate than men (women: 0.193 SD [95% CI, 0.146-0.241], men: 0.100 SD [95% CI, 0.041-0.159]). Consistently, retirement was associated with reduced physical inactivity (−4.3%-point [95% CI −0.068 to −0.018]) and smoking (−1.9%-point [95% CI, −0.034 to −0.004]) among women, which was not observed among men.

### Sensitivity analyses

First, we excluded participants who claimed to be retired but were still working or partly retired, thereby considering the alternative definition of retirement, that is, fully retired. Results were similar to the main findings (Figure S12 in the Supplementary material). Second, we tested narrower (52-68 years) and broader (50-80 years) age ranges. In the narrower window, retirement was associated with declined cognitive function among men, while the association between retirement and smoking for women was attenuated towards the null (Figure S13 in the Supplementary material). The analysis using the broader age window yielded larger estimates compared to our main results (Figure S14 in the Supplementary material). Third, we excluded individuals who had been self-employed from the analysis and confirmed that our findings remained robust even after their exclusion (Figure S15 in the Supplementary material). Fourth, we excluded countries with weak IVs^32^: namely, Greece, Latvia, Malta, Portugal, Romania, Costa Rica, Japan, and South Korea. Results were similar to the main findings (Figure S16 in the Supplementary material). Fifth, 24.1% of the participants were from the United States. Additionally, while the current SPA in the United States is gradually increasing from 66 to 67, the SPA for those born in 1937 or earlier was 65, the same as the Medicare eligibility. Thus, we excluded data from the HRS to check the robustness of our findings and obtained consistent results (Figure S17 in the Supplementary material). Sixth, we investigated the short- and long-term impacts of retirement by dividing retirees into those who retired within 5 years and those who retired more than 5 years ago, comparing them with workers. There was a beneficial association between retirement and physical independence among men who retired more than 5 years ago. However, retirement was associated with increased physical inactivity among men who retired within the past 5 years (Figure S18 in the Supplementary material). In stratified analysis by region and retirement timing, we observed similar patterns to the pooled analysis (Figure S19 in the Supplementary material). Seventh, we conducted additional analyses adjusting for time-varying confounders, including household assets and self-reported cardiovascular health status (defined as diagnoses of heart disease and stroke). These analyses yielded similar results to our main findings (Figure S20 in the Supplementary material). Finally, given that the length of a word list in the cognitive function test differed by surveys, we restricted our analysis to surveys with a 10-word list and investigated the association between retirement and the raw scores of cognitive function. The results revealed no clear association among men, while female retirees could recall 0.281 more words than workers, which is in line with the main findings (Table S19 in the Supplementary material).

## Discussion

This study examined the associations of retirement with health and behaviors using harmonized longitudinal data from 35 countries. Our FEIV models revealed that retirement was associated with improved cognitive function and physical independence among women. In both genders, retirement was associated with better self-rated health, but women indicated a larger point estimate than men. Consistently, retirement was associated with decreased physical inactivity and smoking among women, which was not observed among men.

We have hypothesized 3 potential sources of the inconsistent findings in previous studies: differences in statistical methodologies, the country of the study population, and individual characteristics. First, as demonstrated, the associations of retirement flipped to be positive after adopting the IV, suggesting that some studies could not fully address its endogeneity. Indeed, our findings of the beneficial association between retirement and physical function are consistent with recent IV studies.^34^^,^^35^ Second, this study does not support country-level heterogeneity, as we found no signs of interactions in terms of region, country income, and the percentage of the older population. Third, we found substantial gender differences in the associations of retirement with health outcomes, whereas they appeared homogeneous across educational levels and pre-retirement job characteristics. While studies are reporting a disproportionately harmful association between retirement and cognitive function only for men,^22^^,^^24^ our findings highlight its benefits for women. Our findings imply that the average treatment effect of retirement can vary depending on the gender composition within the study population.

In line with the gender differences in health outcomes, we observed the associations of retirement with decreased physical inactivity and smoking only among women. The decreased physical inactivity for women is consistent with a previous study showing that women tend to be more socially active than men after retirement.^22^ Female retirees participate in more social activities and have more channels of social support than men, which can lead to increased opportunities to engage in physical activity.^36^ Additionally, the reduction in smoking among female retirees may be explained by relief from job strain. Smoking is one of the stress-coping behaviors, and women tend to be more reactive to stress compared to men.^37^ Hence, retirement could result in a loss of motivation to smoke for women. Given that unhealthy lifestyles, including physical inactivity and smoking, are potential risk factors for cognitive and physical impairments,^19^^,^^20^ the gender differences in health behaviors may contribute to the heterogeneous associations of retirement. It should be noted that men also benefited from retirement in terms of self-rated health, which aligns with the findings of most previous studies.^4^ The reduction of work and commuting stress, along with the freedom to engage in leisure activities, may have contributed to improved subjective health for both genders.

Our study expands upon the work of Nishimura and colleagues^17^ in several important ways. First, while they examined each country separately, leaving the issue of cross-country variations in the health implications of retirement unsolved, we addressed this by testing effect heterogeneity across countries. We included interaction terms between retirement and country characteristics, such as region, income level, and the percentage of the older population. Our analysis confirmed that the effect of retirement was homogeneous across countries, allowing us to present pooled estimates from data across 35 countries. Moreover, given that many countries began raising their SPA around 2015,^2^ this study incorporates more recent data from a larger number of countries. Finally, while Nishimura et al. focused on health outcomes, our study extends this by also exploring health behaviors, including physical inactivity, smoking, and binge drinking, which enabled us to uncover underlying mechanisms that contribute to the heterogeneous effects on health outcomes.

Although this study contributed to the literature, several limitations should be acknowledged. First, certain discrepancies across surveys were recognized though field experts harmonized data. Although these discrepancies could potentially bias estimations, the inclusion of country FEs could help mitigate some of these biases. Second, measurement errors could occur because most of the measures were self-reported. Nonetheless, the performance of outcome measures has been validated.^8^^,^^15^^,^^18^ Additionally, the straightforward inquiry into retirement status ensured face validity to measure individuals’ recognition, which could induce behavioral adjustments. Third, further studies are needed to determine the mechanism linking retirement to improved health outcomes. Although our study demonstrated gender differences in physical inactivity and smoking after retirement, other factors, such as sleep, diet, and social participation, not provided in the harmonized data, may play a role.

In conclusion, this study underscores that retirement is associated with better health outcomes, especially for women. Policymakers are urged to consider the social benefits of raising the SPA against the social costs related to delayed retirement, such as increased incidence of expensive medical conditions like dementia and functional disability. Additionally, this study highlights the inadvertent role of post-retirement health behaviors in accentuating health disparities. With the global trend of increasing SPA, there is an imperative to promote healthy behaviors post-retirement, with the broader global goal objectives to enhance public health overall.

## Supplementary Material

Web_Material_kwaf126

## Data Availability

The harmonized datasets are available through the Gateway to Global Aging Data website (https://g2aging.org/).
