# Supplementary material for: Heterogeneous associations of retirement with health and behaviors: a longitudinal study in 35 countries
Source: Am J Epidemiol. 2025 Jun 13;195(3):644–52. doi: 10.1093/aje/kwaf126 (PMC13017461; doi:10.1093/aje/kwaf126)
Supplement: Web_Material_kwaf126 [file web_material_kwaf126.zip › 250604_Supplement.pdf]

Web Supplement

Gender differences in the association of retirement with health and behaviors: A longitudinal study in 35 countries

Koryu Sato and Haruko Noguchi

Table S1. Comparison between included and excluded individuals ... 3
Appendix S1. Measurement of outcomes ... 4
Figure S1. Distribution of cognitive function score ... 5
Figure S2. Distribution of physical function score ... 7
Figure S3. Distribution of self-rated health score ... 9
Appendix S2. Measurement of labor force status ... 11
Table S2. Summary of the harmonized variable of labor force status ... 13
Table S3. Early and official retirement age for each country ... 14
Figure S4. Men's retirement rate by country ... 16
Figure S5. Women's retirement rate by country ... 19
Appendix S3. Measurement of potential effect modifiers ... 22
Appendix S4. Model specification ... 22
Table S4. Comparison between participants followed up and those lost to follow-up ... 23
Figure S6. Country-by-country associations between retirement and cognitive function ... 24
Figure S7. Country-by-country associations between retirement and physical independence ... 25
Figure S8. Country-by-country associations between retirement and self-rated health ... 26
Figure S9. Country-by-country associations between retirement and physical inactivity ... 27
Figure S10. Country-by-country associations between retirement and smoking ... 28
Figure S11. Country-by-country associations between retirement and binge drinking ... 29
Table S5. FEIV models with interactions of region ... 30
Table S6. FEIV models with an interaction of country income ... 30
Table S7. FEIV models with an interaction of an aged society ... 31
Table S8. FEIV models for cognitive function with an interaction of self-employment ... 31
Table S9. FEIV models for physical independence with an interaction of self-employment ... 33
Table S10. FEIV models for self-rated health with an interaction of self-employment ... 34
Table S11. FEIV models for physical inactivity with an interaction of self-employment ... 36
Table S12. FEIV models for smoking with an interaction of self-employment ... 37
Table S13. FEIV models for binge drinking with an interaction of self-employment ... 38
Table S14. First stage estimation of FEIV models ... 39
Table S15. FEIV models with an interaction of gender ... 40

Table S16. FEIV models with interactions of educational levels 40
Table S17. FEIV models with an interaction of physical labor 41
Table S18. FEIV models with an interaction of a job with low control 41
Figure S12. FEIV models for the associations of full-retirement with outcomes 42
Figure S13. FEIV models for people aged 52–68 years 43
Figure S14. FEIV models for people aged 50–80 years 44
Figure S15. FEIV models excluding self-employed people 45
Figure S16. FEIV models excluding countries with weak IVs 46
Figure S17. FEIV models excluding data from the United States 47
Figure S18. FEIV models by gender and retirement duration 48
Figure S19. FEIV models by region and retirement duration 49
Figure S20. FEIV models with further adjustments 50
Table S19. FEIV models for the raw scores of cognitive function 51

List of Abbreviations

CHARLS	China Health and Retirement Longitudinal Study
CI	Confidence interval
Coef.	Coefficient
CRELES	Costa Rican Longevity and Healthy Aging Study
ELSA	English Longitudinal Study on Ageing
ERA	Early retirement age
FE	Fixed effect
FEIV	Fixed effects instrumental variable
HRS	Health and Retirement Study
IV	Instrumental variable
JSTAR	Japanese Study of Aging and Retirement
KLoSA	Korean Longitudinal Study of Aging
Obs.	Observations
ORA	Official retirement age
SD	Standard deviation
SHARE	Survey of Health, Ageing and Retirement in Europe
SPA	State pension age
MHAS	Mexican Health and Aging Study

Table S1. Comparison between included and excluded individuals

Country	Included (Workers or Retirees)				Excluded (Never worked)			
	No.	% of men	% of high school graduate	% of college graduate	No.	% of men	% of high school graduate	% of college graduate
Austria	2,877	46.2	53.5	28.1	181	31.5	51.4	24.3
Belgium	4,118	51.8	28.3	39.7	425	31.1	30.4	23.5
Bulgaria	377	43.0	58.9	19.4	78	35.9	47.4	12.8
Croatia	1,119	49.5	30.6	19.7	122	41.8	19.7	11.5
Cyprus	124	42.7	42.7	27.4	29	27.6	41.4	6.9
Czech Republic	3,827	41.4	48.3	13.1	176	48.3	39.8	4.5
Denmark	3,031	48.2	39.3	48.1	210	33.8	43.3	26.2
England	9,895	47.6	52.7	20.8	625	30.4	39.1	10.0
Estonia	3,662	42.3	55.6	27.7	255	53.3	60.4	15.7
Finland	550	47.3	35.5	48.2	63	44.4	38.1	39.7
France	3,540	47.1	41.7	26.7	321	39.6	34.9	21.2
Germany	3,437	49.7	58.4	34.1	330	29.4	67.3	15.2
Greece	2,187	60.5	32.2	30.3	268	28.4	30.6	13.4
Hungary	788	41.1	64.1	18.1	143	38.5	60.8	4.2
Israel	1,447	46.9	34.8	39.7	179	33.5	37.4	19.0
Italy	3,026	56.1	29.7	11.9	297	29.0	20.9	4.7
Latvia	303	41.6	63.4	29.4	70	38.6	71.4	18.6
Lithuania	528	37.1	50.8	45.8	132	41.7	66.7	27.3
Luxembourg	841	54.5	41.5	23.4	83	32.5	33.7	9.6
Malta	239	72.8	58.2	10.9	50	18.0	58.0	2.0
Netherlands	1,862	55.6	28.6	35.3	438	28.3	23.5	19.9
Poland	1,700	41.4	65.4	12.7	310	48.7	69.0	8.1
Portugal	761	50.5	12.6	12.5	187	34.2	8.6	10.2
Romania	560	45.5	59.5	7.5	109	50.5	47.7	3.7
Slovakia	665	47.7	84.4	10.8	55	43.6	87.3	5.5
Slovenia	2,531	44.6	55.6	19.8	129	45.7	56.6	5.4
Spain	2,731	59.6	16.6	16.4	557	31.8	12.4	9.9
Sweden	3,151	45.1	34.6	34.3	140	40.0	42.1	18.6
Switzerland	2,178	48.8	65.6	18.4	158	24.1	56.3	10.8
Costa Rica	1,244	76.9	9.1	12.9	577	34.8	10.6	10.6
Mexico	8,148	66.7	4.7	16.4	2,801	36.6	3.1	6.9
United States	25,753	46.9	58.5	22.6	1,613	32.9	55.6	11.0
China	2,819	54.1	28.3	6.6	119	51.3	20.2	7.6
Japan	1,775	64.8	54.5	22.4	263	37.3	56.3	15.2
South Korea	5,133	53.1	39.9	13.1	642	21.7	32.6	9.3
Total	106,927	50.5	44.1	23.0	12,135	34.7	31.8	11.6

Note: Educational attainment was classified into three groups using the 1997 International Standard Classification of Education codes—less than upper secondary education, upper secondary and vocational training (high school graduate), and tertiary education (college graduate).

Appendix S1. Measurement of outcomes

1. Cognitive function

As a measure of cognitive function, we focused on episodic memory, a neurocognitive system responsible for recollecting past experiences. Following the Consortium to Establish a Registry for Alzheimer's Disease battery,[1] a list of common words was verbally presented to the participants, followed by an immediate recollection of as many words as possible. After approximately 5 min, participants were asked to recall the words once again. The episodic memory score was calculated by adding the number of words remembered during both the immediate and delayed recalls. Typically, most surveys included a list of ten words, providing a score range from 0 to 20. Waves 1 and 2 of the HRS comprised 20 words on the list, while the MHAS consisted of 8 words, and the CRELES and the KLoSA contained 3 words. To enable comparison, a z-score (i.e., mean of 0 and standard deviation of 1) was computed for each country by pooling all the surveys in the country (except for the HRS; we separately computed z-scores for waves 1, 2, and 3–14 of the HRS).

2. Physical inactivity

We considered those who engaged in vigorous or moderate physical activity (PA) less than once per week to be physically inactive. We constructed a binary variable from two original variables of the frequency of vigorous and moderate PAs. However, for South Korea, we used only the variable of vigorous PA because the KLoSA did not ask about the frequency of moderate PA. In wave 7 of SHARE, only those who participated in wave 3 were asked about the frequency of PA; thus, all observations from Bulgaria, Cyprus, Finland, Latvia, Lithuania, Malta, Romania, and Slovakia were excluded from the analysis because individuals had only one observation. In waves 1–3 of CHARLS, only half of the participants were asked questions. We also excluded some observations due to the incompatibility of questions; MHAS, waves 1–2 of CRELES, and waves 1–6 of HRS asked whether the participant engaged in vigorous PA three times or more per week; and JSTAR asked for minutes of exercise on weekdays and weekends.

3. Smoking status

Smoking status indicates whether the participant is currently smoking. In Wave 6 of SHARE, those who had been interviewed previously were not asked about their smoking status. In wave 7, only new participants and those who were in wave 3 and had previously reported smoking were asked about their current smoking status. Thus, all the observations from Bulgaria, Cyprus, Finland, Latvia, Lithuania, Malta, Romania, and Slovakia were excluded due to a single observation.

4. Binge drinking

We defined binge drinking as consuming five or more drinks per day for men and four or more for women.[2] Waves 1 and 6–8 of SHARE, wave 1 of ELSA, waves 1–2 of HRS, and all waves of CRELES and CHARLS did not provide the number of drinks per day. Thus, all the observations from Bulgaria, Croatia, Cyprus, Finland, Greece, Hungary, Latvia, Lithuania, Luxembourg, Malta, Portugal, Romania, and Slovakia were excluded from the analysis because individuals had only one observation. In some study sites of JSTAR, the question was not asked in wave 2.

---

[1] Morris JC, Heyman A, Mohs RC, et al. The Consortium to Establish a Registry for Alzheimer's Disease (CERAD). Part I. Clinical and neuropsychological assessment of Alzheimer's disease. Neurology. 1989;39(9):1159-1165. doi:10.1212/wnl.39.9.1159

[2] Centers for Disease Control and Prevention. Binge Drinking [Internet]. Cent. Dis. Control Prev. 2022 [cited 2022 Jun 16]. Available from: https://www.cdc.gov/alcohol/fact-sheets/binge-drinking.htm

Figure S1. Distribution of cognitive function score

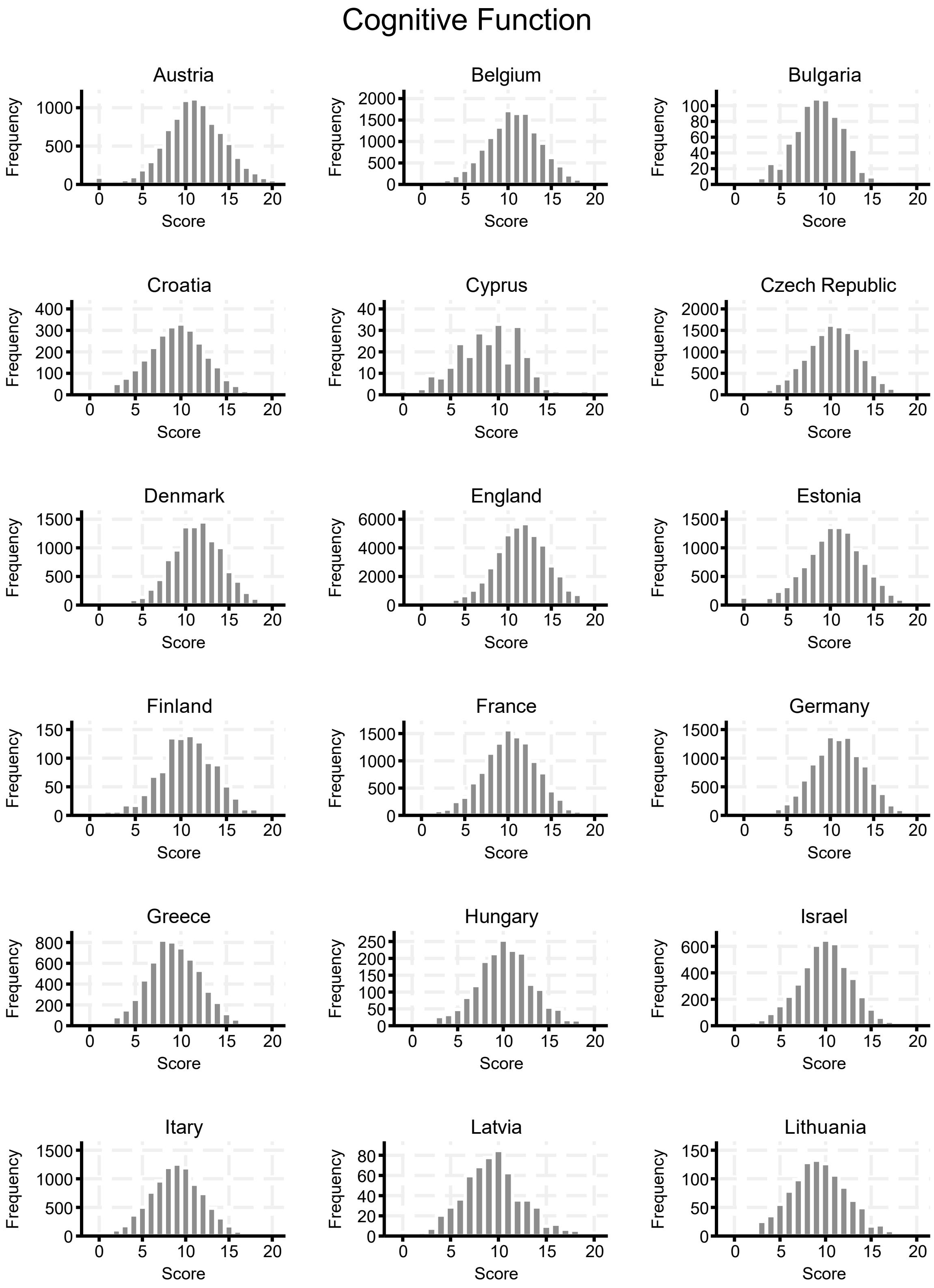

Cognitive Function (cont.)

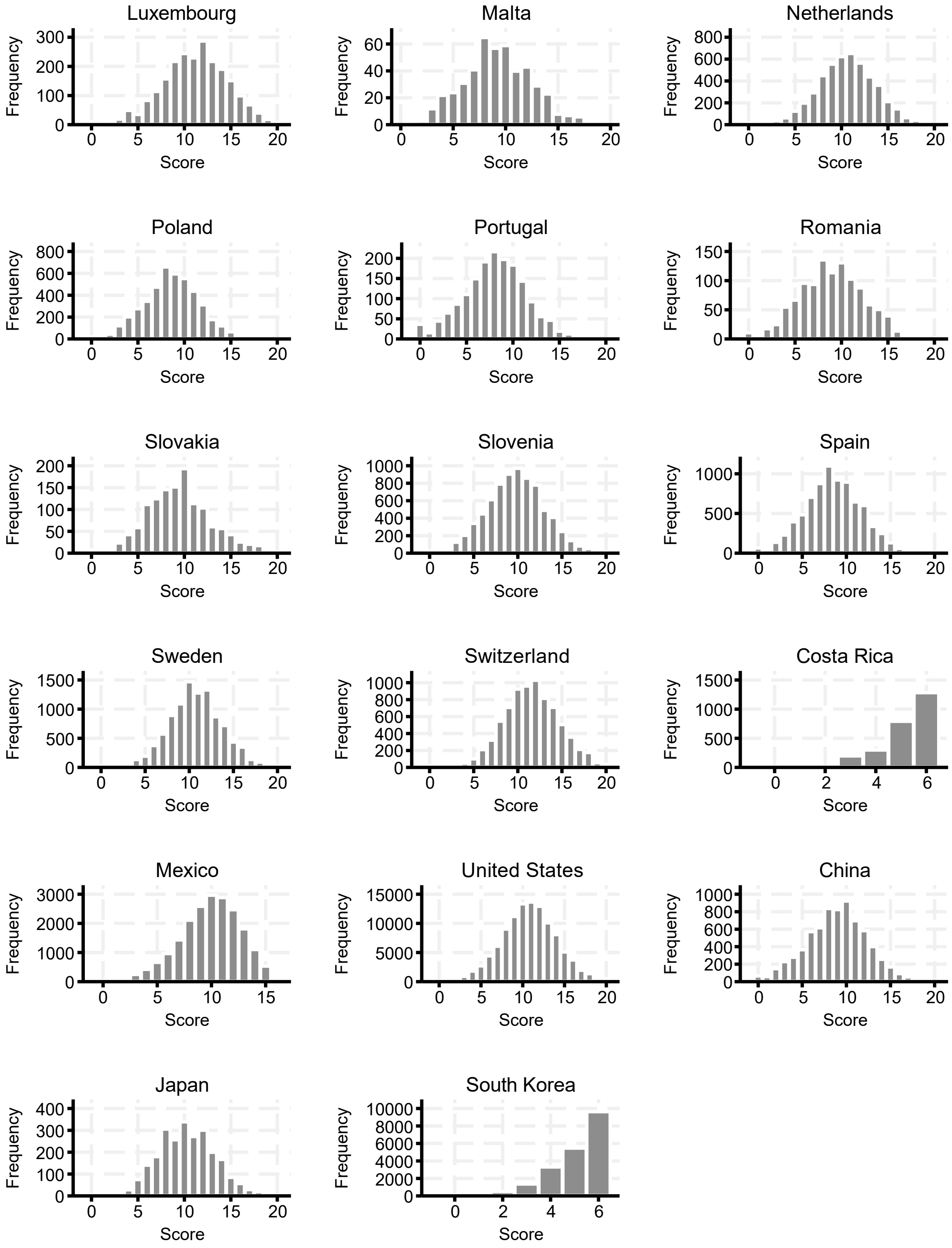

Figure S2. Distribution of physical function score

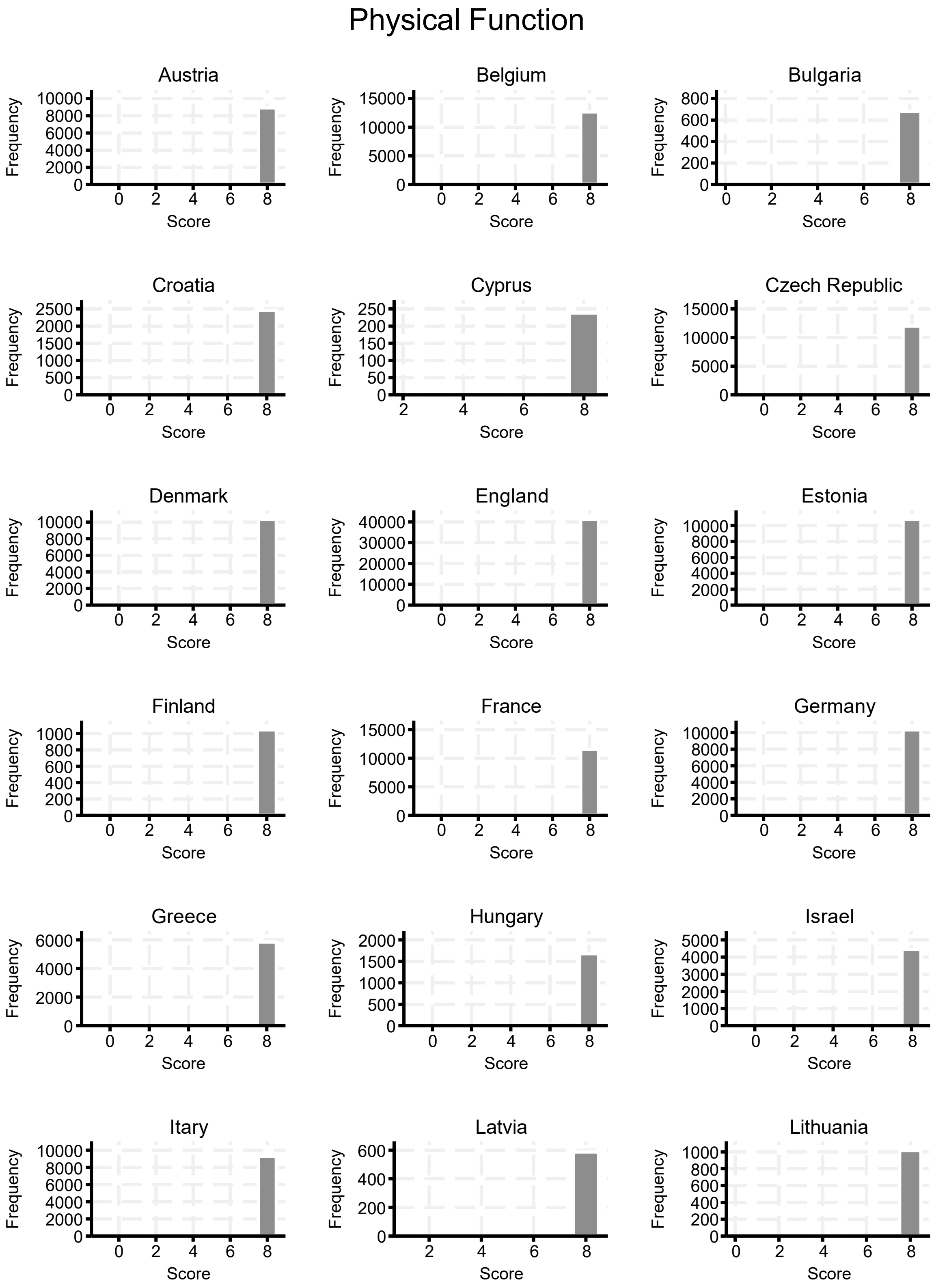

Physical Function (cont.)

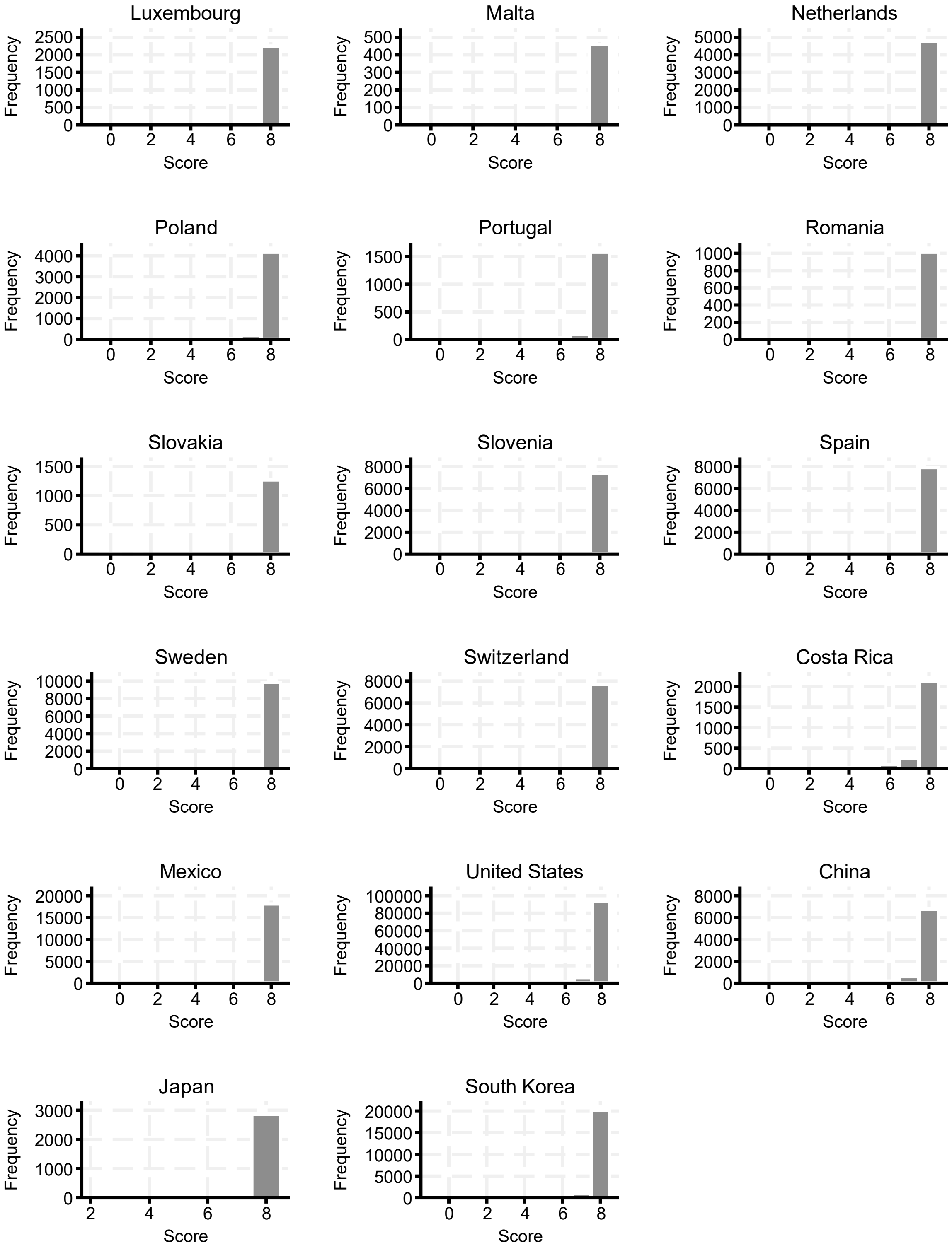

Figure S3. Distribution of self-rated health score

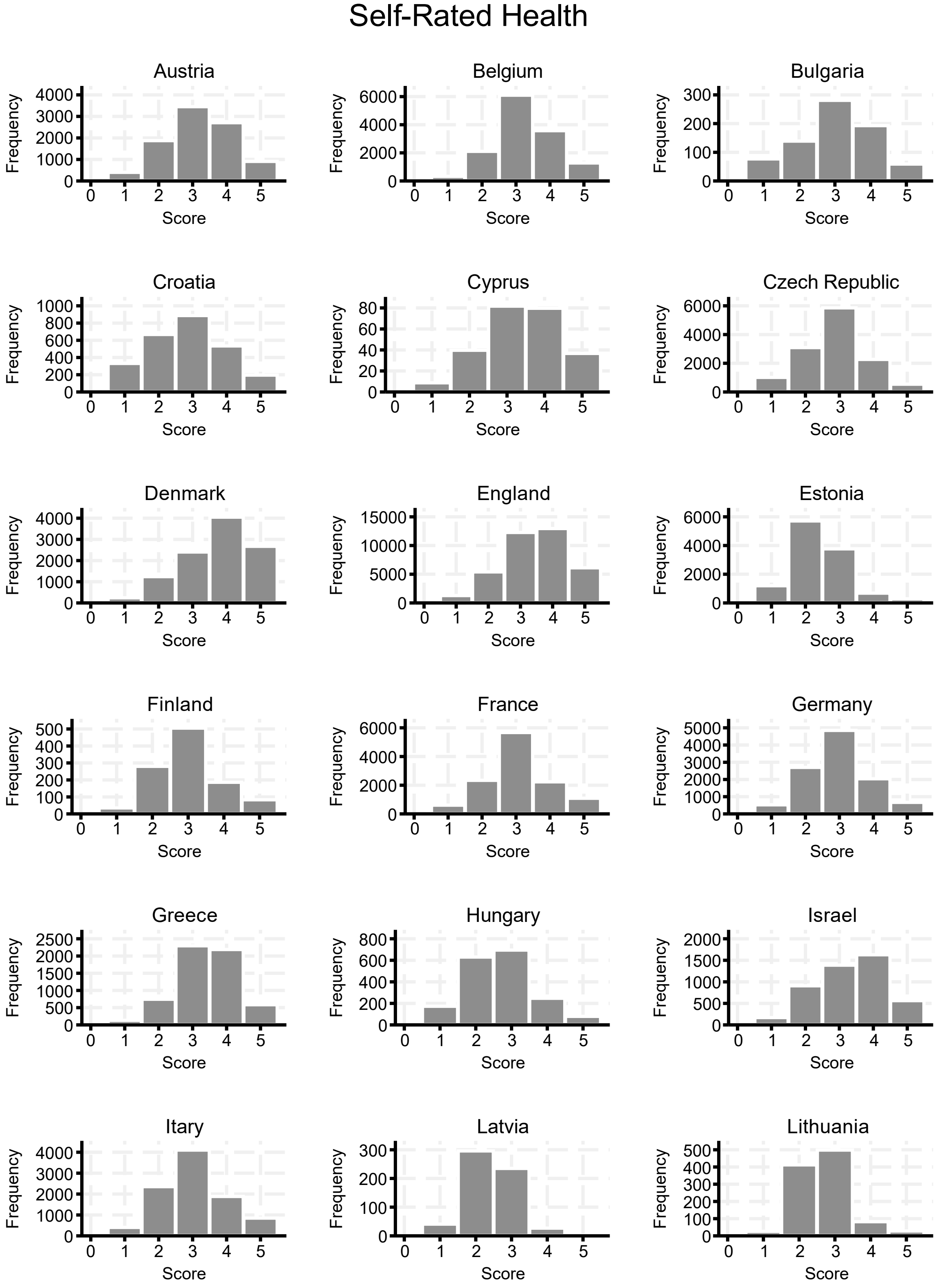

Self-Rated Health (cont.)

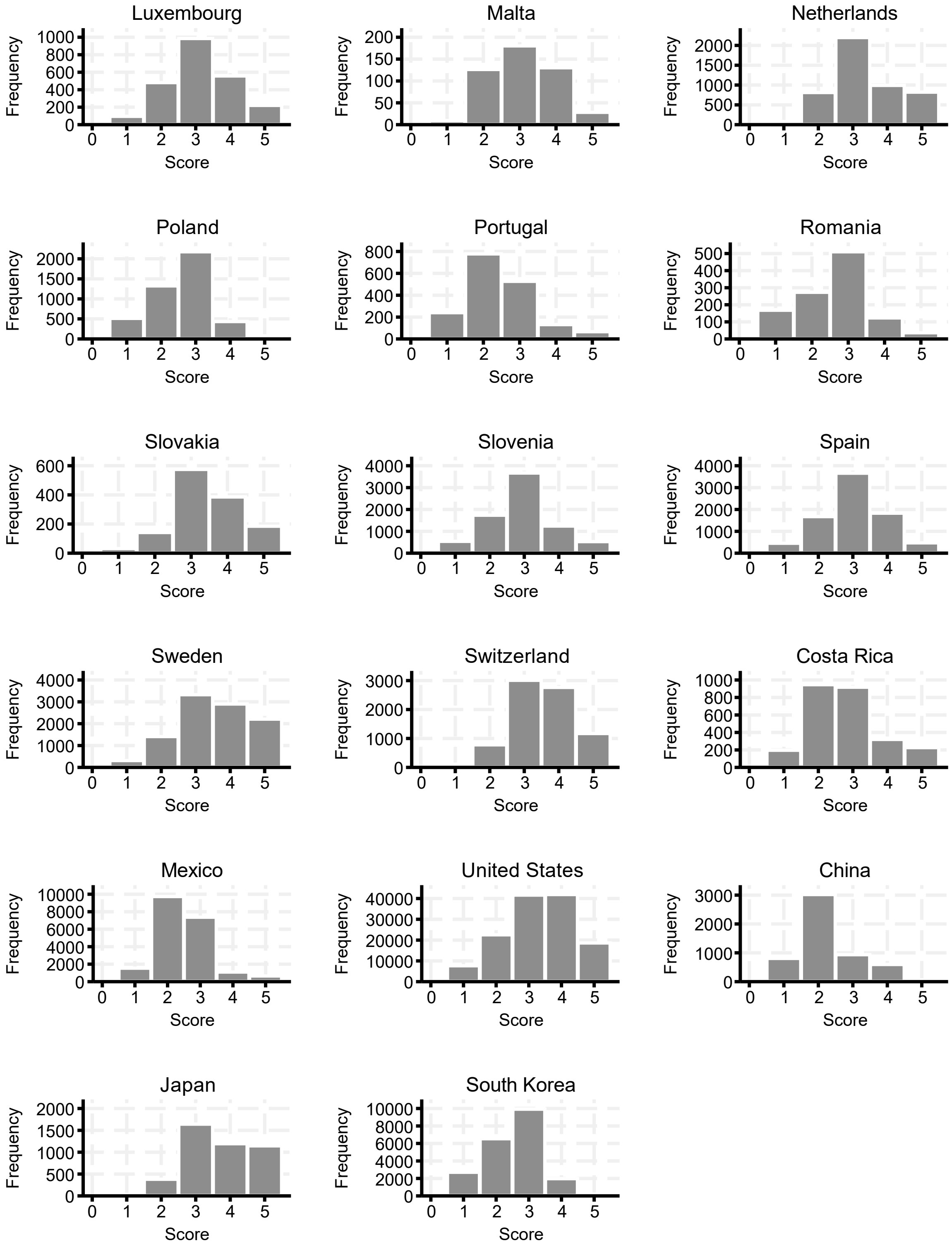

Appendix S2. Measurement of labor force status

1. SHARE

SHARE asks participants, "In general, how would you describe your current situation?" They then choose the best description of their current labor force status from a list of options: 1) retired, 2) employed or self-employed (including working for a family business), 3) unemployed and looking for work, 4) permanently sick or disabled, 5) homemaker, or 6) other (renter, living off own property, student, or doing voluntary work). The harmonized variable was constructed based on responses to this direct question.

2. ELSA

ELSA asks participants, "Which of these, would you say, best describes your situation?" They then choose the best description of their current labor force status from a list of options: 1) employed, 2) self-employed, 3) unemployed, 4) partly retired, 5) retired, 6) permanently sick or disabled, or 7) looking after home or family. The harmonized variable was constructed based on responses to this direct question.

3. CRELES

CRELES asks participants whether they have ever had a job for which they received payment in money or kind. If the respondent answered yes, they were asked what they did during most of the last week: worked, worked with a family business, did not work but had a job, looking for work, did household chores, or did not work. Participants who were not working were then asked if they had not worked: less than 2 years, more than 2 years, or had never worked.

If the participant answered that they worked, helped with a family business, or did not work last week but had a job, the harmonized variable was set to "working." If the participant reported that they were looking for work, it was set to "unemployed." If the participant reported that they were doing household chores, it was set to "doing household chores." If the participant reported that they had worked in the past but were not currently working, it was set to "retired." If the participant reported that they had never worked, it was set to "never worked."

4. MHAS

MHAS asks participants whether they have worked or are currently working. In waves 2–4, it also asks the main reason why they were not currently working: dedicated to household chores, retired, old age, sick or temporarily disabled, unable to work for the rest of life, and does not have customers or cannot find work.

If the participant reported that they were currently working, the harmonized variable was set to "working." If the participant is currently looking for work or does not work but "does not have customers or cannot find work," it is set to "unemployed." If the participant mentioned retirement, regardless of current work, it was set to "retired." If the participant is "sick or temporarily disabled" or "unable to work for rest of life," it is set to "disabled." Otherwise, the variable is set to "not in the labor force." The question asking the reason for not working is not included in wave 1; thus, the harmonized variable has an integrated category indicating "unemployed, retired, or disabled" in wave 1. If the present study, we treated those in the integrated category in wave 1 as retirees only if they were categorized as "retired" in wave 2; otherwise, they were not included in the analyses.

5. HRS

Participants in HRS provided information on their labor force status at several time points in an interview. First, HRS asks the participants to select all applicable options from a list that includes 1) working now, 2) unemployed and looking for work, 3) temporarily laid off, on sick or other leave, 4) disabled, 5) retired, 6) homemaker, or 7) other (specify). It also asks them whether they are currently working for payment, the usual number of hours per week if applicable, and whether they consider themselves partly retired, completely retired, or not retired.

If the participant reports working full-time (i.e., working 35+ hours per week or 36+ weeks per year), the harmonized variable is set to "working full-time." If the participant is working part-time and does not mention retirement, it is set to "working part-time." If the participant is working part-time and mentions retirement, it is set to "partly retired." If the participant is not working but is looking for a job, it is set to "unemployed." If the participant is not looking for a job and there is any mention of retirement, it is set to "retired." If retirement is not mentioned and disabled employment status is given, it is set to "disabled." Otherwise, the variable is set to "not in the labor force."

6. CHARLS
CHARLS asks participants whether they engaged in agricultural work for more than 10 days in the past year, worked for at least 1 hour last week if not engaged in agricultural work, were temporarily laid-off or on sick or other leave, worked for at least a few months, whether they were homemakers, completed retirement procedures, and currently retired (including early retirement or internal retirement).

If the participants report working for other farmers, the harmonized variable is set to "agricultural employed." If the participants reported working for their household, it is set to "agricultural self-employed." If the participants describe their non-agricultural job as employment, it is set to "non-agricultural employed." If the participants describe their non-agricultural job as self-employment, it is set to "non-agricultural self-employed." If the participants describe their non-agricultural job as an unpaid family business, it is set to "non-agricultural unpaid family business." If the participants report not currently working but had worked for at least 3 months and have searched for a job in the past month, it is set to "unemployed." If the participants declare to have completed retirement procedures or describe themselves as retired, it is set to "retired." If the participants reported never worked, it was set to "never worked."

7. JSTAR
JSTAR asks participants whether they are currently employed, looking for a job, or intend to look for work in the future. If they were neither a worker nor a job seeker, they were asked about their current status with the following response options: 1) retired, 2) keep house, 3) receive medical care, 4) other, 5) do not know, and 6) refused to answer.

If the participant reports working full-time or working 35+ hours per week and 36+ weeks per year, the harmonized variable is set to "working full-time." If the participant reports working part-time or less than 35 hours per week or 36 weeks per year, it is set to "working part-time." If the participant reports currently working as an owner of an independent business or having a side job at home, it is set to "self-employed."

If the participant reports not working but is looking for a job and there is no mention of retirement, it is set to "unemployed." If the participant reports looking for a part-time job and mentions retirement, it is set to "partly retired." If the participant is not working and not looking for work, and there is any mention of retirement, it is set to "retired." If retirement is not mentioned and disabled employment status is given, it is set to "disabled." If neither retirement nor disability is mentioned, but a homemaker situation is given, it is set to "not in the labor force."

8. KLoSA
KLoSA asks participants whether they are currently working or looking for a job. If they are neither a worker nor a job seeker, they are asked about their retirement status with the following response options: 1) worked before but currently retired, 2) worked before and intended to work in the future but currently not looking for a job, and 3) never had a job before.

If the participant is employed by another person or company for payment, the harmonized variable is set to "employed full-time" or "employed part-time," based on the working classification the participant gave for the job. If the participant report being self-employed, it is set to "self-employed." If the participant is employed and reports working without payment for family more than 18 hours per week, it is set to "help with family 18 hours or more per week." If a non-working participant is looking for work and reports being able to work if offered a job and then confirms that they have done something to find work in the last 4 weeks, it is set to "unemployed." If a non-working participant is not looking for work and reports being retired, it is set to "retired." If the participant reports being retired but later mentions working for payment or looking for paid work, it is set to "partly retired." If a non-working participant is looking for work but then reports not being able to accept work or looking for work due to poor health or a disability, it is set to "disabled." Otherwise, it is set to "not in labor force."

Table S2. Summary of the harmonized variable of labor force status

This study	SHARE	ELSA	CRELES	MHAS	HRS	CHARLS (urban residents only)	JSTAR	KloSA
Included as those "working"	1. employed or self employed	1. employed<br>2. self-employed	1. working	1. working	1. working full-time<br>2. working part-time	1. agricultural employed<br>2. agricultural self-employed<br>3. non-agricultural employed<br>4. non-agricultural self-employed<br>5. non-agricultural unpaid family business	1. working full-time<br>2. working part-time<br>8. self-employed	1. working full-time<br>2. working part-time<br>3. self-employed<br>4. help with family 18 hours or more per week
Included as those being "retired"	5. retired	4. partly retired<br>5. retired	4. retired	3. retired	4. partly retired<br>5. retired	7. retired	4. partly retired<br>5. retired	6. partly retired<br>7. retired
Excluded from analyses	3. unemployed<br>6. permanently sick or disabled<br>8. homemaker	3. unemployed<br>6. disabled<br>7. looking after home or family	2. unemployed<br>3. doing household chores<br>5. never worked	2. unemployed<br>4. disabled<br>5. not in labor force	3. unemployed<br>6. disabled<br>7. not in labor force	6. unemployed<br>8. never worked	3. unemployed<br>6. disabled<br>7. not in labor force	5. unemployed<br>8. disabled<br>9. not in labor force

Note: Retirement status was determined based on the harmonized variable of labor force status (RwLBRF).

Table S3. Early and official retirement age for each country

Country	Year	Men		Women	
		ERA	ORA	ERA	ORA
Austria[a]	2018	NA	65	NA	60
Belgium[b]	2018	63	65	63	65
Bulgaria[c]	2018	63.08	64.08	60.17	61.17
Croatia[d]	2018	60	65	57	62
Cyprus	2018	63	65	63	65
Czech Republic[e]	2018	60	63.16	59.66	62.66
Denmark[f]	2018	NA	65	NA	65
England[g]	2018	NA	65	NA	65
Estonia[h]	2018	60.5	63.5	60.5	63.5
Finland	2018	63	65	63	65
France[i]	2018	62	67	62	67
Germany[j]	2018	63	65.58	63	65.58
Greece[k]	2018	62	67	62	67
Hungary[l]	2018	NA	63.5	NA	63.5
Israel[m]	2018	NA	67	NA	62
Italy[n]	2018	62	66.58	62	66.58
Latvia[o]	2018	61.25	63.25	61.25	63.25
Lithuania[p]	2018	58.67	63.67	57.33	62.33
Luxembourg	2018	57	65	57	65
Malta[q]	2018	61	62	61	62
Netherlands[r]	2018	NA	66	NA	66
Poland[s]	2018	NA	65	NA	60
Portugal[t]	2018	60	66.33	60	66.33
Romania[u]	2018	60	65	55.92	60.92
Slovakia[v]	2018	60.42	62.42	60.42	62.42
Slovenia[w]	2018	60	65	59.67	64
Spain[x]	2018	61.5	65.5	61.5	65.5
Sweden[y]	2018	61	65	61	65
Switzerland	2018	63	65	62	64
Costa Rica	2013	62	65	60	65
Mexico	2018	60	65	60	65
United States[z]	2018	62	66	62	66
China[aa]	2016	NA	60	NA	50
Japan	2012	60	65	60	65
South Korea[ab]	2018	57	61	57	61

Source: The United States Social Security Administration "Social Security Programs Throughout the World"; Organisation for Economic Co-operation and Development "Pensions at a Glance"; websites of the authorities of each country.
Note: ERA, ORA, and NA denote early retirement age, official retirement age, and not applicable, respectively.

[a] ERA was 61.5 for men and 56.5 for women in 2004 and gradually increased to be phased out in 2017.
[b] ERA gradually increased from age 60 to 63 from 2013 to 2018.
[c] ORA is gradually increasing from age 63 to 65 by 2029 for men and from age 60 to 65 by 2037 for women. Early retirement is possible up to one year prior to the ORA.
[d] ERA and ORA for women are gradually increasing from age 55 to 60 and 60 to 65 by 2030, respectively.
[e] ORA is gradually increasing from age 60 to 65 for men and 57 to 65 for women without children by 2030.
[f] ORA was 67 for those who reached age 60 before 1 July 1999. ORA is gradually increasing to age 67 from 2019 to 2022.
[g] ORA for women gradually increased from age 60 to 65 from 2010 to 2018.
[h] ORA is gradually increasing from age 63 to 65 from 2017 to 2026. Early retirement is possible up to three years prior to the ORA.
[i] ERA is increasing from age 60 to 62, and ORA from 65 to 67, depending on the year of birth.
[j] ERA and ORA are gradually increasing from age 63 to 65 and 65 to 67 by 2029, respectively.
[k] ERA increased from age 60 to 62 for men and 55 to 62 for women, and ORA from 65 to 67 for men and 60 to 67 for women in 2013.
[l] ORA is gradually increasing from age 62 to 65 by 2022.
[m] ORA is increasing from age 65 to 67 for men and 60 to 62 for women, depending on the year of birth.
[n] ERA gradually increased from age 57 to 62, and ORA from 65 to 67 for men and 60 to 67 for women in 2019.
[o] ORA is gradually increasing from age 62 to 65 from 2013 to 2025. Early retirement is possible up to two years prior to the ORA.
[p] ORA is gradually increasing to age 65 by 2026. Early retirement is possible up to five years prior to the ORA.
[q] ORA is gradually increasing to age 65, depending on the year of birth.
[r] ORA is gradually increasing to age 67 by 2024, depending on the year of birth.
[s] ORA increased from age 65 to 65.58 for men and 60 to 60.58 for women from 2012 to 2015 but returned to age 65 and 60 in 2017.
[t] ERA increased from age 55 to 60 in 2015, and ORA is gradually increasing to age 66.5 by 2021.
[u] ORA for women is gradually increasing to age 63 by 2030. Early retirement is possible up to five years prior to the ORA.
[v] ORA is gradually increasing from age 62 based on increases in life expectancy from 2016. Early retirement is possible up to two years prior to the ORA.
[w] ERA (with 40 years of contribution) gradually increased from age 58 to 60 in 2018 for men and 2019 for women. ORA (with 20 years of contribution) gradually increased from age 63 to 65 from 2012 to 2016 for men and 61 to 65 from 2012 to 2020 for women.
[x] ORA is gradually increasing from age 65 to 67 from 2012 to 2027. Early retirement is possible up to four years prior to the ORA in the case of involuntary unemployment.
[y] The earning-related national pension and guarantee pension benefits are available from ages 61 and 65, respectively.
[z] ORA gradually increased from age 65 to 66, depending on the year of birth.
[aa] ORA of 50 is for non-professional salaried women.
[ab] ERA is gradually increasing from age 55 to 60 from 2012 to 2029, and ORA from 60 to 65 from 2012 to 2034.

Figure S4. Men's retirement rate by country

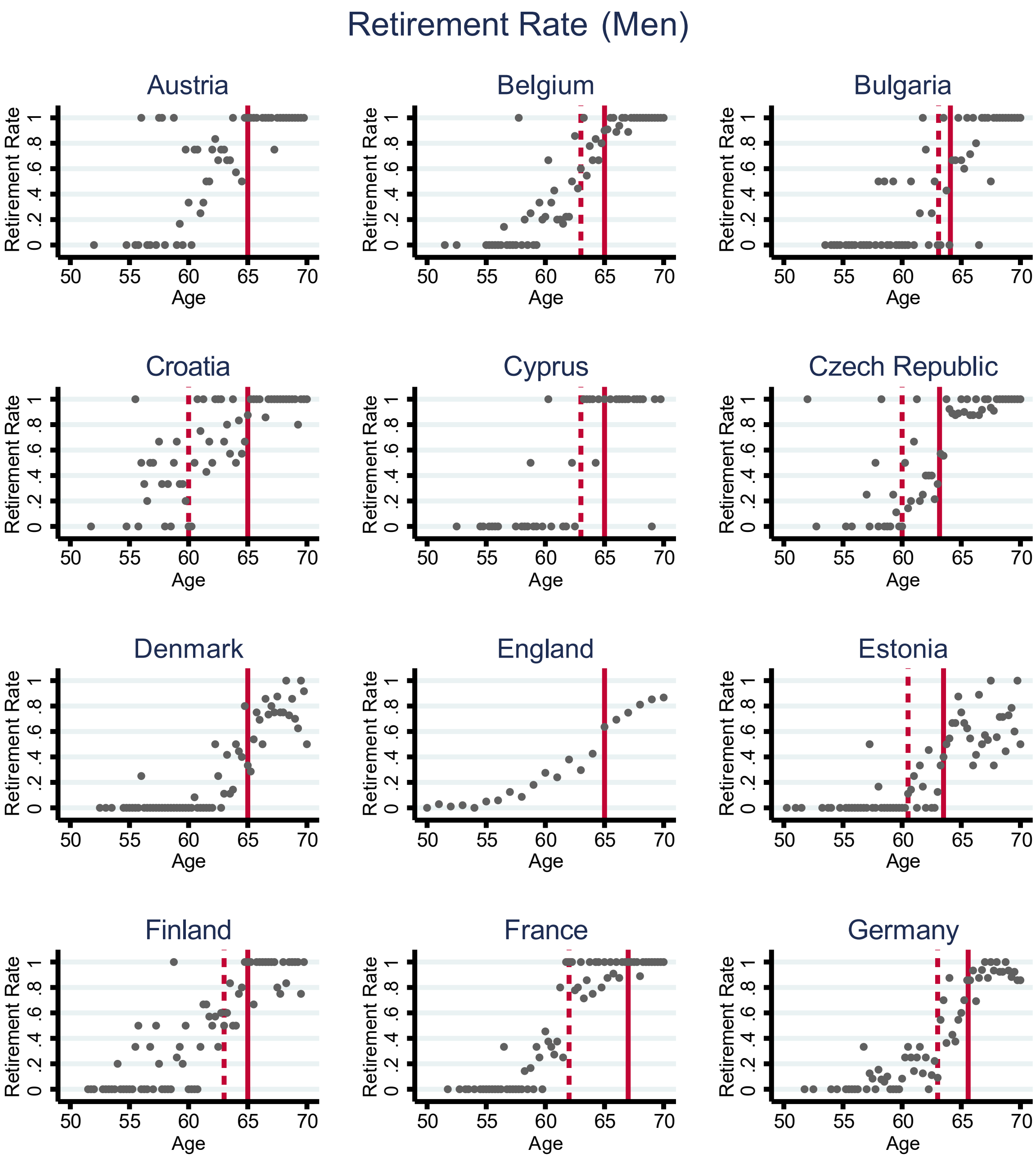

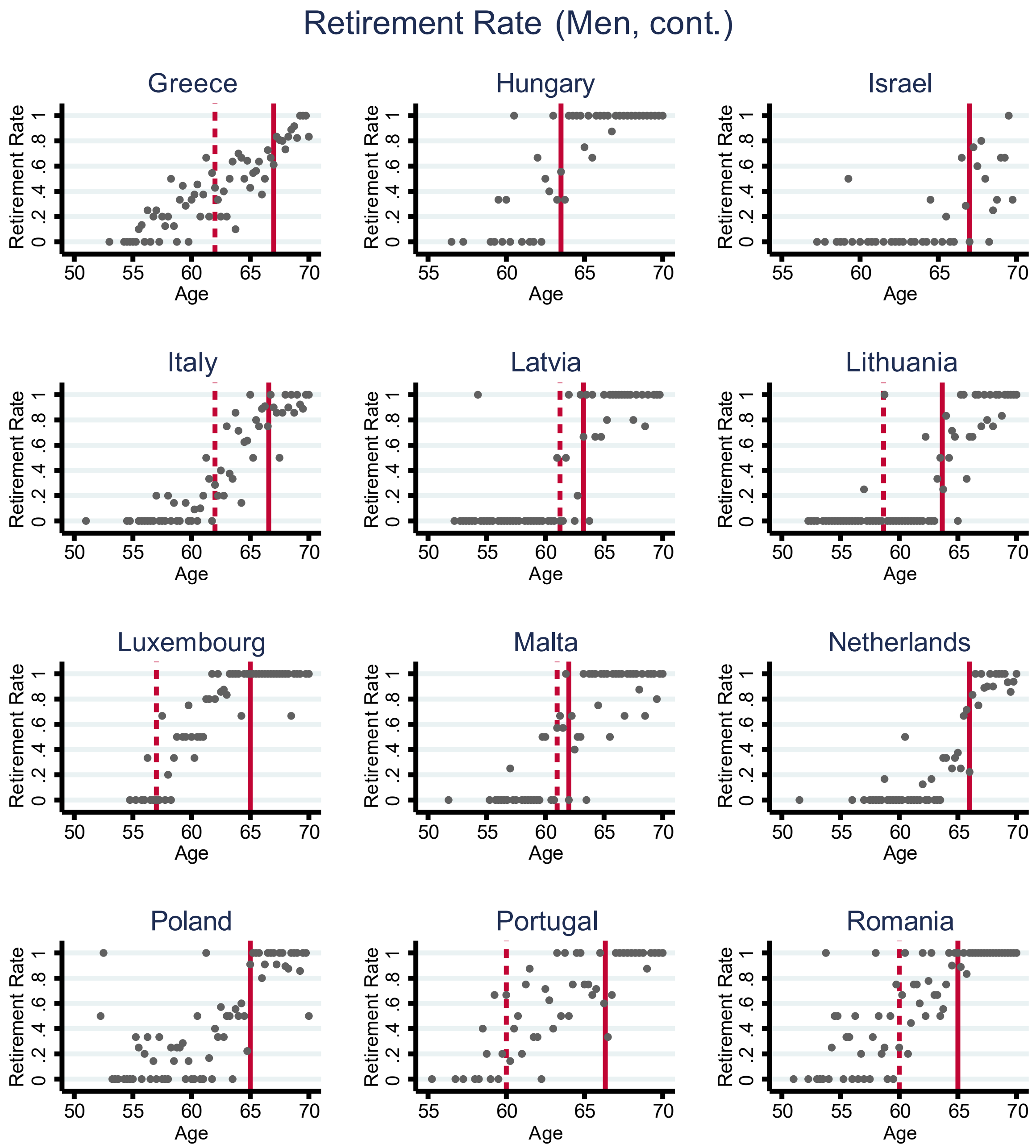

Retirement Rate (Men, cont.)
Greece
Hungary
Israel
Italy
Latvia
Lithuania
Luxembourg
Malta
Netherlands
Poland
Portugal
Romania
Retirement Rate
Age

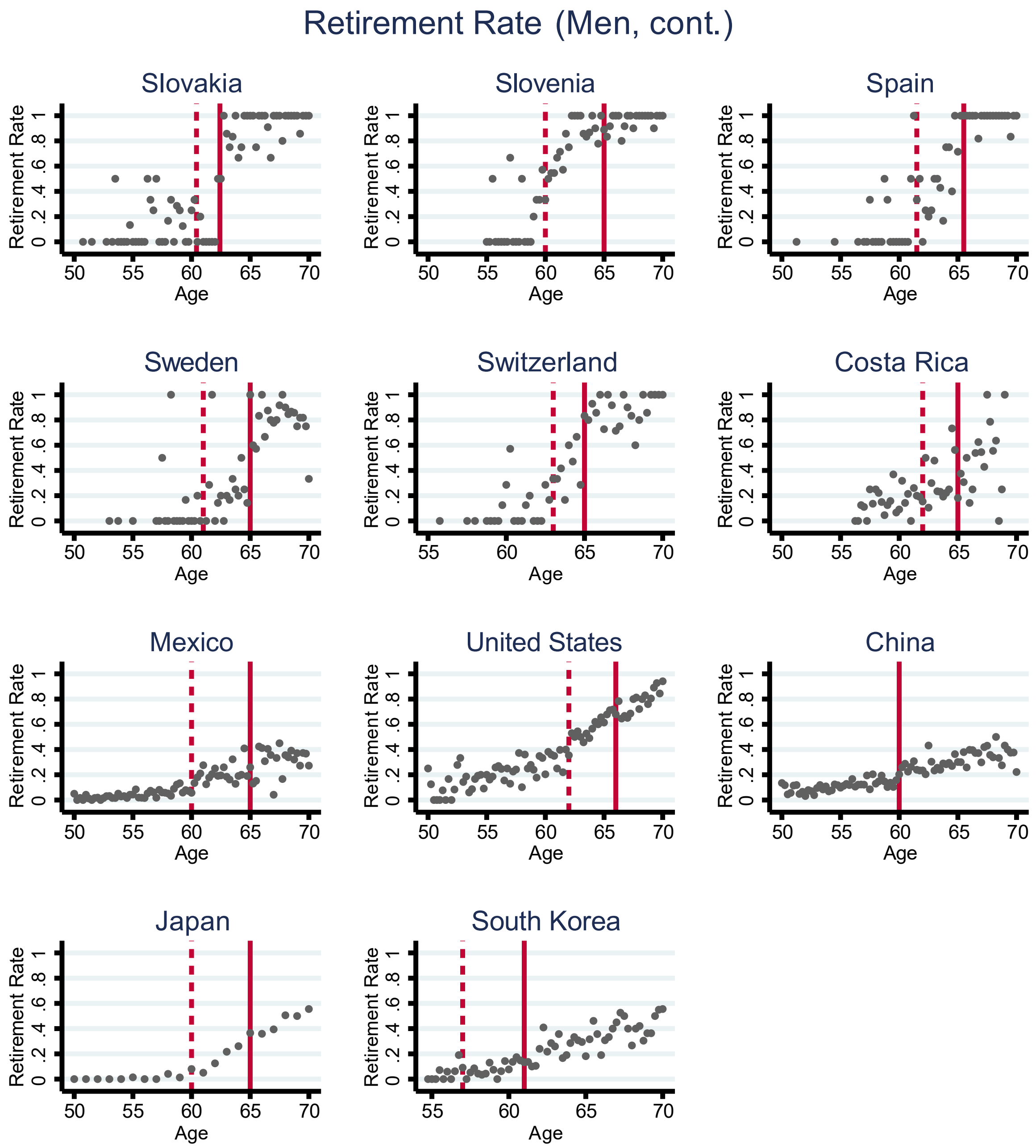

Note: The latest surveys were utilized to illustrate the figures. Each individual dot represents the average retirement rate for each 3-month intervals, as monthly age data was unavailable in England and Japan. The retirement rate is calculated by dividing the number of retirees by the sum of retirees and non-retired individuals who are not working due to reasons other than retirement (e.g., unemployed, disabled, or homemaker), with the exclusion of the latter. The dashed red line denotes the ERA, while the solid red line represents the ORA that corresponds to the survey year.

Figure S5. Women's retirement rate by country

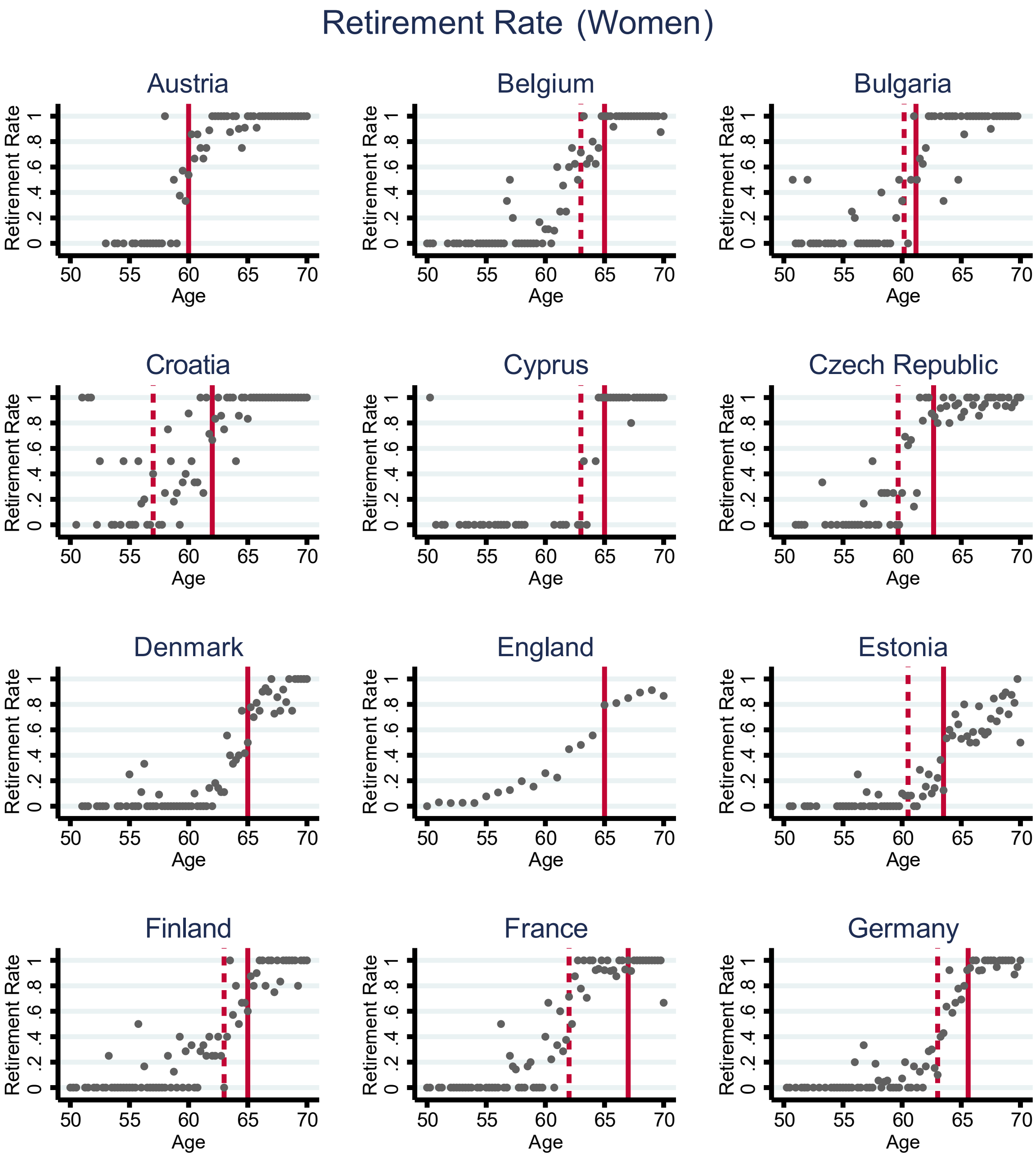

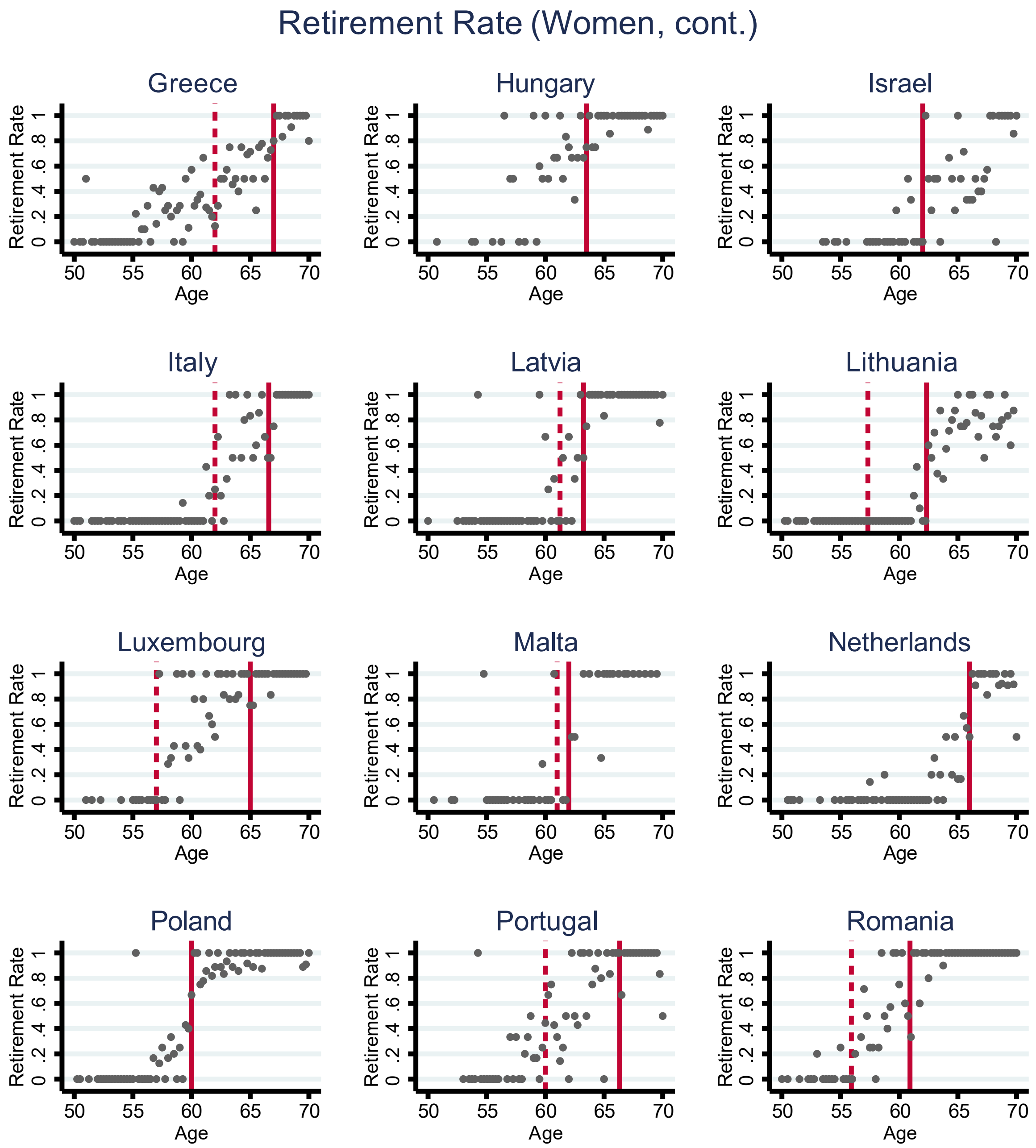
Retirement Rate (Women, cont.)
Greece
Hungary
Israel
Italy
Latvia
Lithuania
Luxembourg
Malta
Netherlands
Poland
Portugal
Romania
Retirement Rate
0 .2 .4 .6 .8 1
50 55 60 65 70
Age

Retirement Rate (Women, cont.)

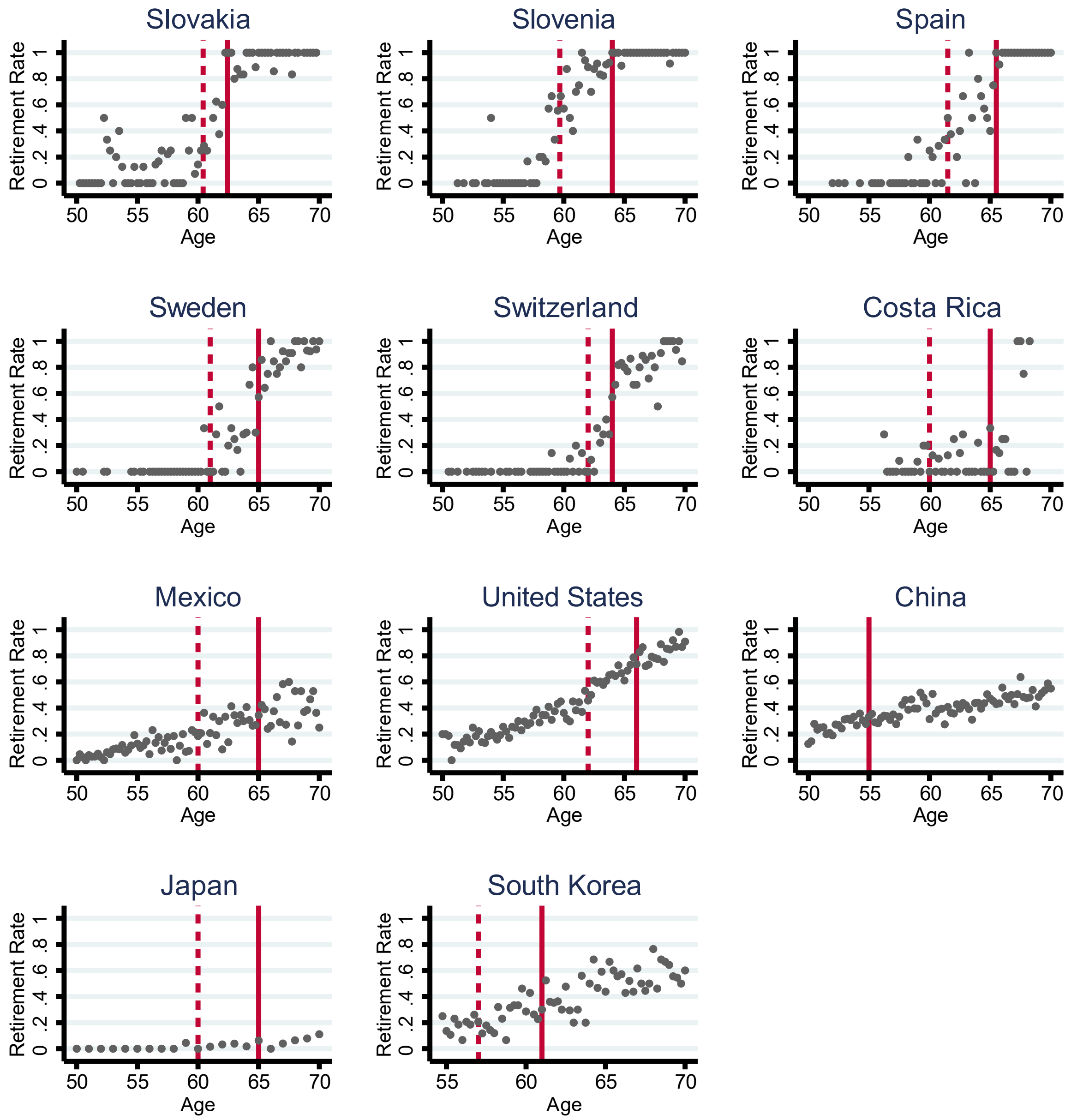

Note: The latest surveys were utilized to illustrate the figures. Each individual dot represents the average retirement rate for each 3-month intervals, as monthly age data was unavailable in England and Japan. The retirement rate is calculated by dividing the number of retirees by the sum of retirees and non-retired individuals who are not working due to reasons other than retirement (e.g., unemployed, disabled, or homemaker), with the exclusion of the latter. The dashed red line denotes the ERA, while the solid red line represents the ORA that corresponds to the survey year.

Appendix S3. Measurement of potential effect modifiers

1. Country characteristics

We performed interaction tests across country characteristics, grouping regions into Europe (including Israel), America (Costa Rica, Mexico, and the United States), and Asia (China, Japan, and South Korea); classifying countries as high-income (all European countries, United States, Japan, and Korea) or low-middle income (Bulgaria, Romania, Costa Rica, Mexico, and China) based on Gross National Income per capita as defined by the World Bank[3]; and considering a country to be an aged society if the percentage of the population aged 65 years and older exceeded 14%.[4]

2. Educational attainment

Educational attainment was classified into three groups using the 1997 International Standard Classification of Education codes—less than upper secondary education as low, upper secondary and vocational training as middle, and tertiary education as high.

3. Pre-retirement job characteristics

During the surveys, participants who were currently employed were asked to rate their agreement with the following statements regarding the physical demands[5] and control of their job[6]; "My job is physically demanding" and "I have very little freedom to decide how I do my work." Each item was measured using a four-point Likert scale that included "strongly disagree," "disagree," "agree," or "strongly agree." Participants who responded with "agree" or "strongly agree" at least once during the interview were considered to have experience in physical labor and low-control jobs, respectively. Participants who had never engaged in paid work during the study period were excluded from the models with job characteristics interactions.

Appendix S4. Model specification

1. FE model without IV

The FE model was specified as follows:

$$Y_{ijt} = \beta_1 R_{ijt} + \beta_2 age_{ijt} + \beta_3 age_{ijt}^2 + \beta_4 mstat_{ijt} + \alpha_i + \lambda_t + u_j * \lambda_t + \varepsilon_{ijt}$$

where Y_{ijt} represents health outcomes and health behaviors as risk factors, and R_{ijt} indicates whether individual *i* residing in country *j* was retired in interviewed year *t*. The variable age_{ijt} denotes the participant's age in years, while $mstat_{ijt}$ indicates marital status. α_i and λ_t represent individual and survey year FEs, respectively. The model also includes $u_j * \lambda_t$, an interaction term between country and year FEs. ε_{ijt} is the error term.

2. FEIV model

In the first stage, the probability of retirement was predicted as follows:

$$R_{ijt} = \beta_1 age_{ijt} + \beta_2 age_{ijt}^2 + \beta_3 ERA_{ijt} + \beta_4 ERA * age_{ijt} + \beta_5 ERA * age_{ijt}^2 + \beta_6 ORA_{ijt} + \beta_7 ORA * age_{ijt} + \beta_8 ORA * age_{ijt}^2 + \beta_9 mstat_{ijt} + \alpha_i + \lambda_t + u_j * \lambda_t + \varepsilon_{ijt}$$

where ERA_{ijt} and ORA_{ijt} are instruments indicating whether the participants reached the ERA and the ORA. The model incorporates interactions between the age function and the IVs because the slopes of the retirement

---

[3] Although Poland did not meet the threshold for high-income countries in 2006, we categorized it as a high-income country through the study period.

[4] Organisation for Economic Co-operation and Development and World Health Organization. 2020. Health at a Glance: Asia/Pacific 2020: Measuring Progress Towards Universal Health Coverage. Paris: OECD Publishing. https://www.oecd-ilibrary.org/social-issues-migration-health/health-at-a-glance-asia-pacific-2020_26b007cd-en.

[5] The question was not included in wave 1 of the ELSA, CRELES, MHAS, and CHARLS.

[6] The question was not included in wave 1 of the ELSA; CRELES; MHAS; and waves 1 through 7 of HRS, CHARLS, JSTAR, and KLoSA.

probability vary after reaching SPAs as shown in Figures S4–5. Subsequently, the second stage was estimated using the following equation:

$$Y_{ijt} = \gamma_1 \hat{R}_{ijt} + \gamma_2 age_{ijt} + \gamma_3 age_{ijt}^2 + \gamma_4 mstat_{ijt} + \alpha_i + \lambda_t + u_j * \lambda_t + v_{ijt}$$

where $\hat{R}_{ijt}$ is the predicted probability of retirement from the first-stage estimation, and v_{ijt} is an error term. Robust standard errors clustering for individual, year, and interactions between country and year were estimated.

Table S4. Comparison between participants followed up and those lost to follow-up

Characteristics in the previous interview	Lost to follow-up		Followed up		Standardized difference
	Mean (obs.)	SD (%)	Mean (obs.)	SD (%)	
Retired	0.487	0.500	0.441	0.497	0.092
Age	61.28	5.406	60.44	5.509	0.154
Men	0.525	0.499	0.494	0.500	0.061
Married	0.783	0.412	0.783	0.413	0.001
Education					0.098
Low	(13,700)	(31.7)	(79,318)	(27.4)	
Middle	(19,175)	(44.3)	(139,831)	(48.2)	
High	(10,407)	(24.0)	(70,693)	(24.4)	
Physically demanding job	0.537	0.499	0.559	0.497	-0.043
Low control job	0.354	0.478	0.342	0.474	0.025
Cognitive function (z-score)	-0.011	1.019	0.063	0.972	-0.074
Physical independence	0.918	0.275	0.925	0.264	-0.026
Self-rated health (z-score)	0.003	1.007	0.112	0.961	-0.110
Physical inactivity	0.180	0.384	0.190	0.392	-0.026
Smoking	0.226	0.418	0.196	0.397	0.072
Binge drinking	0.085	0.278	0.086	0.280	-0.003

Note: In general, a standardized difference less than 0.1 indicates a well balance between the two groups.

Figure S6. Country-by-country associations between retirement and cognitive function

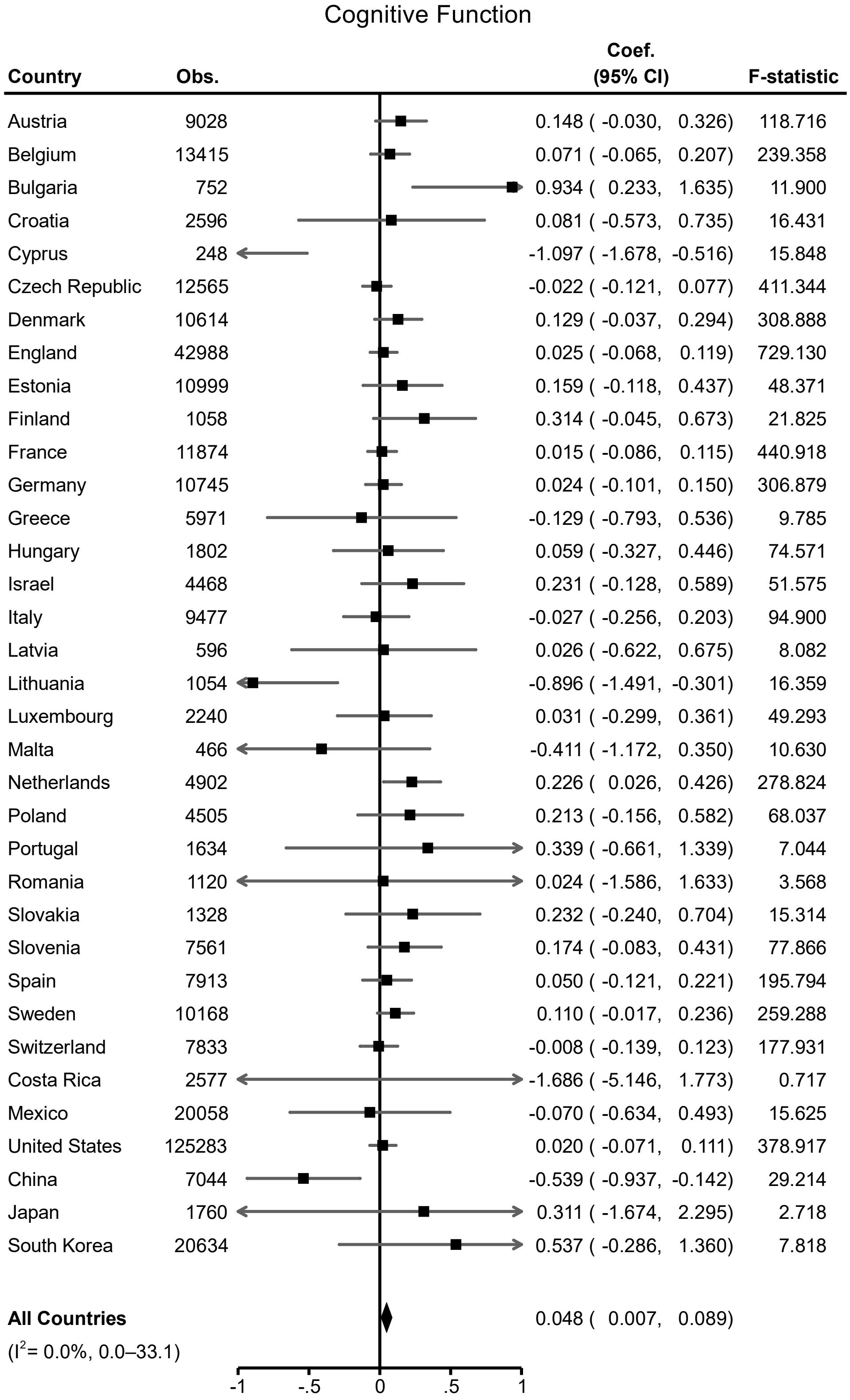

Note: All regressions are adjusted for age, age squared, marital status, and FEs of individual and year. Robust standard errors clustering at individual and year are calculated.

Figure S7. Country-by-country associations between retirement and physical independence

Physical Independence

Country	Obs.	Coef. (95% CI)	F-statistic
Austria	9402	0.010 (-0.030, 0.051)	124.056
Belgium	13481	0.031 (-0.005, 0.067)	242.973
Bulgaria	738	-0.244 (-0.411, -0.076)	12.543
Croatia	2620	0.049 (-0.118, 0.216)	16.425
Cyprus	244	0.093 (-0.007, 0.193)	16.346
Czech Republic	12866	-0.005 (-0.031, 0.021)	412.966
Denmark	10670	0.017 (-0.016, 0.050)	310.583
England	44684	0.029 (0.001, 0.057)	765.277
Estonia	11707	0.126 (0.046, 0.205)	53.531
Finland	1094	-0.093 (-0.182, -0.005)	21.660
France	12034	0.004 (-0.017, 0.025)	447.183
Germany	10836	0.035 (0.002, 0.068)	307.755
Greece	5991	0.004 (-0.108, 0.115)	10.021
Hungary	1814	0.105 (0.006, 0.204)	79.989
Israel	4701	-0.028 (-0.116, 0.059)	55.968
Italy	9645	0.011 (-0.034, 0.056)	97.435
Latvia	600	-0.061 (-0.215, 0.093)	7.702
Lithuania	1046	0.032 (-0.090, 0.155)	16.707
Luxembourg	2342	-0.047 (-0.098, 0.005)	54.277
Malta	472	0.031 (-0.143, 0.205)	10.549
Netherlands	4937	-0.044 (-0.094, 0.005)	282.648
Poland	4549	0.043 (-0.065, 0.151)	67.549
Portugal	1755	-0.024 (-0.289, 0.241)	7.667
Romania	1092	-0.098 (-0.623, 0.426)	3.560
Slovakia	1308	0.179 (0.025, 0.333)	14.457
Slovenia	7767	0.032 (-0.022, 0.085)	83.140
Spain	8160	-0.020 (-0.055, 0.015)	202.179
Sweden	10230	-0.003 (-0.029, 0.023)	260.822
Switzerland	7880	0.007 (-0.017, 0.032)	179.354
Costa Rica	2599	0.545 (-0.344, 1.434)	1.142
Mexico	18245	-0.029 (-0.201, 0.142)	14.812
United States	105591	0.043 (0.007, 0.079)	298.573
China	7963	-0.064 (-0.202, 0.075)	35.715
Japan	2384	0.285 (-0.019, 0.589)	5.565
South Korea	21526	0.126 (-0.074, 0.326)	7.247
**All Countries** (I^2= 40.5%, 0.1–59.8)		0.027 (0.015, 0.038)	

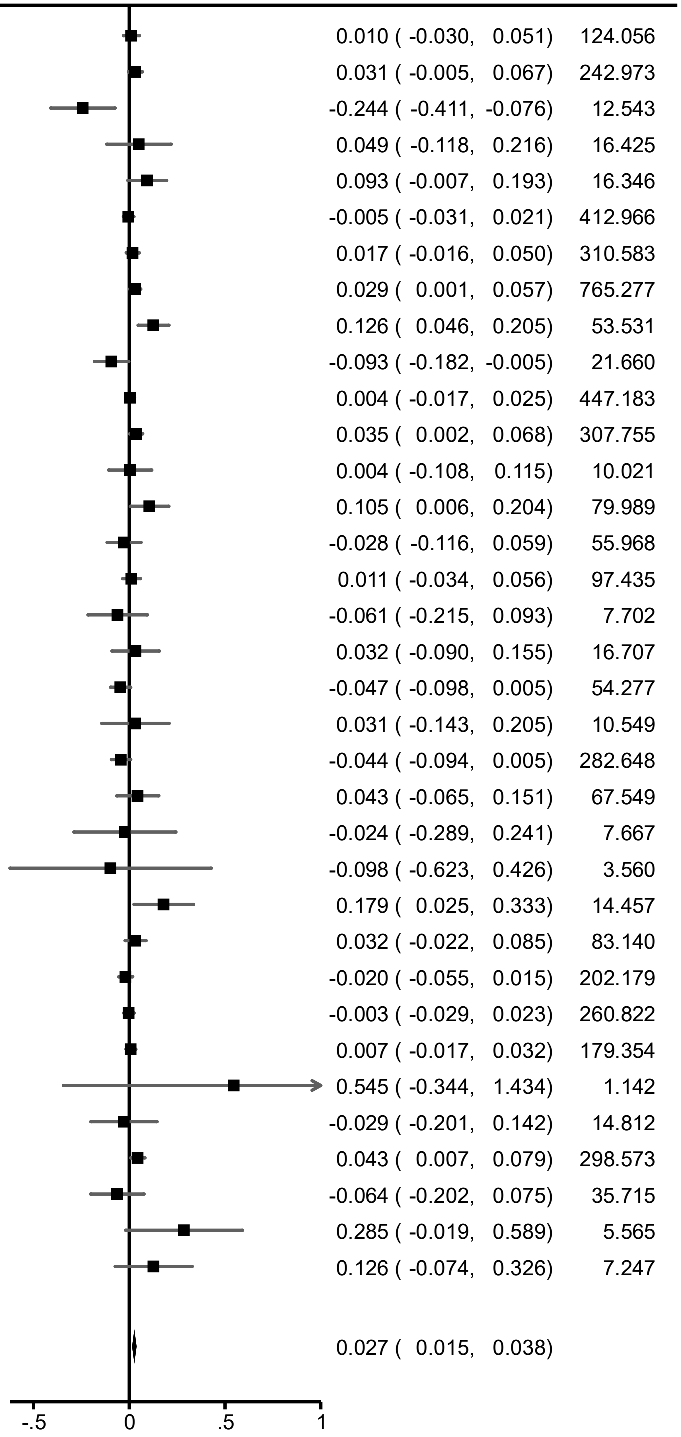

Note: All regressions are adjusted for age, age squared, marital status, and FEs of individual and year. Robust standard errors clustering at individual and year are calculated.

Figure S8. Country-by-country associations between retirement and self-rated health

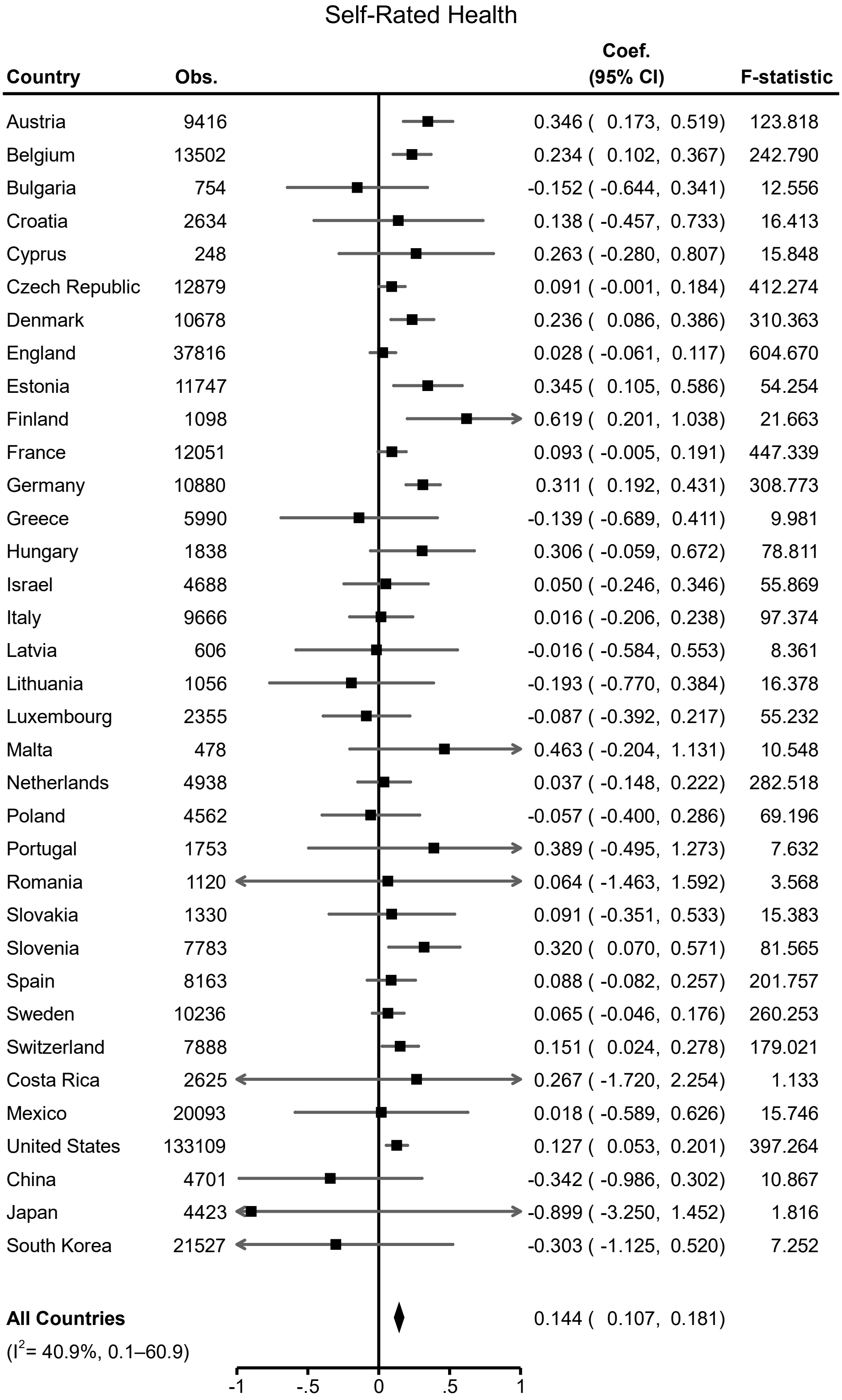

Country	Obs.	Coef. (95% CI)	F-statistic
Austria	9416	0.346 (0.173, 0.519)	123.818
Belgium	13502	0.234 (0.102, 0.367)	242.790
Bulgaria	754	-0.152 (-0.644, 0.341)	12.556
Croatia	2634	0.138 (-0.457, 0.733)	16.413
Cyprus	248	0.263 (-0.280, 0.807)	15.848
Czech Republic	12879	0.091 (-0.001, 0.184)	412.274
Denmark	10678	0.236 (0.086, 0.386)	310.363
England	37816	0.028 (-0.061, 0.117)	604.670
Estonia	11747	0.345 (0.105, 0.586)	54.254
Finland	1098	0.619 (0.201, 1.038)	21.663
France	12051	0.093 (-0.005, 0.191)	447.339
Germany	10880	0.311 (0.192, 0.431)	308.773
Greece	5990	-0.139 (-0.689, 0.411)	9.981
Hungary	1838	0.306 (-0.059, 0.672)	78.811
Israel	4688	0.050 (-0.246, 0.346)	55.869
Italy	9666	0.016 (-0.206, 0.238)	97.374
Latvia	606	-0.016 (-0.584, 0.553)	8.361
Lithuania	1056	-0.193 (-0.770, 0.384)	16.378
Luxembourg	2355	-0.087 (-0.392, 0.217)	55.232
Malta	478	0.463 (-0.204, 1.131)	10.548
Netherlands	4938	0.037 (-0.148, 0.222)	282.518
Poland	4562	-0.057 (-0.400, 0.286)	69.196
Portugal	1753	0.389 (-0.495, 1.273)	7.632
Romania	1120	0.064 (-1.463, 1.592)	3.568
Slovakia	1330	0.091 (-0.351, 0.533)	15.383
Slovenia	7783	0.320 (0.070, 0.571)	81.565
Spain	8163	0.088 (-0.082, 0.257)	201.757
Sweden	10236	0.065 (-0.046, 0.176)	260.253
Switzerland	7888	0.151 (0.024, 0.278)	179.021
Costa Rica	2625	0.267 (-1.720, 2.254)	1.133
Mexico	20093	0.018 (-0.589, 0.626)	15.746
United States	133109	0.127 (0.053, 0.201)	397.264
China	4701	-0.342 (-0.986, 0.302)	10.867
Japan	4423	-0.899 (-3.250, 1.452)	1.816
South Korea	21527	-0.303 (-1.125, 0.520)	7.252
**All Countries** (I^2= 40.9%, 0.1–60.9)		0.144 (0.107, 0.181)	

Note: All regressions are adjusted for age, age squared, marital status, and FEs of individual and year. Robust standard errors clustering at individual and year are calculated.

Figure S9. Country-by-country associations between retirement and physical inactivity

Physical Inactivity

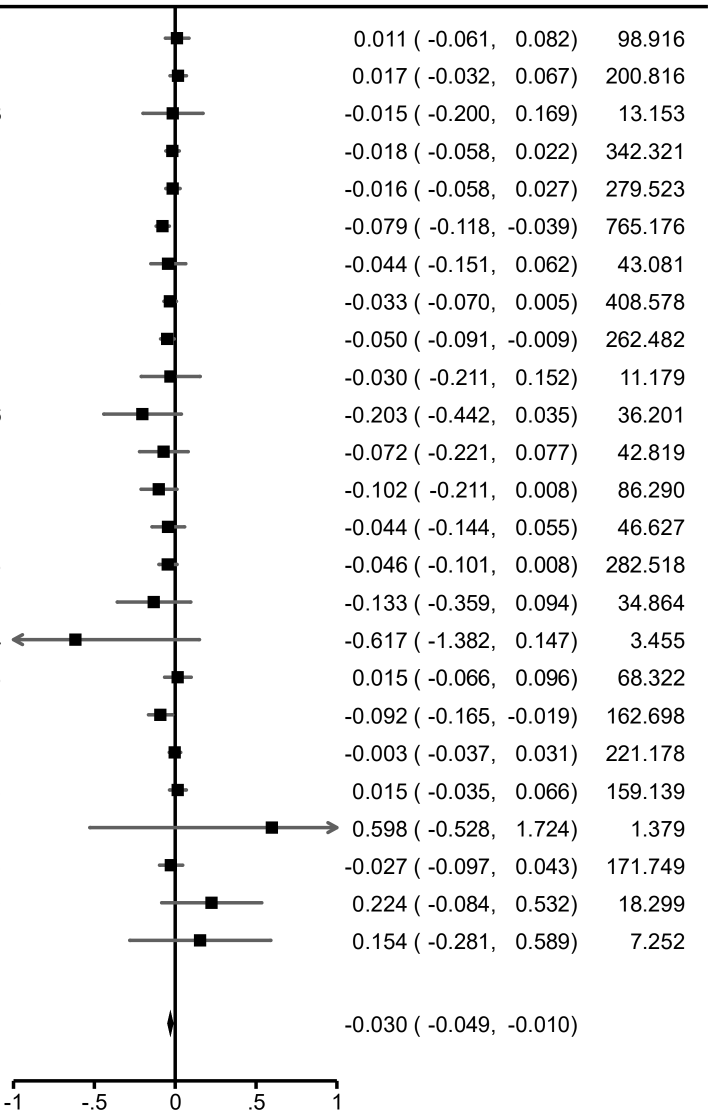

Country	Obs.	Coef. (95% CI)	F-statistic
Austria	8039	0.011 (-0.061, 0.082)	98.916
Belgium	11333	0.017 (-0.032, 0.067)	200.816
Croatia	858	-0.015 (-0.200, 0.169)	13.153
Czech Republic	11029	-0.018 (-0.058, 0.022)	342.321
Denmark	9222	-0.016 (-0.058, 0.027)	279.523
England	44647	-0.079 (-0.118, -0.039)	765.176
Estonia	9009	-0.044 (-0.151, 0.062)	43.081
France	10725	-0.033 (-0.070, 0.005)	408.578
Germany	9061	-0.050 (-0.091, -0.009)	262.482
Greece	5403	-0.030 (-0.211, 0.152)	11.179
Hungary	616	-0.203 (-0.442, 0.035)	36.201
Israel	3782	-0.072 (-0.221, 0.077)	42.819
Italy	7937	-0.102 (-0.211, 0.008)	86.290
Luxembourg	1624	-0.044 (-0.144, 0.055)	46.627
Netherlands	4938	-0.046 (-0.101, 0.008)	282.518
Poland	3070	-0.133 (-0.359, 0.094)	34.864
Portugal	1134	-0.617 (-1.382, 0.147)	3.455
Slovenia	5438	0.015 (-0.066, 0.096)	68.322
Spain	6613	-0.092 (-0.165, -0.019)	162.698
Sweden	9150	-0.003 (-0.037, 0.031)	221.178
Switzerland	7015	0.015 (-0.035, 0.066)	159.139
Costa Rica	1870	0.598 (-0.528, 1.724)	1.379
United States	75515	-0.027 (-0.097, 0.043)	171.749
China	3269	0.224 (-0.084, 0.532)	18.299
South Korea	21527	0.154 (-0.281, 0.589)	7.252
**All Countries** (I^2= 27.5%, 0.0–55.6)		-0.030 (-0.049, -0.010)	

Note: All regressions are adjusted for age, age squared, marital status, and FEs of individual and year. Robust standard errors clustering at individual and year are calculated.

Figure S10. Country-by-country associations between retirement and smoking

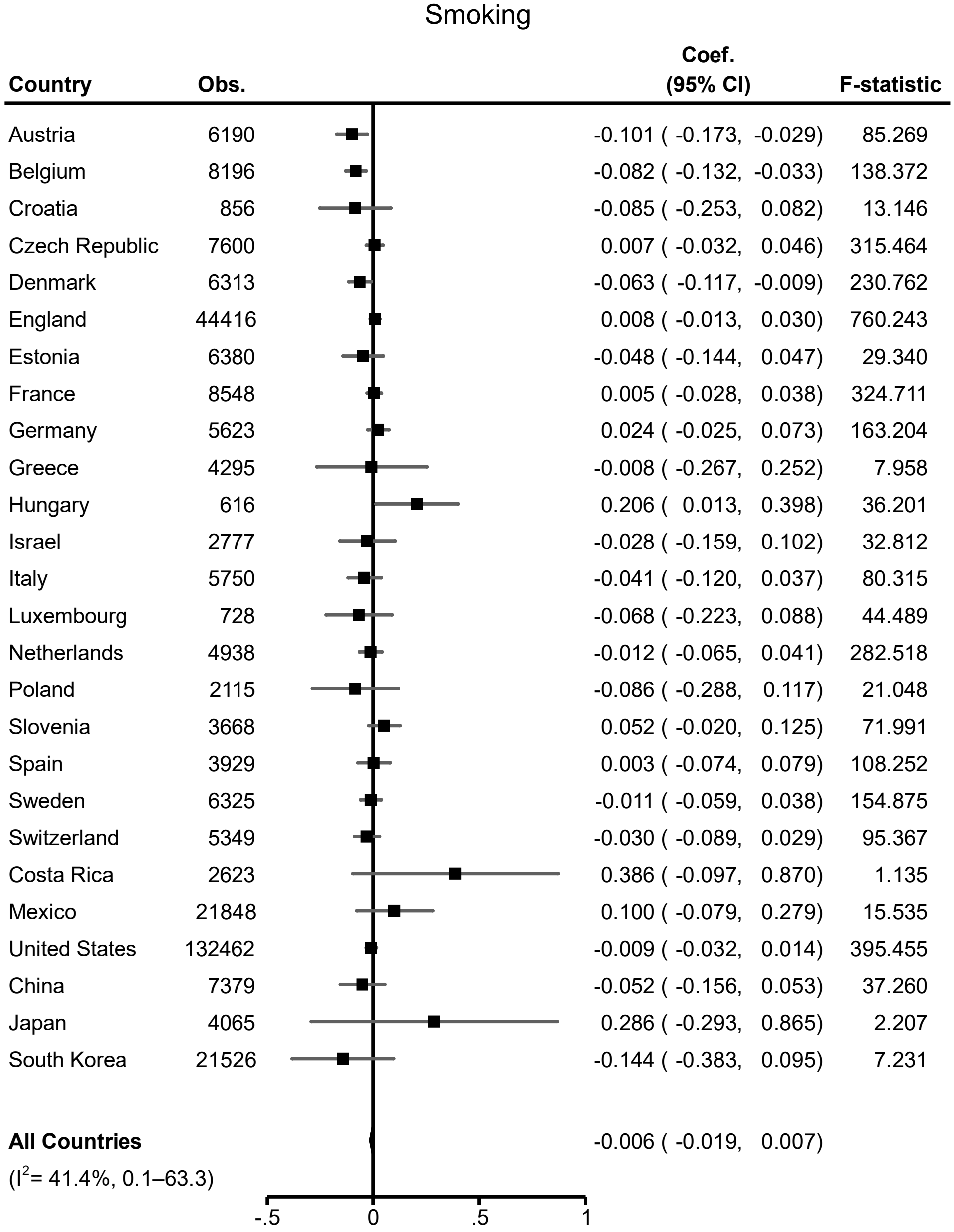

Note: All regressions are adjusted for age, age squared, marital status, and FEs of individual and year. Robust standard errors clustering at individual and year are calculated.

Figure S11. Country-by-country associations between retirement and binge drinking

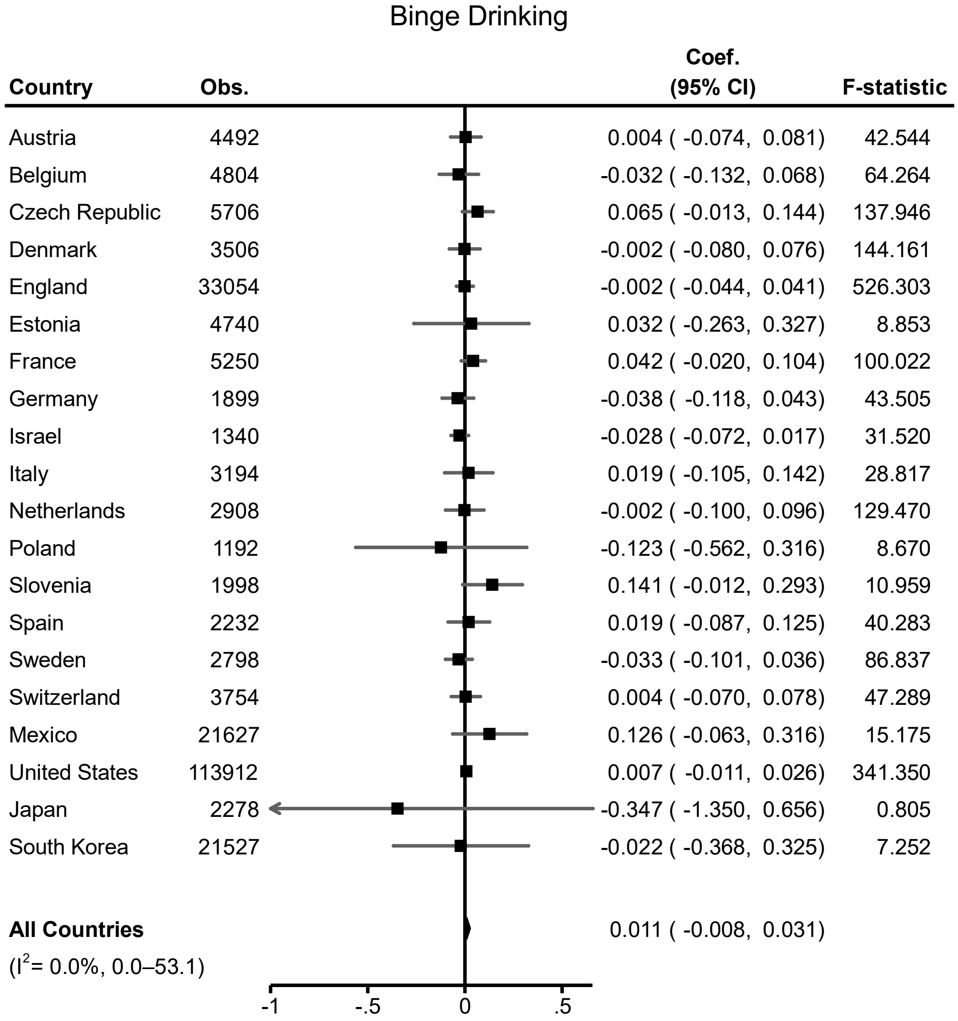

Note: All regressions are adjusted for age, age squared, marital status, and FEs of individual and year. Robust standard errors clustering at individual and year are calculated.

Table S5. FEIV models with interactions of region

Outcome	Obs.	Variable	Coef.	95% CI		P-value
Cognitive function	377276	Retire	0.055	0.015	0.095	0.007
		Retire x America	-0.022	-0.122	0.078	0.661
		Retire x Asia	0.129	-0.252	0.509	0.507
Physical independence	362973	Retire	0.019	0.009	0.029	0.000
		Retire x America	0.025	-0.012	0.063	0.187
		Retire x Asia	-0.034	-0.148	0.080	0.557
Self-rated health	384631	Retire	0.138	0.100	0.175	<0.001
		Retire x America	-0.021	-0.105	0.063	0.628
		Retire x Asia	-0.184	-0.773	0.404	0.540
Physical inactivity	272824	Retire	-0.036	-0.052	-0.020	<0.001
		Retire x America	0.006	-0.066	0.078	0.862
		Retire x Asia	0.019	-0.237	0.274	0.886
Smoking	324519	Retire	-0.010	-0.023	0.003	0.138
		Retire x America	0.004	-0.023	0.031	0.799
		Retire x Asia	-0.058	-0.158	0.043	0.259
Binge drinking	242211	Retire	0.006	-0.020	0.032	0.658
		Retire x America	0.002	-0.031	0.034	0.914
		Retire x Asia	-0.018	-0.354	0.319	0.918

Note: European countries are set to the reference group. All regressions are adjusted for covariates (age, age squared, and marital status), interactions between covariates and regions, and FEs of individual, year, and interactions between country and year. Robust standard errors clustering at individual, year, and interactions between country and year are calculated.

Table S6. FEIV models with an interaction of country income

Outcome	Obs.	Variable	Coef.	95% CI		P-value
Cognitive function	377276	Retire	0.047	0.006	0.087	0.024
		Retire x LMIC	-0.198	-0.519	0.123	0.226
Physical independence	362973	Retire	0.030	0.018	0.042	<0.001
		Retire x LMIC	-0.043	-0.158	0.073	0.469
Self-rated health	384631	Retire	0.151	0.114	0.188	<0.001
		Retire x LMIC	-0.258	-0.721	0.205	0.275
Physical inactivity	272824	Retire	-0.029	-0.048	-0.009	<0.001
		Retire x LMIC	0.139	-0.150	0.427	0.347
Smoking	324519	Retire	-0.006	-0.019	0.006	0.328
		Retire x LMIC	-0.008	-0.099	0.084	0.869
Binge drinking	242211	Retire	0.013	-0.007	0.032	0.199
		Retire x LMIC	0.114	-0.077	0.304	0.242

Note: LMIC denotes low-middle income country. All regressions are adjusted for covariates (age, age squared, and marital status), interactions between covariates and LMIC, and FEs of individual, year, and interactions between country and year. Robust standard errors clustering at individual, year, and interactions between country and year are calculated.

Table S7. FEIV models with an interaction of an aged society

Outcome	Obs.	Variable	Coef.	95% CI		P-value
Cognitive function	377276	Retire	0.032	-0.064	0.128	0.516
		Retire x Aged	0.018	-0.085	0.122	0.727
Physical independence	362973	Retire	0.043	0.007	0.079	0.020
		Retire x Aged	-0.022	-0.060	0.016	0.252
Self-rated health	384631	Retire	0.096	0.014	0.178	0.022
		Retire x Aged	0.042	-0.048	0.132	0.361
Physical inactivity	272824	Retire	0.009	-0.078	0.096	0.837
		Retire x Aged	-0.046	-0.135	0.043	0.310
Smoking	324519	Retire	0.004	-0.021	0.029	0.745
		Retire x Aged	-0.012	-0.041	0.016	0.397
Binge drinking	242211	Retire	0.002	-0.022	0.025	0.886
		Retire x Aged	0.010	-0.024	0.045	0.559

Note: An aged society is a country where the percentage of the population aged 65 years and older exceeds 14%. All regressions are adjusted for covariates (age, age squared, and marital status), interactions between covariates and an aged society, and FEs of individual, year, and interactions between country and year. Robust standard errors clustering at individual, year, and interactions between country and year are calculated.

Table S8. FEIV models for cognitive function with an interaction of self-employment

Country	Obs.	Variable	Coef.	95% CI		P-value
Austria	4734	Retire	0.205	0.018	0.391	0.031
		Retire x Self-employed	0.027	-0.407	0.461	0.904
Belgium	8988	Retire	0.069	-0.131	0.269	0.496
		Retire x Self-employed	-0.110	-0.543	0.322	0.617
Bulgaria	400	Retire	0.067	-0.483	0.618	0.810
		Retire x Self-employed	0.243	-1.476	1.961	0.781
Croatia	1037	Retire	0.064	-0.600	0.728	0.850
		Retire x Self-employed	-2.714	-6.915	1.487	0.205
Cyprus	136	Retire	-0.626	-1.062	-0.191	0.005
		Retire x Self-employed	0.555	-0.289	1.398	0.196
Czech Republic	7519	Retire	0.013	-0.093	0.119	0.815
		Retire x Self-employed	0.050	-0.222	0.321	0.720
Denmark	8863	Retire	0.240	0.012	0.468	0.039
		Retire x Self-employed	-0.522	-1.309	0.264	0.193
England	34113	Retire	0.069	-0.047	0.186	0.241
		Retire x Self-employed	-0.374	-0.745	-0.003	0.048
Estonia	8169	Retire	0.174	-0.192	0.541	0.351
		Retire x Self-employed	-0.262	-1.448	0.924	0.665
Finland	676	Retire	0.077	-0.227	0.381	0.619
		Retire x Self-employed	-0.006	-0.721	0.710	0.988
France	7805	Retire	-0.024	-0.144	0.096	0.695
		Retire x Self-employed	0.199	-0.186	0.583	0.311
Germany	8261	Retire	-0.004	-0.149	0.141	0.957
		Retire x Self-employed	0.337	-0.133	0.808	0.160
Greece	4202	Retire	0.455	-0.679	1.589	0.432
		Retire x Self-employed	-0.853	-2.424	0.718	0.287

Hungary	941	Retire	0.323	-0.076	0.722	0.113
		Retire x Self-employed	0.108	-1.593	1.808	0.901
Israel	3872	Retire	0.103	-0.248	0.454	0.565
		Retire x Self-employed	-0.131	-1.646	1.383	0.865
Italy	5773	Retire	-0.168	-0.507	0.170	0.330
		Retire x Self-employed	-0.250	-0.862	0.363	0.425
Latvia	354	Retire	0.270	-0.388	0.928	0.420
		Retire x Self-employed	–	–	–	–
Lithuania	740	Retire	-1.022	-1.627	-0.417	0.001
		Retire x Self-employed	0.780	-0.570	2.129	0.257
Luxembourg	1215	Retire	-0.164	-0.622	0.294	0.483
		Retire x Self-employed	0.595	-0.557	1.747	0.311
Malta	258	Retire	-0.471	-1.223	0.282	0.219
		Retire x Self-employed	–	–	–	–
Netherlands	4015	Retire	0.218	-0.053	0.489	0.114
		Retire x Self-employed	0.206	-0.532	0.944	0.585
Poland	2468	Retire	0.286	-0.019	0.590	0.066
		Retire x Self-employed	-1.443	-2.646	-0.240	0.019
Portugal	868	Retire	1.070	-0.428	2.568	0.161
		Retire x Self-employed	-0.327	-1.940	1.286	0.691
Romania	334	Retire	-0.169	-1.510	1.173	0.805
		Retire x Self-employed	-0.624	-2.052	0.805	0.391
Slovakia	816	Retire	0.431	-0.023	0.885	0.063
		Retire x Self-employed	–	–	–	–
Slovenia	2904	Retire	0.162	-0.089	0.412	0.206
		Retire x Self-employed	-0.166	-1.044	0.713	0.711
Spain	5801	Retire	-0.143	-0.388	0.101	0.250
		Retire x Self-employed	0.214	-0.190	0.617	0.300
Sweden	8332	Retire	0.079	-0.062	0.220	0.273
		Retire x Self-employed	0.239	-0.215	0.693	0.303
Switzerland	6732	Retire	-0.047	-0.194	0.099	0.525
		Retire x Self-employed	0.001	-0.441	0.442	0.997
Costa Rica	2237	Retire	0.351	-1.636	2.338	0.729
		Retire x Self-employed	2.672	-3.447	8.791	0.392
Mexico	18911	Retire	-0.154	-0.600	0.293	0.500
		Retire x Self-employed	-0.278	-1.638	1.081	0.688
United States	107578	Retire	0.067	-0.030	0.163	0.177
		Retire x Self-employed	-0.183	-0.416	0.050	0.124
China	4175	Retire	-0.151	-0.488	0.186	0.380
		Retire x Self-employed	-1.431	-3.653	0.792	0.207
Japan	1620	Retire	-1.255	-3.009	0.499	0.161
		Retire x Self-employed	2.273	-1.404	5.950	0.225
South Korea	17662	Retire	0.001	-0.775	0.777	0.998
		Retire x Self-employed	-0.817	-2.715	1.080	0.398

Note: All regressions are adjusted for age, age squared, marital status, and FEs of individual and year. Robust standard errors clustering at individual and year are calculated. In Latvia, Malta, and Slovakia, the interaction term was not estimated due to collinearity.

Table S9. FEIV models for physical independence with an interaction of self-employment

Country	Obs.	Variable	Coef.	95% CI		P-value
Austria	4957	Retire	0.055	0.014	0.097	0.009
		Retire x Self-employed	-0.089	-0.158	-0.021	0.011
Belgium	9024	Retire	0.016	-0.033	0.066	0.512
		Retire x Self-employed	-0.101	-0.200	-0.001	0.047
Bulgaria	394	Retire	-0.111	-0.210	-0.011	0.030
		Retire x Self-employed	-0.028	-0.306	0.250	0.843
Croatia	1038	Retire	0.031	-0.028	0.091	0.301
		Retire x Self-employed	1.347	-0.870	3.563	0.233
Cyprus	132	Retire	-0.057	-0.140	0.027	0.180
		Retire x Self-employed	0.053	-0.026	0.132	0.189
Czech Republic	7682	Retire	-0.007	-0.030	0.017	0.594
		Retire x Self-employed	-0.013	-0.066	0.040	0.623
Denmark	8904	Retire	0.001	-0.036	0.038	0.955
		Retire x Self-employed	0.189	-0.014	0.393	0.068
England	35506	Retire	0.008	-0.025	0.042	0.621
		Retire x Self-employed	0.067	-0.021	0.155	0.134
Estonia	8701	Retire	0.082	-0.013	0.177	0.089
		Retire x Self-employed	0.081	-0.243	0.405	0.626
Finland	690	Retire	-0.014	-0.065	0.038	0.602
		Retire x Self-employed	-0.021	-0.211	0.170	0.832
France	7922	Retire	0.008	-0.017	0.033	0.543
		Retire x Self-employed	-0.020	-0.094	0.054	0.597
Germany	8313	Retire	0.024	-0.013	0.060	0.200
		Retire x Self-employed	-0.096	-0.209	0.017	0.096
Greece	4207	Retire	-0.125	-0.243	-0.007	0.038
		Retire x Self-employed	0.111	-0.051	0.272	0.178
Hungary	953	Retire	-0.008	-0.116	0.100	0.888
		Retire x Self-employed	0.082	-0.118	0.282	0.423
Israel	4066	Retire	-0.033	-0.119	0.053	0.456
		Retire x Self-employed	0.146	-0.125	0.418	0.291
Italy	5881	Retire	0.026	-0.038	0.091	0.422
		Retire x Self-employed	-0.020	-0.109	0.069	0.659
Latvia	360	Retire	-0.182	-0.363	-0.001	0.048
		Retire x Self-employed	–	–	–	–
Lithuania	732	Retire	0.029	-0.069	0.127	0.562
		Retire x Self-employed	0.025	-0.139	0.189	0.767
Luxembourg	1262	Retire	-0.056	-0.119	0.006	0.078
		Retire x Self-employed	0.049	-0.015	0.113	0.134
Malta	260	Retire	-0.033	-0.079	0.014	0.169
		Retire x Self-employed	–	–	–	–
Netherlands	4042	Retire	-0.075	-0.138	-0.012	0.021
		Retire x Self-employed	-0.104	-0.282	0.074	0.252
Poland	2477	Retire	0.053	-0.019	0.125	0.148
		Retire x Self-employed	-0.112	-0.416	0.193	0.472
Portugal	919	Retire	0.490	-0.025	1.004	0.062
		Retire x Self-employed	-0.532	-1.075	0.010	0.055
Romania	332	Retire	-0.273	-0.716	0.169	0.225
		Retire x Self-employed	0.245	-0.188	0.679	0.266

Slovakia	798	Retire	0.002	-0.061	0.065	0.949
		Retire x Self-employed	–	–	–	–
Slovenia	2991	Retire	0.037	-0.014	0.088	0.153
		Retire x Self-employed	0.018	-0.178	0.213	0.860
Spain	5944	Retire	-0.047	-0.084	-0.009	0.016
		Retire x Self-employed	0.087	0.022	0.151	0.009
Sweden	8374	Retire	-0.014	-0.041	0.012	0.293
		Retire x Self-employed	-0.026	-0.100	0.048	0.495
Switzerland	6768	Retire	0.010	-0.017	0.037	0.461
		Retire x Self-employed	-0.029	-0.109	0.051	0.484
Costa Rica	2231	Retire	0.426	-0.347	1.199	0.280
		Retire x Self-employed	-1.898	-3.746	-0.050	0.044
Mexico	17146	Retire	0.001	-0.126	0.127	0.992
		Retire x Self-employed	0.045	-0.363	0.452	0.829
United States	91518	Retire	0.041	0.003	0.078	0.032
		Retire x Self-employed	0.011	-0.075	0.097	0.808
China	4735	Retire	-0.044	-0.165	0.076	0.471
		Retire x Self-employed	-0.208	-0.983	0.567	0.598
Japan	2192	Retire	-0.099	-0.236	0.038	0.155
		Retire x Self-employed	0.107	-0.333	0.547	0.633
South Korea	18389	Retire	0.105	-0.076	0.285	0.255
		Retire x Self-employed	0.054	-0.421	0.530	0.824

Note: All regressions are adjusted for age, age squared, marital status, and FEs of individual and year. Robust standard errors clustering at individual and year are calculated. In Latvia, Malta, and Slovakia, the interaction term was not estimated due to collinearity.

Table S10. FEIV models for self-rated health with an interaction of self-employment

Country	Obs.	Variable	Coef.	95% CI		P-value
Austria	4959	Retire	0.333	0.144	0.522	0.001
		Retire x Self-employed	-0.200	-0.590	0.190	0.316
Belgium	9039	Retire	0.194	0.000	0.388	0.050
		Retire x Self-employed	0.135	-0.322	0.592	0.563
Bulgaria	402	Retire	0.281	-0.259	0.821	0.307
		Retire x Self-employed	-3.167	-8.063	1.728	0.204
Croatia	1042	Retire	0.103	-0.487	0.692	0.733
		Retire x Self-employed	1.676	-2.560	5.911	0.438
Cyprus	136	Retire	0.224	-0.343	0.790	0.436
		Retire x Self-employed	0.289	-0.842	1.420	0.614
Czech Republic	7690	Retire	0.130	0.032	0.227	0.009
		Retire x Self-employed	-0.270	-0.507	-0.033	0.026
Denmark	8912	Retire	0.214	0.009	0.419	0.041
		Retire x Self-employed	0.257	-0.490	1.005	0.500
England	30254	Retire	-0.002	-0.113	0.109	0.970
		Retire x Self-employed	0.004	-0.320	0.328	0.982
Estonia	8729	Retire	0.565	0.221	0.908	0.001
		Retire x Self-employed	-1.057	-2.169	0.054	0.062
Finland	694	Retire	0.499	0.048	0.950	0.030
		Retire x Self-employed	0.183	-0.569	0.935	0.634

France	7933	Retire	0.178	0.056	0.299	0.004
		Retire x Self-employed	-0.242	-0.592	0.108	0.176
Germany	8349	Retire	0.276	0.139	0.413	0.000
		Retire x Self-employed	-0.086	-0.557	0.384	0.719
Greece	4208	Retire	1.092	0.117	2.066	0.028
		Retire x Self-employed	-1.306	-2.592	-0.020	0.047
Hungary	966	Retire	0.245	-0.157	0.647	0.232
		Retire x Self-employed	-0.669	-1.971	0.634	0.314
Israel	4053	Retire	-0.168	-0.478	0.142	0.288
		Retire x Self-employed	0.647	-0.492	1.787	0.266
Italy	5896	Retire	0.249	-0.078	0.575	0.135
		Retire x Self-employed	-0.158	-0.738	0.423	0.594
Latvia	362	Retire	0.033	-0.568	0.634	0.914
		Retire x Self-employed	–	–	–	–
Lithuania	742	Retire	0.186	-0.379	0.752	0.518
		Retire x Self-employed	-1.157	-2.092	-0.222	0.015
Luxembourg	1270	Retire	-0.266	-0.734	0.202	0.265
		Retire x Self-employed	1.224	0.053	2.395	0.041
Malta	264	Retire	-0.172	-0.824	0.481	0.605
		Retire x Self-employed	–	–	–	–
Netherlands	4043	Retire	0.057	-0.198	0.312	0.663
		Retire x Self-employed	0.140	-0.518	0.798	0.677
Poland	2489	Retire	0.105	-0.176	0.386	0.463
		Retire x Self-employed	-0.571	-1.481	0.339	0.219
Portugal	914	Retire	-0.134	-1.446	1.178	0.841
		Retire x Self-employed	0.172	-1.239	1.584	0.811
Romania	334	Retire	-0.853	-2.324	0.618	0.255
		Retire x Self-employed	1.732	0.134	3.331	0.034
Slovakia	816	Retire	0.193	-0.233	0.619	0.373
		Retire x Self-employed	–	–	–	–
Slovenia	2993	Retire	0.172	-0.086	0.429	0.191
		Retire x Self-employed	0.481	-0.303	1.265	0.229
Spain	5948	Retire	0.069	-0.172	0.310	0.575
		Retire x Self-employed	0.042	-0.352	0.436	0.836
Sweden	8376	Retire	0.067	-0.056	0.190	0.285
		Retire x Self-employed	-0.083	-0.482	0.317	0.685
Switzerland	6776	Retire	0.086	-0.055	0.227	0.231
		Retire x Self-employed	0.202	-0.255	0.659	0.387
Costa Rica	2252	Retire	0.495	-1.237	2.226	0.575
		Retire x Self-employed	1.812	-2.731	6.355	0.434
Mexico	18946	Retire	-0.335	-0.818	0.148	0.174
		Retire x Self-employed	-0.720	-2.222	0.782	0.347
United States	114365	Retire	0.102	0.025	0.179	0.009
		Retire x Self-employed	-0.066	-0.254	0.123	0.494
China	2728	Retire	0.004	-0.598	0.606	0.990
		Retire x Self-employed	-0.243	-2.467	1.982	0.830
Japan	4079	Retire	-0.370	-3.595	2.856	0.822
		Retire x Self-employed	-1.700	-7.142	3.742	0.540
South Korea	18389	Retire	-0.152	-0.910	0.605	0.693
		Retire x Self-employed	-0.658	-2.772	1.455	0.542

Note: All regressions are adjusted for age, age squared, marital status, and FEs of individual and year. Robust standard errors clustering at individual and year are calculated. In Latvia, Malta, and Slovakia, the interaction term was not estimated due to collinearity.

Table S11. FEIV models for physical inactivity with an interaction of self-employment

Country	Obs.	Variable	Coef.	95% CI		P-value
Austria	4152	Retire	0.037	-0.034	0.108	0.309
		Retire x Self-employed	0.006	-0.121	0.134	0.923
Belgium	7463	Retire	0.029	-0.040	0.099	0.410
		Retire x Self-employed	-0.036	-0.168	0.095	0.587
Croatia	384	Retire	-0.014	-0.173	0.146	0.867
		Retire x Self-employed	0.520	-0.207	1.248	0.160
Czech Republic	6535	Retire	0.004	-0.037	0.046	0.831
		Retire x Self-employed	-0.089	-0.178	0.000	0.051
Denmark	7690	Retire	-0.040	-0.093	0.012	0.128
		Retire x Self-employed	0.195	-0.023	0.412	0.080
England	35480	Retire	-0.063	-0.113	-0.014	0.012
		Retire x Self-employed	0.037	-0.101	0.175	0.601
Estonia	6544	Retire	-0.105	-0.252	0.043	0.164
		Retire x Self-employed	-0.054	-0.487	0.378	0.806
France	6998	Retire	-0.034	-0.081	0.012	0.149
		Retire x Self-employed	0.070	-0.034	0.174	0.187
Germany	6915	Retire	-0.007	-0.054	0.040	0.763
		Retire x Self-employed	-0.238	-0.410	-0.067	0.006
Greece	3828	Retire	0.299	-0.021	0.619	0.067
		Retire x Self-employed	-0.570	-1.012	-0.129	0.011
Hungary	382	Retire	0.130	-0.087	0.346	0.240
		Retire x Self-employed	0.203	-0.480	0.886	0.559
Israel	3251	Retire	-0.012	-0.161	0.138	0.878
		Retire x Self-employed	0.022	-0.791	0.835	0.958
Italy	4703	Retire	-0.067	-0.235	0.101	0.436
		Retire x Self-employed	0.044	-0.251	0.338	0.771
Luxembourg	896	Retire	0.104	-0.065	0.272	0.228
		Retire x Self-employed	-0.135	-0.307	0.038	0.126
Netherlands	4043	Retire	0.055	-0.016	0.126	0.131
		Retire x Self-employed	-0.100	-0.252	0.052	0.196
Poland	1562	Retire	0.021	-0.111	0.153	0.757
		Retire x Self-employed	0.234	-0.263	0.730	0.356
Portugal	528	Retire	-0.237	-0.980	0.506	0.531
		Retire x Self-employed	-0.587	-1.542	0.368	0.228
Slovenia	2115	Retire	-0.032	-0.108	0.044	0.409
		Retire x Self-employed	0.080	-0.150	0.310	0.496
Spain	4807	Retire	-0.046	-0.148	0.056	0.378
		Retire x Self-employed	-0.087	-0.249	0.076	0.296
Sweden	7464	Retire	-0.021	-0.054	0.013	0.221
		Retire x Self-employed	0.103	-0.016	0.222	0.088
Switzerland	6019	Retire	0.022	-0.037	0.081	0.458
		Retire x Self-employed	0.014	-0.139	0.168	0.856

Costa Rica	1628	Retire	0.278	-0.235	0.792	0.288
		Retire x Self-employed	-0.694	-2.095	0.707	0.331
United States	65922	Retire	-0.012	-0.086	0.061	0.742
		Retire x Self-employed	-0.134	-0.283	0.015	0.077
China	1960	Retire	0.070	-0.199	0.339	0.610
		Retire x Self-employed	0.783	-0.362	1.928	0.180
South Korea	18389	Retire	-0.336	-0.734	0.063	0.099
		Retire x Self-employed	0.259	-0.817	1.335	0.637

Note: All regressions are adjusted for age, age squared, marital status, and FEs of individual and year. Robust standard errors clustering at individual and year are calculated.

Table S12. FEIV models for smoking with an interaction of self-employment

Country	Obs.	Variable	Coef.	95% CI		P-value
Austria	3105	Retire	-0.051	-0.133	0.031	0.223
		Retire x Self-employed	-0.104	-0.260	0.052	0.191
Belgium	5352	Retire	-0.094	-0.162	-0.026	0.007
		Retire x Self-employed	0.104	-0.082	0.289	0.273
Croatia	384	Retire	0.007	-0.211	0.224	0.953
		Retire x Self-employed	-0.249	-0.721	0.223	0.300
Czech Republic	4525	Retire	0.000	-0.042	0.042	0.997
		Retire x Self-employed	-0.025	-0.120	0.070	0.609
Denmark	5398	Retire	-0.028	-0.097	0.042	0.435
		Retire x Self-employed	-0.143	-0.468	0.182	0.390
England	35281	Retire	0.023	-0.004	0.049	0.099
		Retire x Self-employed	-0.068	-0.155	0.019	0.124
Estonia	4650	Retire	-0.043	-0.197	0.110	0.579
		Retire x Self-employed	-0.069	-0.336	0.198	0.614
France	5538	Retire	-0.002	-0.043	0.040	0.943
		Retire x Self-employed	0.017	-0.121	0.155	0.807
Germany	4372	Retire	0.013	-0.047	0.073	0.665
		Retire x Self-employed	0.003	-0.194	0.199	0.979
Greece	2944	Retire	-0.206	-0.664	0.251	0.377
		Retire x Self-employed	0.104	-0.503	0.712	0.737
Hungary	382	Retire	0.014	-0.307	0.335	0.931
		Retire x Self-employed	0.576	-0.070	1.223	0.080
Israel	2370	Retire	-0.070	-0.195	0.054	0.266
		Retire x Self-employed	0.346	-0.347	1.039	0.328
Italy	3345	Retire	0.064	-0.054	0.181	0.287
		Retire x Self-employed	0.016	-0.217	0.250	0.892
Luxembourg	488	Retire	0.040	-0.128	0.207	0.643
		Retire x Self-employed	-0.253	-0.698	0.193	0.266
Netherlands	4043	Retire	0.012	-0.064	0.088	0.759
		Retire x Self-employed	0.116	-0.025	0.257	0.108
Poland	1089	Retire	-0.072	-0.229	0.085	0.366
		Retire x Self-employed	0.128	-0.329	0.585	0.582
Slovenia	1466	Retire	0.054	-0.023	0.132	0.166
		Retire x Self-employed	-0.026	-0.217	0.164	0.787
Spain	2901	Retire	0.070	-0.048	0.188	0.243

		Retire x Self-employed	-0.138	-0.323	0.048	0.146
Sweden	5277	Retire	0.018	-0.035	0.071	0.507
		Retire x Self-employed	0.022	-0.164	0.208	0.820
Switzerland	4588	Retire	-0.011	-0.076	0.054	0.735
		Retire x Self-employed	0.080	-0.159	0.319	0.511
Costa Rica	2250	Retire	0.228	-0.148	0.604	0.235
		Retire x Self-employed	-0.075	-0.861	0.711	0.852
Mexico	20621	Retire	0.204	0.044	0.364	0.013
		Retire x Self-employed	-0.254	-0.685	0.176	0.247
United States	113850	Retire	-0.018	-0.043	0.006	0.144
		Retire x Self-employed	0.029	-0.027	0.086	0.311
China	4345	Retire	0.031	-0.072	0.133	0.557
		Retire x Self-employed	-0.598	-1.340	0.144	0.114
Japan	3752	Retire	-0.162	-0.913	0.590	0.673
		Retire x Self-employed	0.486	-0.694	1.667	0.419
South Korea	18388	Retire	-0.122	-0.331	0.086	0.251
		Retire x Self-employed	0.383	-0.255	1.022	0.239

Note: All regressions are adjusted for age, age squared, marital status, and FEs of individual and year. Robust standard errors clustering at individual and year are calculated.

Table S13. FEIV models for binge drinking with an interaction of self-employment

Country	Obs.	Variable	Coef.	95% CI		P-value
Austria	2224	Retire	0.006	-0.070	0.082	0.872
		Retire x Self-employed	-0.154	-0.365	0.056	0.151
Belgium	3216	Retire	-0.094	-0.217	0.029	0.133
		Retire x Self-employed	0.031	-0.219	0.281	0.810
Czech Republic	3103	Retire	0.035	-0.048	0.118	0.408
		Retire x Self-employed	0.224	0.023	0.425	0.029
Denmark	3036	Retire	0.041	-0.067	0.149	0.458
		Retire x Self-employed	0.017	-0.472	0.505	0.946
England	27015	Retire	0.006	-0.042	0.054	0.804
		Retire x Self-employed	-0.040	-0.215	0.135	0.653
Estonia	3228	Retire	-0.555	-0.950	-0.159	0.006
		Retire x Self-employed	0.921	0.302	1.541	0.004
France	3293	Retire	0.074	0.002	0.147	0.045
		Retire x Self-employed	-0.222	-0.429	-0.016	0.034
Germany	1481	Retire	0.010	-0.067	0.088	0.791
		Retire x Self-employed	-0.043	-0.217	0.131	0.626
Israel	1164	Retire	-0.019	-0.064	0.025	0.397
		Retire x Self-employed	0.010	-0.223	0.243	0.931
Italy	1787	Retire	-0.046	-0.176	0.084	0.487
		Retire x Self-employed	0.090	-0.148	0.327	0.460
Netherlands	2362	Retire	-0.071	-0.170	0.027	0.157
		Retire x Self-employed	-0.179	-0.561	0.202	0.357
Poland	534	Retire	0.217	-0.094	0.529	0.171
		Retire x Self-employed	-0.604	-1.149	-0.058	0.030
Slovenia	724	Retire	0.038	-0.112	0.188	0.618
		Retire x Self-employed	0.222	-0.093	0.536	0.167

Spain	1619	Retire	0.016	-0.120	0.152	0.817
		Retire x Self-employed	-0.129	-0.338	0.080	0.227
Sweden	2439	Retire	-0.053	-0.126	0.019	0.148
		Retire x Self-employed	-0.021	-0.214	0.171	0.827
Switzerland	3143	Retire	-0.010	-0.085	0.065	0.791
		Retire x Self-employed	0.035	-0.171	0.240	0.742
Mexico	20403	Retire	0.124	-0.025	0.273	0.103
		Retire x Self-employed	-0.063	-0.512	0.386	0.783
United States	98869	Retire	0.006	-0.014	0.025	0.556
		Retire x Self-employed	0.004	-0.042	0.049	0.866
Japan	2136	Retire	0.397	-0.179	0.973	0.177
		Retire x Self-employed	-0.817	-1.531	-0.104	0.025
South Korea	18389	Retire	0.190	-0.128	0.508	0.242
		Retire x Self-employed	-0.185	-1.092	0.722	0.689

Note: All regressions are adjusted for age, age squared, marital status, and FEs of individual and year. Robust standard errors clustering at individual and year are calculated.

Table S14. First stage estimation of FEIV models

	Cognitive function	Physical independence	Self-rated health	Physical inactivity	Smoking	Binge drinking
Age	0.017***	0.018***	0.016***	0.021***	0.015***	0.013***
	(0.001)	(0.001)	(0.001)	(0.002)	(0.001)	(0.002)
Age^2	0.003***	0.003***	0.003***	0.003***	0.003***	0.002***
	(0.000)	(0.000)	(0.000)	(0.000)	(0.000)	(0.000)
Married	0.007**	0.003	0.005*	-0.001	0.005	0.004
	(0.003)	(0.003)	(0.003)	(0.004)	(0.003)	(0.004)
ERA	0.086***	0.083***	0.084***	0.087***	0.083***	0.064***
	(0.003)	(0.003)	(0.002)	(0.003)	(0.003)	(0.003)
ERA x Age	-0.006***	-0.007***	-0.006***	-0.008***	-0.008***	-0.004***
	(0.001)	(0.001)	(0.001)	(0.001)	(0.001)	(0.001)
ERA x Age^2	-0.000***	-0.000***	-0.000***	-0.000**	-0.000**	-0.000
	(0.000)	(0.000)	(0.000)	(0.000)	(0.000)	(0.000)
ORA	0.180***	0.182***	0.175***	0.180***	0.165***	0.123***
	(0.004)	(0.004)	(0.004)	(0.004)	(0.004)	(0.005)
ORA x Age	-0.010***	-0.009***	-0.009***	-0.004***	-0.009***	-0.005***
	(0.001)	(0.001)	(0.001)	(0.001)	(0.001)	(0.002)
ORA x Age^2	-0.004***	-0.004***	-0.004***	-0.004***	-0.003***	-0.003***
	(0.000)	(0.000)	(0.000)	(0.000)	(0.000)	(0.000)
Observations	377,276	362,973	384,631	272,824	324,519	242,211
Kleibergen-Paap F	2230.315	2222.211	2184.287	1855.314	1592.798	788.561
Sargan-Hansen J	0.684	0.313	2.510	2.471	3.627*	0.640

Note: Robust standard errors clustering at individual, year, and interactions between country and year are shown in parentheses. *** p<0.01, ** p<0.05, * p<0.1.

Table S15. FEIV models with an interaction of gender

Outcome	Obs.	Variable	Coef.	95% CI		P-value
Cognitive function	377276	Retire	0.096	0.044	0.148	<0.001
		Retire x Men	-0.088	-0.168	-0.008	0.031
Physical independence	362973	Retire	0.034	0.019	0.049	<0.001
		Retire x Men	-0.015	-0.038	0.009	0.216
Self-rated health	384631	Retire	0.184	0.137	0.230	<0.001
		Retire x Men	-0.071	-0.145	0.002	0.057
Physical inactivity	272824	Retire	-0.040	-0.064	-0.015	<0.001
		Retire x Men	0.020	-0.018	0.058	0.306
Smoking	324519	Retire	-0.024	-0.038	-0.009	0.002
		Retire x Men	0.048	0.022	0.074	<0.001
Binge drinking	242211	Retire	-0.002	-0.024	0.020	0.877
		Retire x Men	0.024	-0.015	0.063	0.221

Note: All regressions are adjusted for covariates (age, age squared, and marital status), interactions between covariates and gender, and FEs of individual, year, and interactions between country and year. Robust standard errors clustering at individual, year, and interactions between country and year are calculated.

Table S16. FEIV models with interactions of educational levels

Outcome	Obs.	Variable	Coef.	95% CI		P-value
Cognitive function	373853	Retire	0.062	0.007	0.116	0.028
		Retire x Low	-0.059	-0.155	0.037	0.228
		Retire x High	0.027	-0.079	0.133	0.615
Physical independence	359357	Retire	0.021	0.004	0.038	0.013
		Retire x Low	0.002	-0.029	0.032	0.921
		Retire x High	0.008	-0.019	0.034	0.578
Self-rated health	381552	Retire	0.151	0.101	0.200	<0.001
		Retire x Low	-0.032	-0.123	0.059	0.490
		Retire x High	0.037	-0.057	0.131	0.438
Physical inactivity	269364	Retire	-0.025	-0.053	0.004	0.091
		Retire x Low	0.037	-0.011	0.086	0.132
		Retire x High	-0.029	-0.074	0.016	0.205
Smoking	320898	Retire	-0.009	-0.027	0.009	0.341
		Retire x Low	-0.005	-0.037	0.026	0.738
		Retire x High	-0.007	-0.039	0.025	0.663
Binge drinking	239543	Retire	0.008	-0.016	0.032	0.519
		Retire x Low	-0.013	-0.065	0.039	0.627
		Retire x High	0.020	-0.032	0.071	0.455

Note: People with middle-level education are set to the reference group. All regressions are adjusted for covariates (age, age squared, and marital status), interactions between covariates and educational levels, and FEs of individual, year, and interactions between country and year. Robust standard errors clustering at individual, year, and interactions between country and year are calculated.

Table S17. FEIV models with an interaction of physical labor

Outcome	Obs.	Variable	Coef.	95% CI		P-value
Cognitive function	247236	Retire	0.068	-0.004	0.139	0.064
		Retire x Physical	-0.062	-0.160	0.037	0.219
Physical independence	233908	Retire	0.036	0.017	0.054	<0.001
		Retire x Physical	-0.015	-0.042	0.013	0.294
Self-rated health	254372	Retire	0.154	0.091	0.217	<0.001
		Retire x Physical	-0.025	-0.113	0.063	0.576
Physical inactivity	194225	Retire	-0.042	-0.076	-0.009	0.014
		Retire x Physical	0.039	-0.008	0.086	0.101
Smoking	223630	Retire	-0.015	-0.036	0.006	0.165
		Retire x Physical	0.022	-0.007	0.051	0.144
Binge drinking	174681	Retire	0.019	-0.009	0.047	0.179
		Retire x Physical	-0.023	-0.062	0.017	0.266

Note: All regressions are adjusted for covariates (age, age squared, and marital status), interactions between covariates and physical labor, and FEs of individual, year, and interactions between country and year. Robust standard errors clustering at individual, year, and interactions between country and year are calculated.

Table S18. FEIV models with an interaction of a job with low control

Outcome	Obs.	Variable	Coef.	95% CI		P-value
Cognitive function	161557	Retire	0.054	-0.020	0.127	0.151
		Retire x Low control	-0.030	-0.140	0.079	0.585
Physical independence	161564	Retire	0.003	-0.014	0.021	0.701
		Retire x Low control	0.022	-0.006	0.049	0.125
Self-rated health	161611	Retire	0.149	0.084	0.215	0.000
		Retire x Low control	0.072	-0.028	0.172	0.157
Physical inactivity	144316	Retire	-0.044	-0.073	-0.015	0.003
		Retire x Low control	0.025	-0.019	0.069	0.265
Smoking	131509	Retire	-0.017	-0.040	0.005	0.136
		Retire x Low control	0.039	0.004	0.074	0.028
Binge drinking	97348	Retire	0.009	-0.027	0.044	0.632
		Retire x Low control	-0.022	-0.077	0.033	0.435

Note: All regressions are adjusted for covariates (age, age squared, and marital status), interactions between covariates and a job with low control, and FEs of individual, year, and interactions between country and year. Robust standard errors clustering at individual, year, and interactions between country and year are calculated.

Figure S12. FEIV models for the associations of full-retirement with outcomes

Subgroup	Obs.	Coef. (95% CI)	P-value
**Cognitive Function**			
Men	169243	-0.008 (-0.086, 0.069)	0.838
Women	180496	0.086 (0.024, 0.149)	0.007
**Physical Independence**			
Men	162487	0.024 (0.001, 0.047)	0.042
Women	174231	0.047 (0.028, 0.065)	<0.001
**Self-Rated Health**			
Men	176071	0.123 (0.050, 0.196)	0.001
Women	180681	0.193 (0.136, 0.250)	<0.001
**Physical Inactivity**			
Men	119351	-0.016 (-0.056, 0.025)	0.446
Women	130220	-0.036 (-0.068, -0.005)	0.024
**Smoking**			
Men	149881	0.027 (-0.001, 0.055)	0.063
Women	149878	-0.019 (-0.037, -0.001)	0.035
**Binge Drinking**			
Men	110133	0.031 (-0.011, 0.072)	0.150
Women	113387	-0.007 (-0.033, 0.019)	0.598

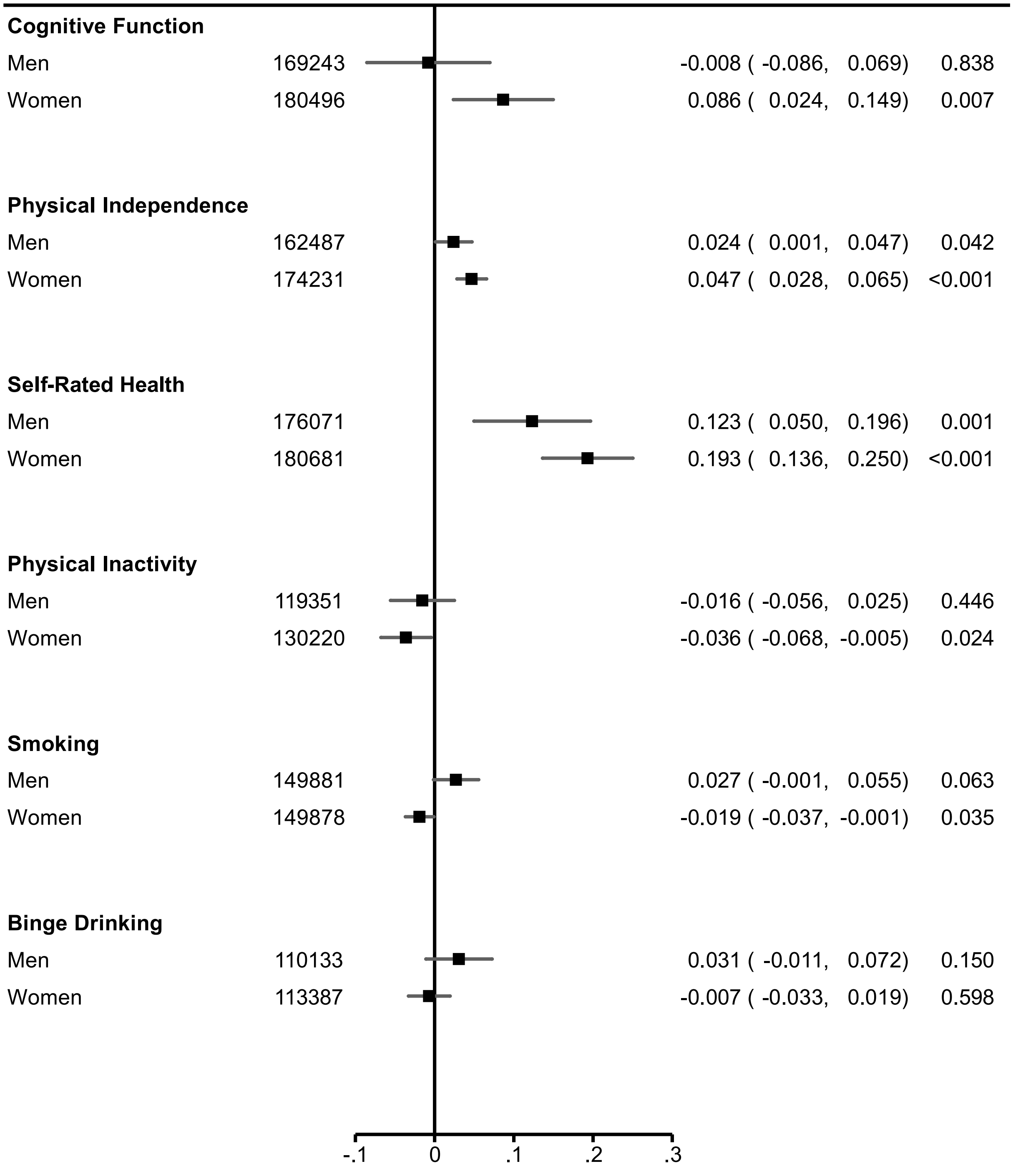

Note: All regressions are adjusted for age, age squared, marital status, and FEs of individual, year, and interactions between country and year. Robust standard errors clustering at individual, year, and interactions between country and year are calculated.

Figure S13. FEIV models for people aged 52–68 years

Subgroup	Obs.	Coef. (95% CI)	P-value
**Cognitive Function**			
Men	154658	-0.106 (-0.192, -0.021)	0.015
Women	162291	0.062 (-0.002, 0.127)	0.058
**Physical Independence**			
Men	147439	0.005 (-0.019, 0.030)	0.673
Women	156429	0.026 (0.007, 0.044)	0.006
**Self-Rated Health**			
Men	161004	0.056 (-0.024, 0.136)	0.170
Women	162712	0.219 (0.160, 0.277)	<0.001
**Physical Inactivity**			
Men	109267	-0.020 (-0.063, 0.023)	0.353
Women	117400	-0.052 (-0.083, -0.022)	0.001
**Smoking**			
Men	136938	0.024 (-0.006, 0.054)	0.123
Women	134843	-0.008 (-0.027, 0.010)	0.371
**Binge Drinking**			
Men	100959	0.038 (-0.008, 0.083)	0.103
Women	102958	-0.001 (-0.029, 0.028)	0.968

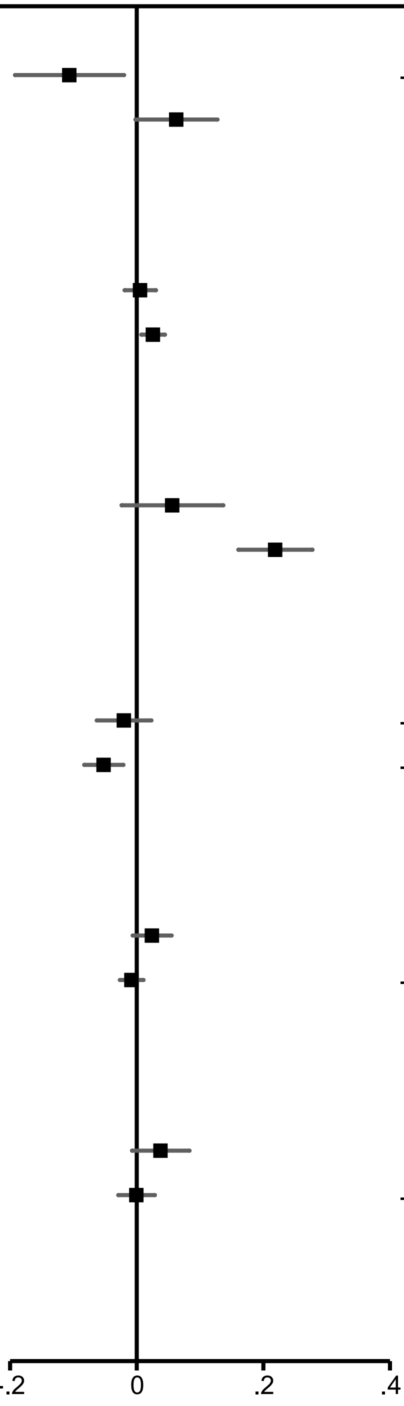

Note: All regressions are adjusted for age, age squared, marital status, and FEs of individual, year, and interactions between country and year. Robust standard errors clustering at individual, year, and interactions between country and year are calculated.

Figure S14. FEIV models for people aged 50–80 years

Subgroup	Obs.	Coef. (95% CI)	P-value
**Cognitive Function**			
Men	262843	0.023 (-0.020, 0.065)	0.299
Women	272819	0.122 (0.082, 0.162)	<0.001
**Physical Independence**			
Men	255133	0.067 (0.054, 0.080)	<0.001
Women	267005	0.083 (0.070, 0.096)	<0.001
**Self-Rated Health**			
Men	274087	0.223 (0.181, 0.265)	<0.001
Women	274535	0.250 (0.213, 0.287)	<0.001
**Physical Inactivity**			
Men	193497	-0.095 (-0.116, -0.075)	<0.001
Women	204254	-0.069 (-0.089, -0.049)	<0.001
**Smoking**			
Men	232335	-0.029 (-0.044, -0.015)	<0.001
Women	228288	-0.028 (-0.040, -0.016)	<0.001
**Binge Drinking**			
Men	171369	-0.013 (-0.035, 0.008)	0.224
Women	172312	-0.013 (-0.028, 0.001)	0.070

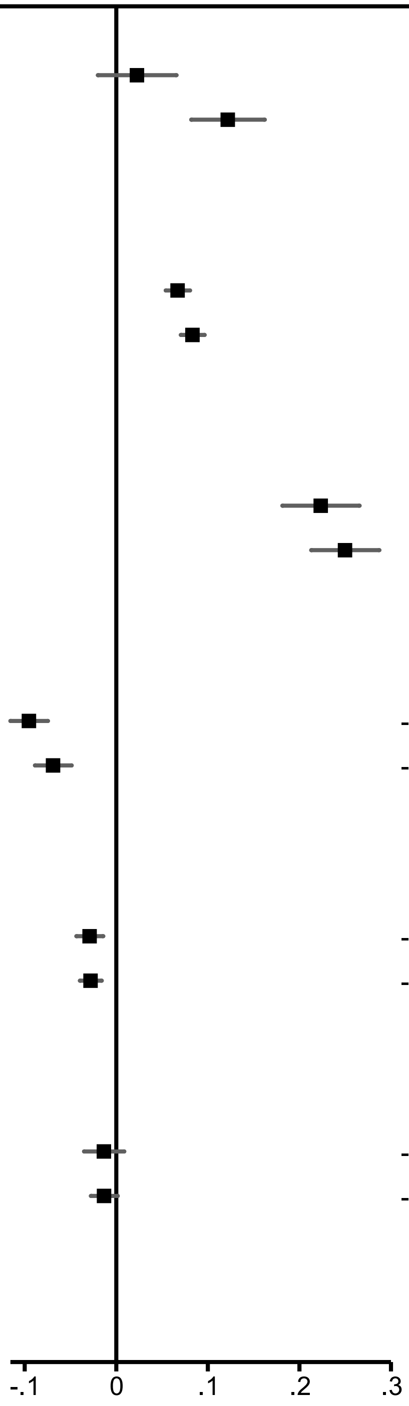

Note: All regressions are adjusted for age, age squared, marital status, and FEs of individual, year, and interactions between country and year. Robust standard errors clustering at individual, year, and interactions between country and year are calculated.

Figure S15. FEIV models excluding self-employed people

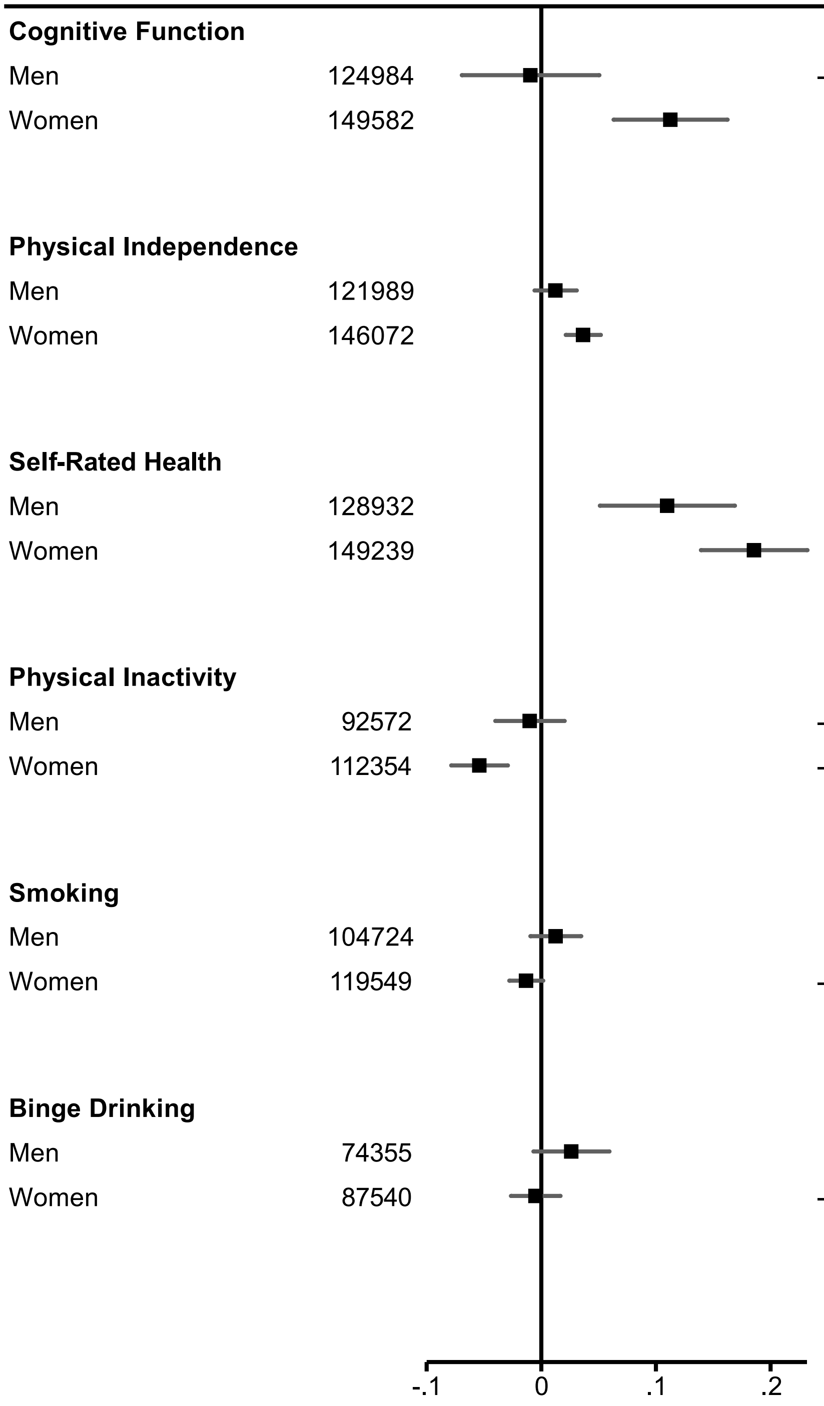

Subgroup	Obs.	Coef. (95% CI)	P-value
**Cognitive Function**			
Men	124984	-0.010 (-0.069, 0.050)	0.754
Women	149582	0.113 (0.063, 0.162)	<0.001
**Physical Independence**			
Men	121989	0.012 (-0.006, 0.030)	0.185
Women	146072	0.036 (0.021, 0.052)	<0.001
**Self-Rated Health**			
Men	128932	0.110 (0.051, 0.168)	<0.001
Women	149239	0.186 (0.139, 0.232)	<0.001
**Physical Inactivity**			
Men	92572	-0.010 (-0.040, 0.020)	0.510
Women	112354	-0.054 (-0.079, -0.030)	<0.001
**Smoking**			
Men	104724	0.012 (-0.009, 0.034)	0.265
Women	119549	-0.013 (-0.028, 0.001)	0.073
**Binge Drinking**			
Men	74355	0.026 (-0.007, 0.059)	0.120
Women	87540	-0.005 (-0.026, 0.016)	0.642

Note: All regressions are adjusted for age, age squared, marital status, and FEs of individual, year, and interactions between country and year. Robust standard errors clustering at individual, year, and interactions between country and year are calculated.

Figure S16. FEIV models excluding countries with weak IVs

Subgroup	Obs.	Coef. (95% CI)	P-value
**Cognitive Function**			
Men	163265	-0.003 (-0.063, 0.057)	0.925
Women	179253	0.093 (0.043, 0.144)	<0.001
**Physical Independence**			
Men	154500	0.008 (-0.009, 0.025)	0.363
Women	172054	0.038 (0.022, 0.053)	<0.001
**Self-Rated Health**			
Men	167887	0.084 (0.029, 0.140)	0.003
Women	178222	0.199 (0.153, 0.244)	<0.001
**Physical Inactivity**			
Men	113877	-0.034 (-0.061, -0.008)	0.011
Women	129013	-0.041 (-0.065, -0.018)	0.001
**Smoking**			
Men	143229	0.011 (-0.009, 0.031)	0.281
Women	148777	-0.018 (-0.033, -0.003)	0.017
**Binge Drinking**			
Men	105993	0.025 (-0.004, 0.055)	0.090
Women	112413	-0.002 (-0.023, 0.018)	0.821

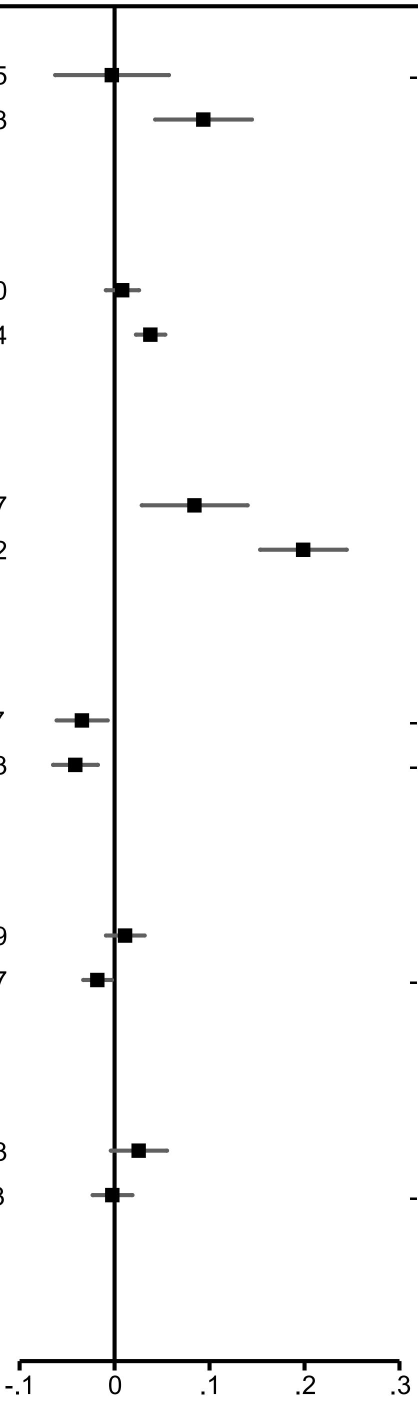

Note: All regressions are adjusted for age, age squared, marital status, and FEs of individual, year, and interactions between country and year. Robust standard errors clustering at individual, year, and interactions between country and year are calculated.

Figure S17. FEIV models excluding data from the United States

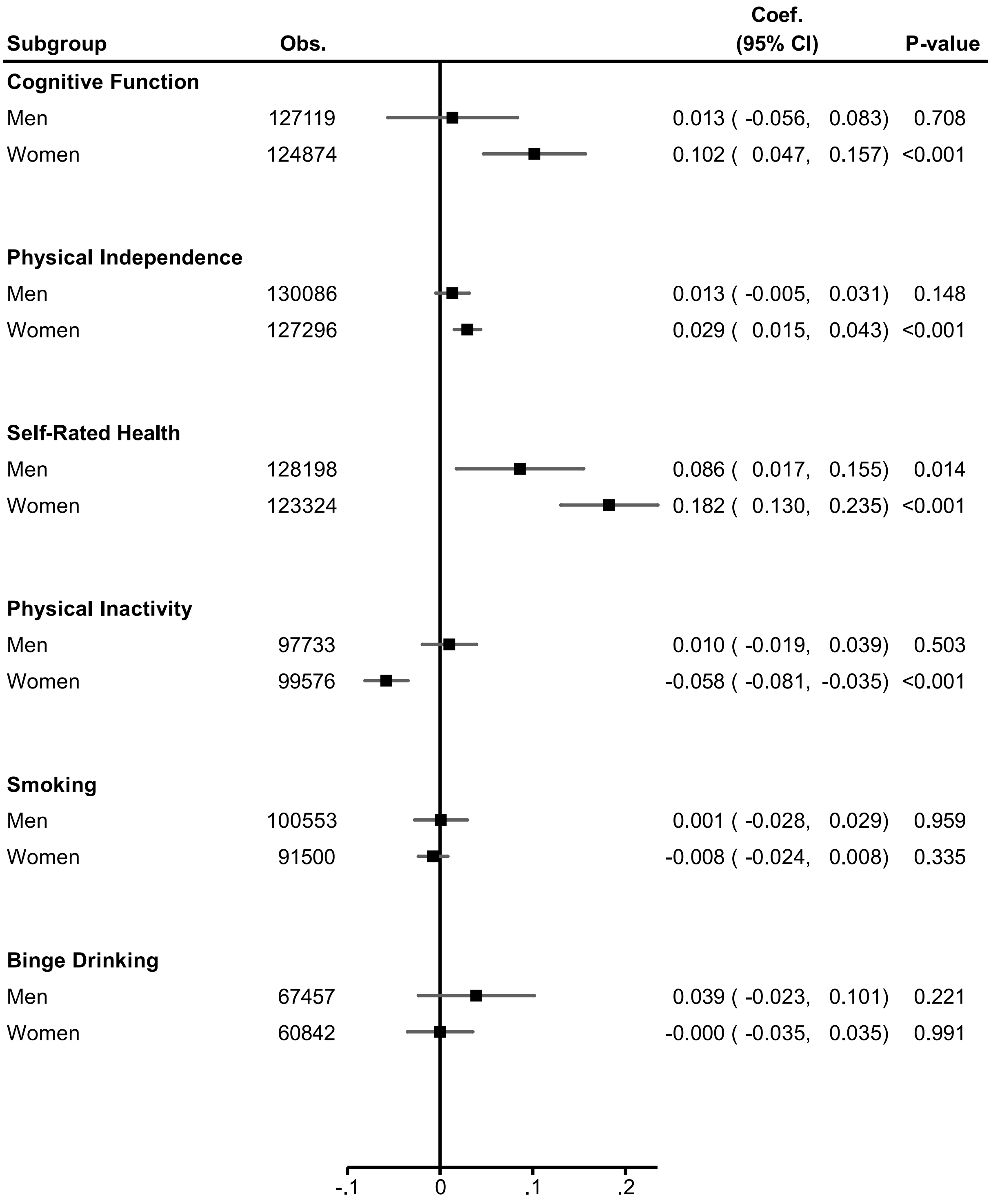

Note: All regressions are adjusted for age, age squared, marital status, and FEs of individual, year, and interactions between country and year. Robust standard errors clustering at individual, year, and interactions between country and year are calculated.

Figure S18. FEIV models by gender and retirement duration

Subgroup	Obs.	Coef. (95% CI)	P-value
**Cognitive Function**			
Men, Retired <5 years	138647	-0.022 (-0.093, 0.048)	0.534
Men, Retired ≥5 years	144989	-0.003 (-0.115, 0.109)	0.957
Women, Retired <5 years	138731	0.074 (0.009, 0.139)	0.026
Women, Retired ≥5 years	155197	0.080 (-0.001, 0.161)	0.053
**Physical Independence**			
Men, Retired <5 years	132244	-0.003 (-0.023, 0.017)	0.747
Men, Retired ≥5 years	139205	0.046 (0.012, 0.079)	0.007
Women, Retired <5 years	132566	0.029 (0.012, 0.047)	0.001
Women, Retired ≥5 years	149693	0.053 (0.028, 0.078)	<0.001
**Self-Rated Health**			
Men, Retired <5 years	145256	0.101 (0.031, 0.171)	0.005
Men, Retired ≥5 years	151834	0.083 (-0.025, 0.190)	0.132
Women, Retired <5 years	139710	0.211 (0.150, 0.272)	<0.001
Women, Retired ≥5 years	155621	0.175 (0.099, 0.252)	<0.001
**Physical Inactivity**			
Men, Retired <5 years	94355	0.047 (0.006, 0.088)	0.026
Men, Retired ≥5 years	99389	-0.043 (-0.108, 0.023)	0.203
Women, Retired <5 years	95769	-0.038 (-0.075, -0.000)	0.047
Women, Retired ≥5 years	109222	-0.024 (-0.069, 0.021)	0.289
**Smoking**			
Men, Retired <5 years	123937	0.026 (-0.002, 0.053)	0.066
Men, Retired ≥5 years	128597	0.034 (-0.013, 0.081)	0.159
Women, Retired <5 years	115386	-0.007 (-0.027, 0.012)	0.464
Women, Retired ≥5 years	127466	-0.018 (-0.044, 0.008)	0.173
**Binge Drinking**			
Men, Retired <5 years	92173	0.039 (-0.001, 0.080)	0.058
Men, Retired ≥5 years	95198	0.025 (-0.048, 0.098)	0.501
Women, Retired <5 years	88707	-0.006 (-0.034, 0.021)	0.652
Women, Retired ≥5 years	96467	-0.006 (-0.048, 0.036)	0.780

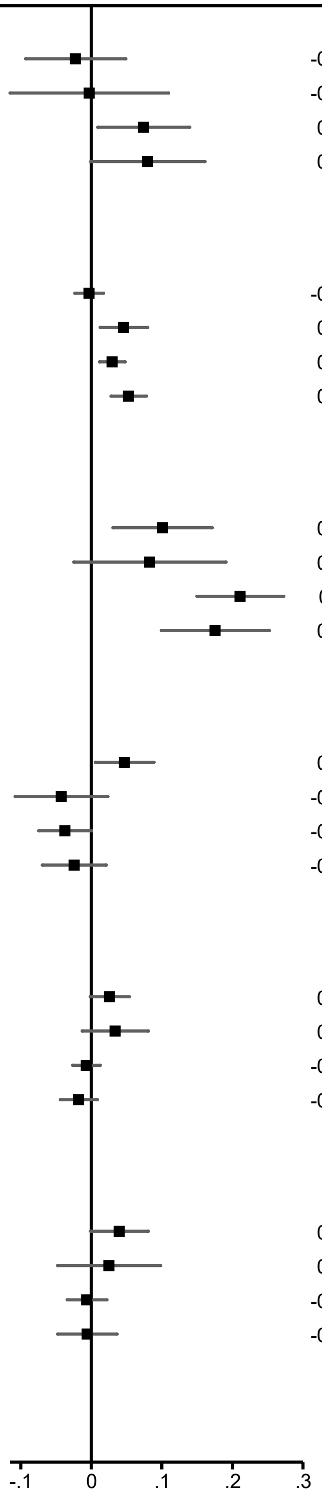

Note: All regressions are adjusted for age, age squared, marital status, and FEs of individual, year, and interactions between country and year. Robust standard errors clustering at individual, year, and interactions between country and year are calculated.

Figure S19. FEIV models by region and retirement duration

Subgroup	Obs.	Coef. (95% CI)	P-value
**Cognitive Function**			
Europe, Retired <5 years	138950	0.046 (-0.003, 0.095)	0.066
Europe, Retired ≥5 years	153978	0.045 (-0.015, 0.105)	0.144
America, Retired <5 years	114260	0.021 (-0.075, 0.117)	0.664
America, Retired ≥5 years	120219	-0.027 (-0.226, 0.171)	0.790
Asia, Retired <5 years	24168	0.014 (-0.424, 0.452)	0.950
Asia, Retired ≥5 years	25989	0.164 (-0.481, 0.809)	0.618
**Physical Independence**			
Europe, Retired <5 years	142392	0.019 (0.007, 0.031)	0.002
Europe, Retired ≥5 years	157784	0.033 (0.016, 0.049)	<0.001
America, Retired <5 years	96246	0.002 (-0.035, 0.040)	0.911
America, Retired ≥5 years	102925	0.096 (0.016, 0.176)	0.018
Asia, Retired <5 years	26172	-0.005 (-0.154, 0.144)	0.949
Asia, Retired ≥5 years	28189	0.058 (-0.143, 0.259)	0.570
**Self-Rated Health**			
Europe, Retired <5 years	137475	0.208 (0.161, 0.255)	<0.001
Europe, Retired ≥5 years	152391	0.117 (0.059, 0.175)	<0.001
America, Retired <5 years	120967	0.043 (-0.038, 0.124)	0.295
America, Retired ≥5 years	127567	0.127 (-0.038, 0.292)	0.132
Asia, Retired <5 years	26524	-0.814 (-1.568, -0.059)	0.035
Asia, Retired ≥5 years	27497	-0.245 (-1.154, 0.664)	0.597
**Physical Inactivity**			
Europe, Retired <5 years	113289	-0.019 (-0.042, 0.003)	0.092
Europe, Retired ≥5 years	125329	-0.051 (-0.083, -0.020)	0.001
America, Retired <5 years	55520	-0.043 (-0.142, 0.056)	0.397
America, Retired ≥5 years	61402	-0.086 (-0.216, 0.043)	0.190
Asia, Retired <5 years	21315	0.176 (-0.166, 0.518)	0.312
Asia, Retired ≥5 years	21880	-0.052 (-0.470, 0.366)	0.808
**Smoking**			
Europe, Retired <5 years	89570	0.001 (-0.019, 0.020)	0.957
Europe, Retired ≥5 years	97905	-0.009 (-0.033, 0.015)	0.471
America, Retired <5 years	122260	0.006 (-0.019, 0.031)	0.624
America, Retired ≥5 years	128810	-0.013 (-0.064, 0.038)	0.625
Asia, Retired <5 years	27493	-0.046 (-0.172, 0.080)	0.473
Asia, Retired ≥5 years	29349	-0.124 (-0.287, 0.038)	0.134
**Binge Drinking**			
Europe, Retired <5 years	55312	0.009 (-0.027, 0.045)	0.612
Europe, Retired ≥5 years	59298	0.015 (-0.044, 0.073)	0.626
America, Retired <5 years	103570	0.012 (-0.011, 0.035)	0.290
America, Retired ≥5 years	110655	0.016 (-0.025, 0.058)	0.444
Asia, Retired <5 years	21998	0.112 (-0.294, 0.518)	0.590
Asia, Retired ≥5 years	21712	0.010 (-0.423, 0.442)	0.965

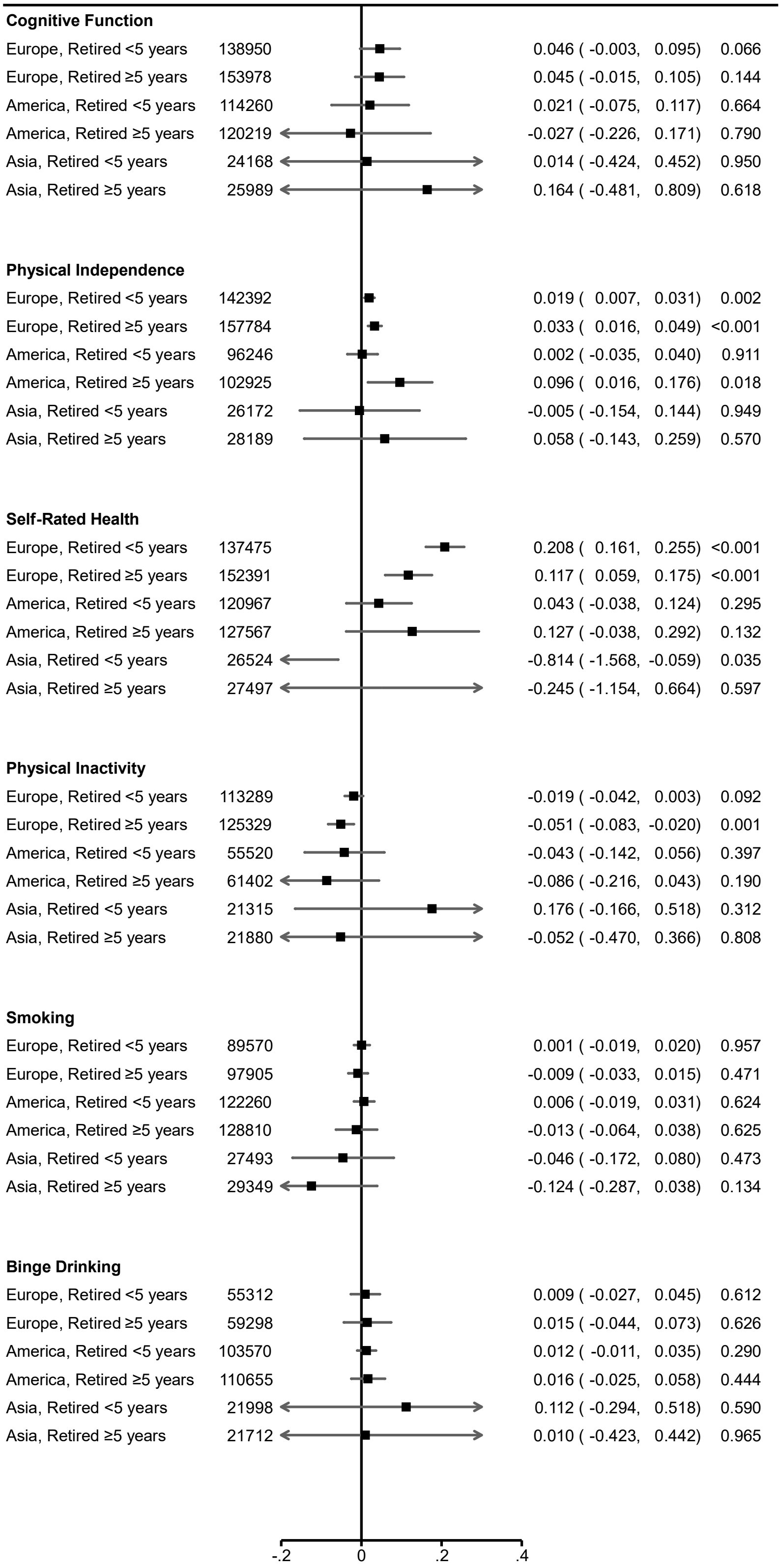

Note: All regressions are adjusted for age, age squared, marital status, and FEs of individual, year, and interactions between country and year. Robust standard errors clustering at individual, year, and interactions between country and year are calculated.

Figure S20. FEIV models with further adjustments

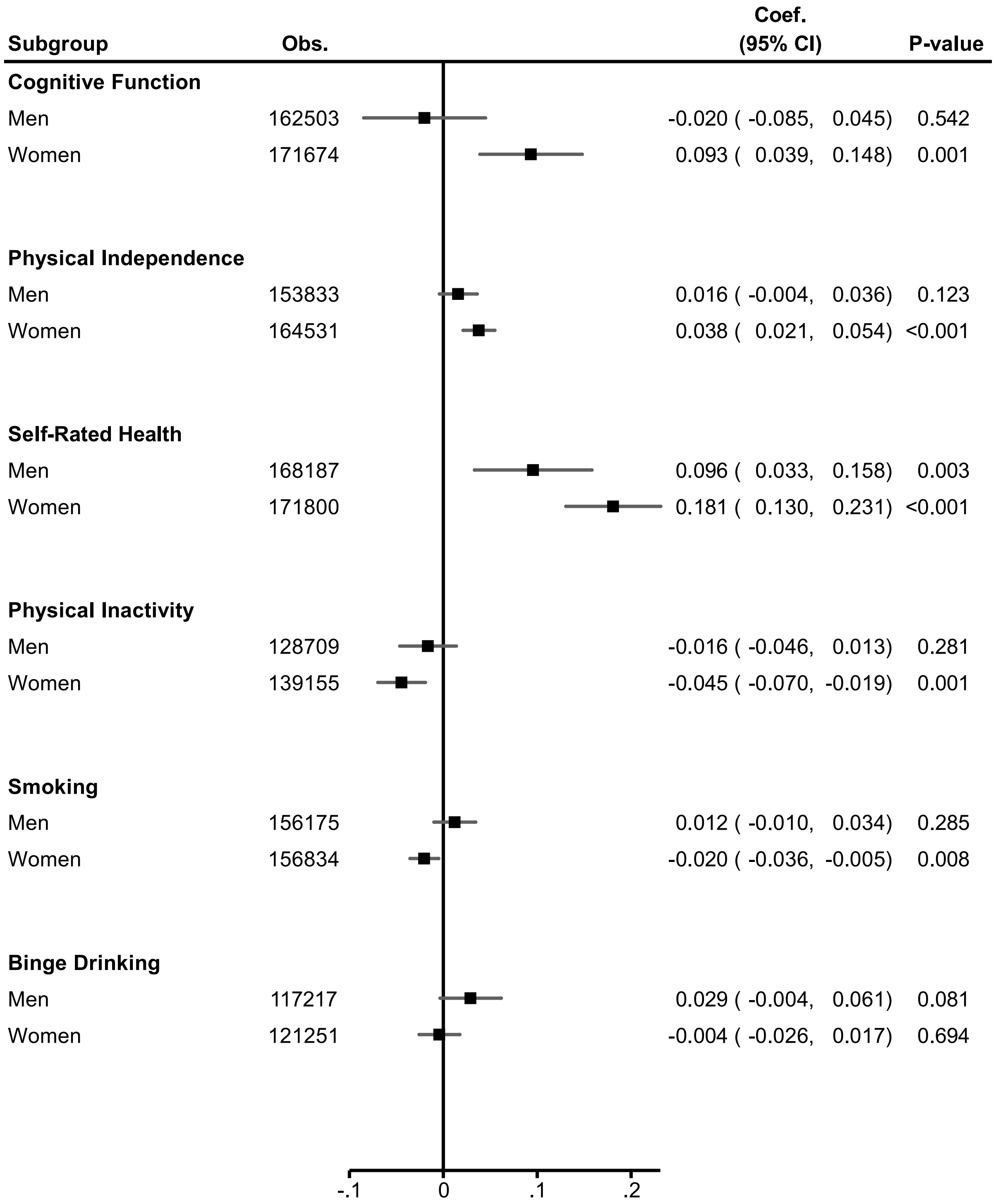

Note: All regressions are adjusted for age, age squared, marital status, household assets, heart disease, stroke, and FEs of individual, year, and interactions between country and year. The variable of household assets is the net value of assets at the couple-level unit calculated as the value of all wealth components (including housing, financial, and non-financial assets) minus that of all debts. To make the variables in different surveys comparable, we standardized them to z-scores for each survey. The variables of heart disease and stroke indicate 1 if a doctor has ever told the participant that they have the conditions and 0 otherwise. Robust standard errors clustering at individual, year, and interactions between country and year are calculated.

Table S19. FEIV models for the raw scores of cognitive function

	Obs.	Coef.	95% CI		P-value
Men	147,516	-0.089	-0.286	0.109	0.380
Women	168,730	0.281	0.111	0.451	0.001

Note: All regressions are adjusted for age, age squared, marital status, and FEs of individual, year, and interactions between country and year. Robust standard errors clustering at individual, year, and interactions between country and year are calculated.
